# Toward transgenic multicellular parasites: CRISPR-Cas12a germline editing of *Schistosoma mansoni* eggs as key to success

**DOI:** 10.1126/sciadv.aeh0455

**Published:** 2026-09-30

**Authors:** Max F. Moescheid, Prapakorn Wisitpongpun, Vanessa Gross, Victoria H. Mann, Thomas Quack, Anne Rognon, Christoph Grunau, Wannaporn Ittiprasert, Paul J. Brindley, Christoph G. Grevelding

**Affiliations:** ^1^ Institute of Parasitology, Biomedical Research Center Seltersberg (BFS), Justus Liebig University Giessen, Germany.; ^2^ Department of Microbiology, Immunology & Tropical Medicine, School of Medicine & Health Sciences, George Washington University, Washington, DC 20037, USA.; ^3^ Cellular and Molecular Immunology Research Unit (CMIRU), Faculty of Allied Health Sciences, Naresuan University, Phitsanulok 65000, Thailand.; ^4^ IHPE Univ Perpignan Via Domitia, CNRS, IFREMER, Univ Montpellier, Perpignan, France.

## Abstract

Lack of transgenesis methods impedes functional studies in platyhelminths. We present a substantially improved ribonucleic protein complex (RNP)–based genome editing approach for *Schistosoma mansoni* comparing Cas9 and Cas12a, the latter attained more efficient homology-directed insertion of a 5′-C6-PEG10–modified double-stranded donor template bearing 50-nucleotide microhomology arms into a genome safe-harbor site (GSH1). Electroporation of eggs recovered from host livers resulted in enhanced green fluorescent protein (EGFP) reporter activity, driven by an *S. mansoni* ubiquitin promoter and terminator. Hamster infection with transgenic cercariae, the infectious stage of this parasitic platyhelminth, recovered from miracidial infection of snails, or from sporocysts generated in vitro from transgenic miracidia and implantation in snails, produced EGFP-positive adult worms. Sequencing of the edited GSH1 and analyses of filial generations demonstrated chromosomal integration and the stable transformation of *S. mansoni*. Together, this approach advances schistosome transgenesis and may be pioneering for other platyhelminths, which lack functional genomics and transformation methods.

## INTRODUCTION

CRISPR-Cas–mediated genome editing has revolutionized research in biomedicine and the life sciences generally (*1*, *2*) and has recently been established for the intermediate host of *Schistosoma mansoni*, *Biomphalaria glabrata* (*3*). An increasing repertoire of Cas homologs is available today, having divergent programmed nuclease activities including alternative protospacer adjacent motifs (PAMs), selectivity for divergent nucleic acid substrates, and sensitivity to metal ions, to temperature, and other factors (*4*, *5*). CRISPR-Cas9 ribonucleic protein complexes (RNPs) can consist of two components: a single guide RNA (gRNA) and Cas9 nuclease (*6*–*8*). Cas9 induces blunt-end double-strand breaks (DSBs) within a targeted locus, which can be repaired by genome maintenance mechanisms including nonhomologous end joining (NHEJ) and homology-directed repair (HDR). In contrast, CRISPR-Cas12 components consist of a CRISPR RNA (crRNA) and a Cas12a nuclease inducing staggered-end DSBs (*9*). CRISPR-Cas9 recognizes the NGG PAM to catalyze the DSB upstream of PAM, whereas CRISPR-Cas12a recognizes a T-rich PAM, TTTV, with the DSB generated downstream of its PAM (*10*). Both nucleases have utility for gene editing in discrete genomic contexts; Cas9 may have advantages for editing GC-rich regions, whereas Cas12a may be better suited for editing AT-rich regions (*11*, *12*).

To establish CRISPR-Cas–mediated genome editing in the parasitic platyhelminth *S. mansoni*, our initial work identified a genome safe harbor (GSH) site, GSH1. On the basis of an independent study (*13*), we defined this region as free of overlapping functional genomic elements and without heterochromatic marks potentially impeding transcription (*14*, *15*). Next, we selected *S. mansoni* eggs extracted from host livers as a biological substrate for editing using electroporation (EPO) for delivery of CRISPR. We used three overlapping gRNAs complexed with the Cas9 nuclease from *Streptococcus pyogenes* to insert a reporter gene construct into GSH1 (*14*, *15*). After we confirmed that Cas12a from *Acidaminococcus* sp. (AsCas12a) was also enzymatically active in *S. mansoni* eggs (*16*), we compared both Cas homologs for ability to edit GSH1 as a prerequisite to generate a stably transformed *S. mansoni* line. Liver-stage eggs (LEs) served as the biological targets. LEs represent a heterogeneous population at different stages of development (*17*), and they are available from liver and intestines of experimentally infected rodents. This increases the probability of transgene integration events being passed on to the next generation and makes LEs an attractive target for germline transformation approaches, followed by selection based on the expression of a reporter gene. To optimize editing efficiency, we considered the design of a single gRNA in tandem with short, 5′-modified homology arms (HAs) of the repair template.

Our findings demonstrated a significantly advanced genome editing approach for *S. mansoni*, which resulted in hitherto unprecedented gene editing efficiency using Cas12a. We present compelling evidence that selection of transgenic schistosomes at the sporocyst stage is possible based on reporter gene expression. Moreover, we provide evidence for two routes to generate stably transformed schistosome lines, by continuing the life cycle the “biological” (vertical) way, where the intermediate snail host is infected by transgenic miracidia, and a “surgical” (horizontal) route of transplantation of sporocysts, generated from transgenic miracidia in vitro, into recipient snails. Both routes resulted in transgenic cercariae and, in turn, transgenic adult *S. mansoni*, which maintained the transgene at GSH1 and its expression in the subsequent generation.

## RESULTS

### Promoter and terminator elements of the endogenous ubiquitin gene drive episomal EGFP expression in adult *S. mansoni*

Reporter gene design to establish chromosomally modified schistosomes requires a selectable marker gene under the control of regulatory elements, which are active in the desired way. To identify a promoter ensuring constitutive and stage-independent reporter gene expression, we interrogated transcriptomics data available for *S. mansoni* (*18*–*20*), wherein we observed that the gene Smp_335990 was strongly, ubiquitously, and stage-independently expressed (fig. S1). Its annotation in WormBase ParaSite (*21*) identified this gene as ubiquitin-like domain-containing protein-coding gene (A0A5K4F9I1; UniProt), which we confirmed using the protein structure prediction tool Phyre2 (*22*). The prediction indicated that Smp_335990 is an ortholog of *ubb*, the human ubiquitin gene (*23*) (fig. S2). Next, to identify promoter elements known to be necessary for transcription and translation initiation, which could be likewise important for driving transgene expression, we analyzed the 2056–base pair (bp) region upstream of the coding sequence of Sm*ubi* (Fig. 1A). Here, we observed canonical promoter and enhancer elements (tables S1 to S3) (*24*, *25*). Promoter analysis, applying the Neural Network Promoter Prediction tool (*24*), predicted two core promoter regions: the first at position 1981 to 2030 bp and the second at 156 to 205 bp (table S3). Subsequently, these regions were analyzed in more detail via AliBaba2.1 (*26*, *27*), TRANSFAC 4.0 (*26*), and YAPP eukaryotic core promoter predictor (*26*, *28*–*30*) and by comparison with known consensus promoter elements (*31*–*33*) (tables S2 and S3). In the core promoter rank 1, typical sequence elements needed for transcription initiation and regulation were present.

**Fig. 1.**
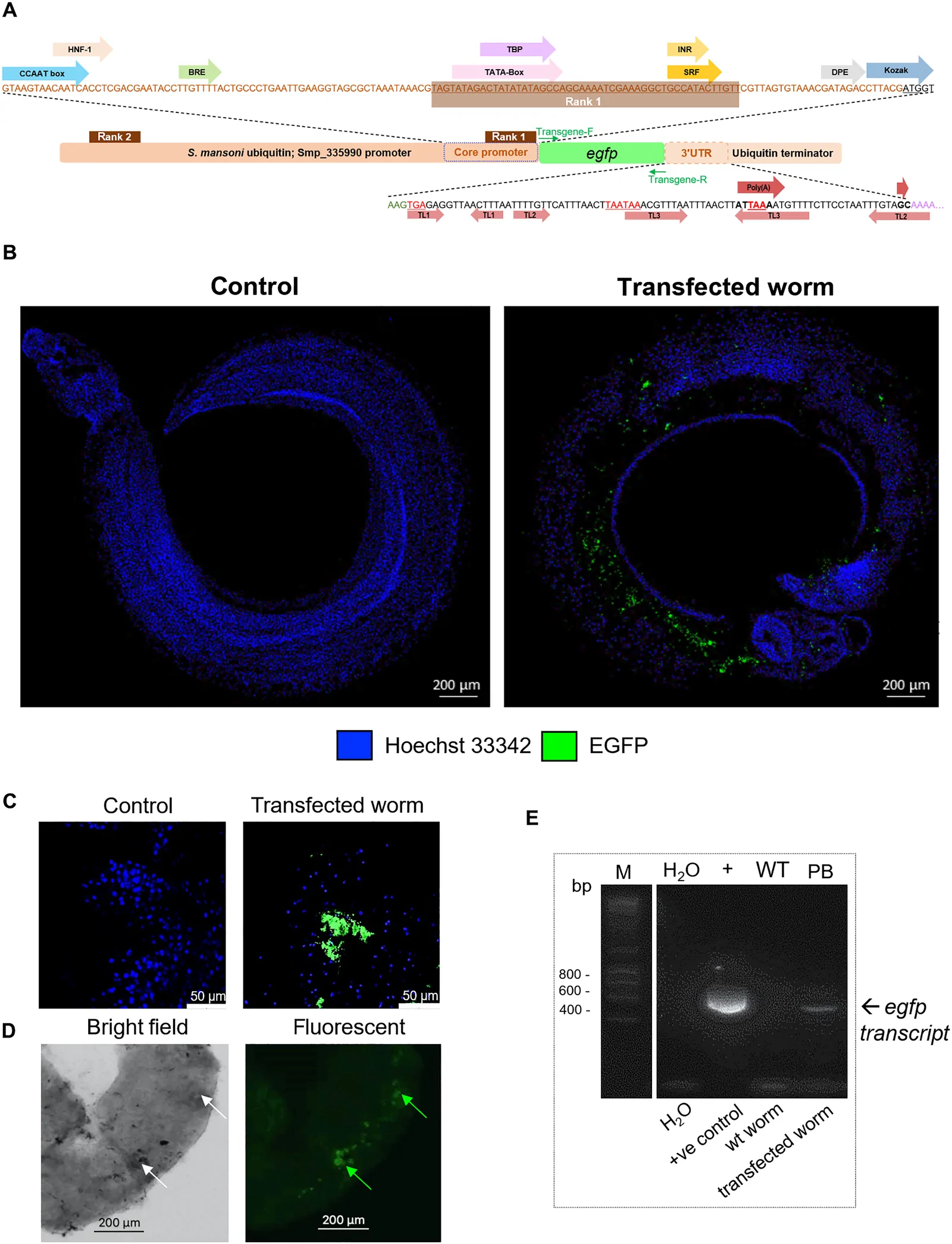
Bioinformatic prediction and experimental verification of the regulatory activity of the *S. mansoni* ubiquitin (Sm*ubi*) promoter and terminator elements driving EGFP expression. (**A**) Overview of the Sm*ubi* (Smp_335990) promoter and terminator elements (see also tables S1 and S2). Elements were identified using the Berkeley *Drosophila* Genome Project Neural Network PromoterPrediction Tool, AliBaba2.1 TRANSFAC 4.0 (*26*) and YAPP eukaryotic core promoter predictor (*26*, *28*–*30*), respectively. Two core promoters were predicted (rank 1 and rank 2; brown boxes) containing a CCAAT enhancer-binding site, hepatocyte nuclear factor 1 binding element (NHF-1), B recognition element (BRE), TATA box–containing region (TBP), transcription initiator element (INR), serum response factor (SRF), and downstream promoter element (DPE) (colored arrows in the enlarged top part). The presumptive terminator region (bottom) contains three presumptive terminator stem-loop structures (TL1 to TL3) and the polyadenylation signal [Poly(A)], which were predicted by the RNAfold Webserver Vienna tools (*37*, *149*) and the DNAFSMiner-DNA Functional Site Miner polyadenylation side prediction tool (*30*), respectively. The Sm*ubi* promoter–induced EGFP reporter gene expression was monitored in male *S. mansoni*, which had been transfected by PB (*39*, *40*) with the SmUbi-EGFP-SmUbi vector construct. After 48 h, transfected as well as untreated control worms were counterstained with Hoechst 33342 (blue) and analyzed by CLSM (**B** and **C**). Green signals were observed by fluorescence microscopy, whereas no EGFP signal were found in control worms. (**D**) As expected, bright-field microscopy also confirmed the presence of clusters of gold particles in the tissue (white arrows). EGFP signals matched the location of gold particles (green arrows). (**E**) RT-PCR confirmed the presence of *egfp* transcripts in transfected worms (PB) and the positive control (+; plasmid DNA as a template). No *egfp* transcripts occurred in control WT worms or in a control without template (H_2_O). Created in BioRender. Möscheid, M. (2026) https://BioRender.com/px43xta.

Furthermore, we identified the start codon-containing Kozak sequence required for ribosome binding and translation initiation (*34*) in silico by comparing the potential Kozak sequences of *S. mansoni* genes known to be abundantly transcribed and sharing comparable transcription strengths (*34*–*36*) and expression patterns (*19*, *35*) to that of Sm*ubi*. For these genes, we predicted the consensus sequence 5′-N N (T/C) (A/G) N (A/G/T) ATG N (C/A/T) N-3′ 6 bp downstream and 5 bp upstream of the translation start (table S2).

Next, we identified the potential terminator sequences including the 3′ untranslated region (3′UTR) (*3*, *5*) of Sm*ubi* using the DNAFSMiner prediction tool (Fig. 1A and table S3) (*37*, *38*). After cloning of the 5′-SmUbi-EGFP-SmUbi-3′ reporter gene construct, we confirmed its suitability by transfecting adult male schistosomes with particle bombardment (PB) (Fig. 1, B to D), a ballistic gene transfer method suitable for the transient transformation of adult and larval schistosomes (*39*, *40*). In contrast to control worms, only worms transfected with this reporter gene showed episomal enhanced fluorescent protein (EGFP) expression. Reverse transcription polymerase chain reaction (RT-PCR) analysis also confirmed the expression of EGFP (Fig. 1E and fig. S3).

### Programmed editing of GSH1 in the schistosome egg

The *S. mansoni* genome is AT-rich (*41*), as is the GSH1 (AT: 65.82%), which constrains the application of the GC content–dependent Cas9 for editing, whereas Cas12a is better suited for editing AT-rich regions (*42*). Our previous studies proved that a combination of three overlapping gRNAs combined with Cas9 significantly increased the number of deleted base pairs at the target locus (*14*, *15*). This increased donor DNA integration by HDR by up to 70% into LEs. We hypothesized that DNA cleavage by Cas12a combined with a single crRNA might result in staggered DNA ends, improving the editing efficiency over that obtained with Cas9, leading to higher indel frequencies mediated by NHEJ and HDR combined with a single gRNA. To this end, we designed crRNAs of both Cas12a and Cas9 with the same programmed cleavage site (Fig. 2A).

**Fig. 2.**
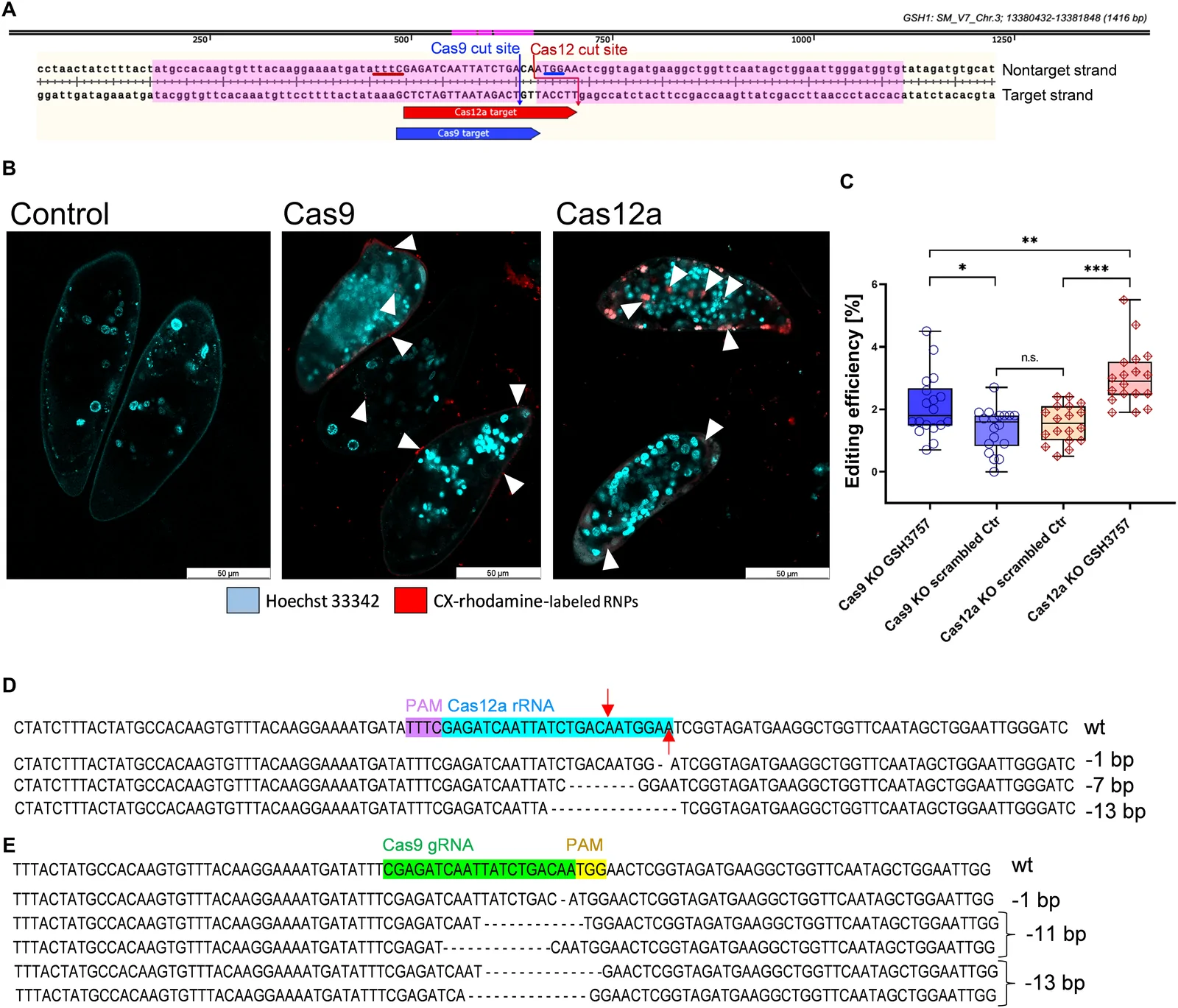
RNA-guided AsCas12a- and SpCas9-catalyzed editing of GSH1 in adult schistosomes and their eggs. Synoptic overview of the comparative approach using Cas9 and Cas12a for programmed gene editing using *S. mansoni* eggs and adults as biological targets for EPO-based transformation. (**A**) Schematic overview of the *S. mansoni* GSH1 target sites for Cas9 (blue arrow) and Cas12a (red arrow). DNA cleavage by Cas9 was expected to result in blunt-ended DNA strands, whereas Cas12a was expected to cause the staggered cleavage of the DNA strand. (**B**) LEs were subjected to EPO in the presence of CX-rhodamine–labeled RNPs (red). LEs were counterstained with Hoechst 33342 (blue) and examined by CLSM 24 hours later. The introduction of RNPs was demonstrated (arrows) with both nucleases. Most Cas9 RNPs accumulated around the eggshell, a pattern dissimilar to Cas12a RNP–treated LEs, where signals were also found within the egg. TIDE analysis of 18 independently electroporated egg pools (**C**) confirmed the successful deletion of the target sequence in the schistosomal GSH1 locus applying RNPs derived from Cas9 (blue) and Cas12a (red). Arrows point to very small, CX-rhodamine spots indicating minor amounts of Cas9 RNPs. We found negligible to low background activity (low mutation rate) with RNPs containing a scrambled gRNA sequence (faint blue and orange). Analysis of NGS data of both LEs (**D**) and adult schistosomes (**E**) showed significantly enhanced editing seen with Cas12a RNPs (red arrows) than Cas9 RNPs. Abbreviations: CLSM, confocal laser scanning microscopy; Ctr, control; KO, knockout; NGS, next-generation sequencing; n.s., not significant. *P < 0.05, **P < 0.01, and ***P < 0.001 by a two-sided *t* test.

Furthermore, we selected gRNAs sharing the same predicted CRISPR-Cas efficiency and for which no off-targets were predicted. We determined the accessibility of LEs for RNPs of both enzymes using RNP tracking with fluorescently labeled RNPs. For this, each gRNA (sgRNA of Cas9 or crRNA of Cas12a) was labeled with a CX-rhodamine fluorescent tag before the assembly of RNP complexes with either *S. pyogenes* Cas9 (SpCas9) or *Acidaminococcus* sp. Cas12a (AsCas12a) nucleases (*16*, *43*, *44*). Subsequently, LEs were transformed with the RNPs by square-wave EPO. We monitored the entry of RNPs entry into the schistosome eggs by confocal laser scanning microscopy (CLSM). By 24 hours following EPO, RNPs were seen in the emerging larval tissue and colocalization with Hoechst 33342–stained nuclei for both Cas nucleases (Fig. 2B). For Cas9 RNPs, a similar picture emerged; however, comparably less labeled RNPs were evident in larval cells. These findings indicated that, although both RNPs were successfully delivered into the embryo/larva, most Cas9 RNPs accumulated close to or in the eggshell, whereas a wider signal distribution including within the larva developing larva within the eggshell was obtained in Cas12a RNP–treated LEs.

Next, we transfected LEs with unlabeled RNPs of Cas9 and Cas12a to compare their potency to access and edit GSH1. Ten days after EPO, we extracted gDNA for PCR to generate templates for indel analysis by Sanger direct sequencing followed by tracking of indels by decomposition (TIDE) analysis (*16*, *45*). We used TIDE to determine the percentage of mismatching base pairs from a chromatogram following Sanger direct sequencing of RNP-treated samples compared to wild-type (WT) controls. The obtained TIDE value compiled substitution and deletion events at the target locus and represented the locus-specific editing efficiency. From 18 independent biological replicates, editing efficiencies were 2.2 ± 0.9 and 3.0 ± 0.9% for Cas9 RNP and Cas12a RNP, respectively. The WT (untreated), the EPO control (mock), and LEs treated with RNPs formed by a crRNA with a scrambled sequence (table S4) were included for this study to investigate background mutations and CRISPR-Cas off-target activity on GSH1. After normalizing amplified sequences to the background noise, an artifact of the sequencing procedure, we subtracted the editing efficiency of the experimental groups (RNP treatment) with the values obtained for WT and mock controls (∼0.1%) to adjust the editing efficiencies. The mutation rate in control group LEs treated with RNPs containing crRNA with a scrambled crRNA sequence was 1.4 ± 0.7%, significantly lower than Cas9- and Cas12a-programmed GSH1 cleavage (Fig. 2C).

Furthermore, we pooled isolated gDNAs (after TIDE analysis above) from sample nos. 1–3, 4–6, and 8–9 for target amplicon next-generation sequencing (NGS). NGS paired-end reads (∼100,000 reads of each sample) from three independent NGS libraries were analyzed by both CRISPRessV2 and Rgen Tools, confirming the precise GSH1 editing by both enzymes (Fig. 2, D and E) (*46*–*48*). We revealed nucleotide deletion on the expected cleavage site showing deletions of 1, 7, and 13 bp by Cas12a and 1, 11, and 13 bp by Cas9 (Fig. 2, D and E). The GSH1 indel profiles were similar from three NGS libraries (fig. S4, A and B). Comparing Cas9/12a editing efficiencies of the GSH1 locus, we found a 100-fold higher editing efficiency for Cas12a (fig. S4B). The length of deletions at GSH1 ranged from 9 to 18 nucleotides (nt) in the Cas12a-treated group to 1 nt for Cas9 (fig. S4B). These findings demonstrated the capability of successful genome editing for both Cas nucleases in *S. mansoni* eggs, although with notable efficiency bias toward Cas12a.

### Microhomology arms allowed precise integration of the transgene

Next, we investigated whether both enzymes equally enable transgene integration into GSH1 resulting from CRISPR-mediated HDR. We focused on the use the 5′ modifications of a double-stranded DNA (dsDNA) donor as a template for transgene KI into GSH1 with amine C6 and PEG10 treatment at the 5′ termini of DNA donor containing HAs of 50 nt. In an independent study, 5′-C6 amine-PEG10 (C6-PEG10)–modified donors (with ∼50-nt HA) showed highest HDR efficiencies to edit a target gene of human embryonic kidney (HEK) 293 cells with single gRNAs (*49*). Here, we investigated the utility of PCR amplicons as donor templates for knock-in of the SmUbi-EGFP-SmUbi construct into GSH1 catalyzed by Cas9 and Cas12a. Transgene integration was achieved at ratios of 1:1:1 (w/w/w) of enzyme:Cas9 sgRNA or Cas12a crRNA:donor dsDNA template. The 5′ HA of the donor template contained the Cas12a PAM recognition site (TTTC) and a partial proportion of the Cas12a target sequence. One base of the Cas12a PAM TTTC was modified to TCTC, to both eliminate the Cas12a PAM and off-target activity, and to obviate donor-DNA cleavage by Cas12a RNPs. In addition, the design of the gRNAs and selection of the corresponding PAM recognition sites of both nucleases accomplished transgene integration by using the same HA sequences of the donor template targeting GSH1. This was achieved by the selection of predicted and almost identical cleavage sites of both nucleases at GSH1 (Fig. 2A and fig. S4A). For transfection of LEs with SmUbi-EGFP-SmUbi, we coelectroporated RNPs and donor template. Mock WT electroporated LEs (mock) served as control, which we transfected with the donor template in the absence of RNP. Ten days post-EPO, we collected eggs and isolated gDNA for the analysis of transgene integration (Fig. 3). For this, we designed two primer pairs amplifying (i) the 5′-integration site including sequences within the GSH1 as well in the Sm*ubi* promoter region of the transgene (5′-KI; Fig. 3B) and (ii) amplifying the 3′-integration site including the 3′ sequence of the *egfp* and GSH1 (3′-KI; Fig. 3B). With both primer pairs, we amplified 5′-KI and 3′-KI integration fragments and thus demonstrated GSH1 editing and KI by both nucleases (Fig. 3C). In comparison to the amplicons generated by Cas12a-treated LEs, the band intensity of 5′-KI amplicons in the Cas9 group was comparatively lower (Fig. 3C). Nucleotide sequence analysis confirmed transgene integration at the programmed cleavage site. Moreover, we confirmed the precise integration of SmUbi-EGFP-SmUbi into GSH1 resulting from HDR by alignment analysis of these sequences, which were obtained from purified 5′-KI and 3′-KI amplicons (Fig. 3D). No 5′-KI and 3′-KI fragments were amplified from gDNA of the control groups. A 1466-bp fragment of the unedited GSH, which was amplified in all experimental groups, served as a PCR control.

**Fig. 3.**
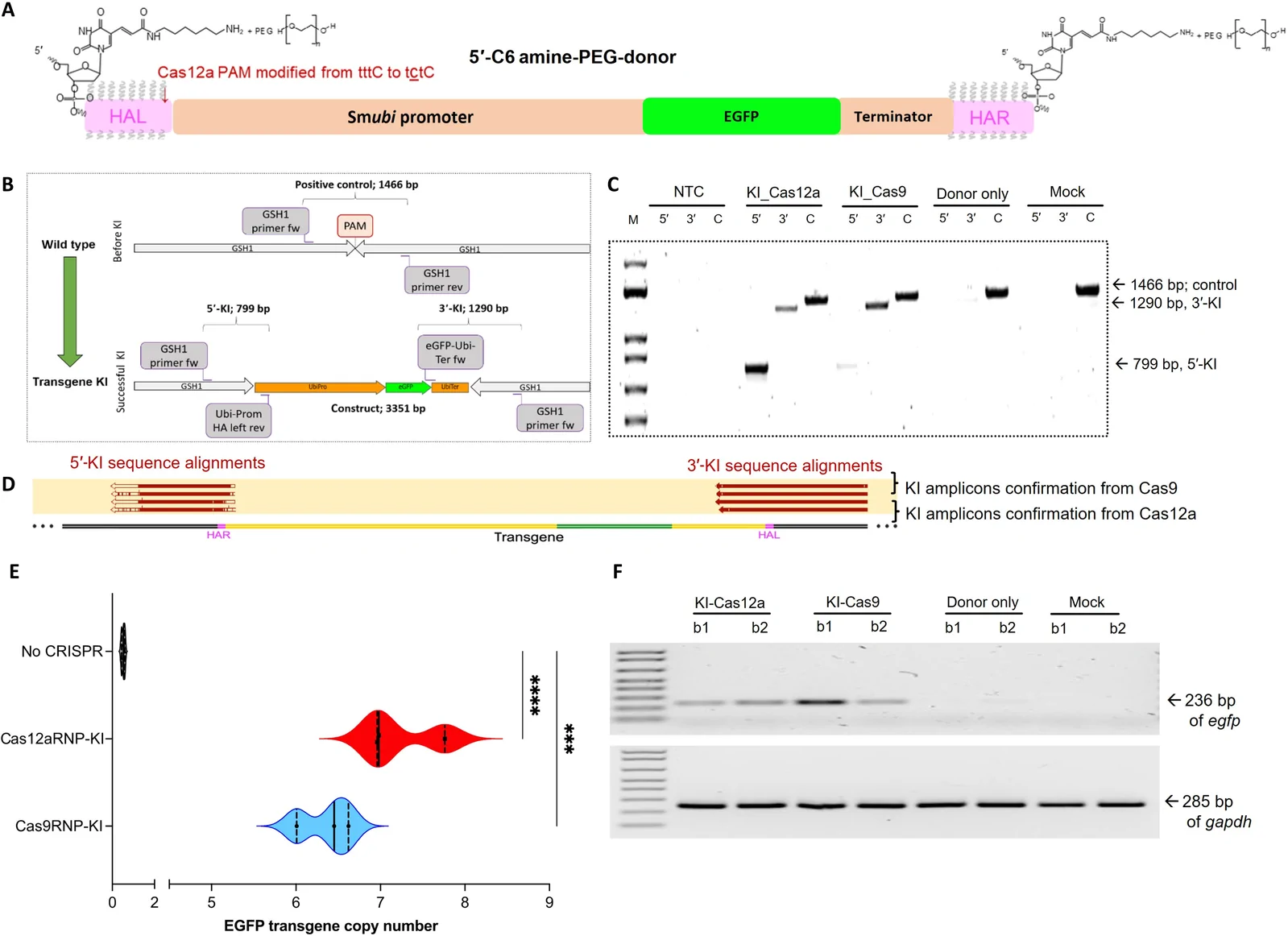
Successful RNP-mediated chromosomal integration of the SmUbi-EGFP-SmUbi reporter gene with HAs into GSH1. (**A**) HDR-mediated integration of the SmUbi-EGFP-SmUbi reporter into GSH1. Shown are the *S. mansoni* ubiquitin (SmUbi) promoter and terminator (orange), flanking the EGFP reporter (green), and the 50-bp HAs (pink). To avoid Cas12a-mediated cleavage of the 5′-C6 amine and PEG(10)-modified (*49*) donor, the Cas12a PAM inside the HAs was changed. (**B**) Illustration of the GSH1 locus before and after KI of the reporter gene (orange and green), the PAM site (red), and primer binding sites for KI analyses. (**C**) Confirmation of chromosomal transgene integration by PCR. The GSH1 WT locus served as a control. (**D**) For both Cas nucleases, alignment analyses of fragments from two biological replicates confirmed the integration of the donor (yellow and green) into GSH1 (gray). (**E**) Estimation of transgene copy numbers in gDNA (10 days post-EPO) based on applying a standard curve plotted from a serial dilution of donor plasmid containing DNA (*14*, *15*). The copy numbers in the Cas-treated groups were significantly higher than LEs only transfected with the donor [two-way analysis of variance (ANOVA), ***P = 0.0001 and ****P < 0.0001]. (**F**) RNA of eggs of all experimental groups were analyzed by RT-PCR to monitor *egfp* transcription. RT-PCR of two biological replicates (b1 and b2) showed *egfp*-specific amplicons (236 bp) in LEs only from KI experiments. We used DNase I–treated RNA for RT-PCR to eliminate potentially contaminating DNA and remaining donor. No *egfp* transcripts occurred in the control groups. Sm*gapdh* (Smp_0569701, 285 bp; bottom) served as a reference for cDNA synthesis and RT-PCRs. Abbreviations: b, biological replicate; ctr, control; gDNA, genomic DNA; HA, homology arms; HAL, homology arm left side; HAR, homology arm right side; KI, knock-in; NTC, nontreated control. Created in BioRender. Möscheid, M. (2026) https://BioRender.com/rggzb0g.

Subsequently, we quantified the copy numbers of chromosomally integrated transgenes in edited LEs compared to LEs, which were transfected only with the donor template in the absence of RNPs (Fig. 3E). We calculated the *egfp* transgene copy number in GSH1 by converting the measured Ct values to the logarithmic copy numbers using the equations obtained from the standard curve. There were 6594 ± 0.196 and 7235 ± 0.458 *egfp* copies in ∼10 ng of DNA resulting from Cas9 and Cas12a, respectively. The control group (donor template only) showed 0.516 ± 0.082 copies (10 ng of gDNA), which likely reflected residual nonintegrated SmUbi-EGFP-SmUbi donor DNA. Accordingly, *egfp* transcripts were not detected by RT-PCR of the RNA/cDNA of the control groups and only found in KI in DNase I–treated RNA samples (Fig. 3F). In summary, the number of integrated SmUbi-EGFP-SmUbi copies was significantly higher using Cas12a compared to Cas9.

### EPO of eggs resulted in EGFP expression in developing miracidia

To monitor transgene expression in developing miracidia obtained from KI treatments, LEs were viewed at days 5 and 10 post-EPO by CLSM (*14*, *15*) (Fig. 4, A and B). Emission spectra were captured and analyzed as previously described (*14*, *15*). EGFP signals occurred in numerous cells inside the eggs including miracidia, which had developed in eggs. Fluorescence intensities (509 nm; representing the EGFP emission peak as illustrated in Fig. 4C) were recorded and quantified in eggs from editing experiments. EGFP-positive eggs of the Cas12a group significantly outnumbered positive eggs of the Cas9 group at both days 5 and 10 (Fig. 4B). For this analysis, only eggs containing miracidia with >50% EGFP expression in the investigated section plane (two dimensions) were counted as EGFP positive. Differences in terms of growth and survival were not noted between successfully transfected eggs and eggs from the control groups. Furthermore, we demonstrated the viability of treated eggs by miracidia hatching. From six biological replicates, the percentages of EGFP-positive eggs were 43.6 ± 19.9% (mean ± SD) and 26.3 ± 7.2% in the Cas12a group at days 5 and 10, respectively, and 20.2 ± 10.1 and 21 ± 16.2% in the Cas9 group at the same time points, respectively (Fig. 4B). Weak or no EGFP signals were detected in LEs transfected with the donor only (no RNP). RT-PCR analyses with egg RNA/cDNA of all groups confirmed *egfp* transcripts in the treatment groups but not in the control, except a weak EGFP background signal in LEs transfected with the donor template in the absence of RNP (Fig. 3C). Furthermore, EGFP intensities in miracidia inside LEs were determined 10 days post-EPO, whereby autofluorescence signals were subtracted. To this end, 100 eggs of each treatment group were analyzed for EGFP signal intensities from miracidia-containing LEs (*14*, *15*). There was no significant difference in the fluorescence intensities of larvae inside the eggs between the Cas9 and Cas12 groups. EGFP signals were not detected in the control groups (donor only, mock; Fig. 4D).

**Fig. 4.**
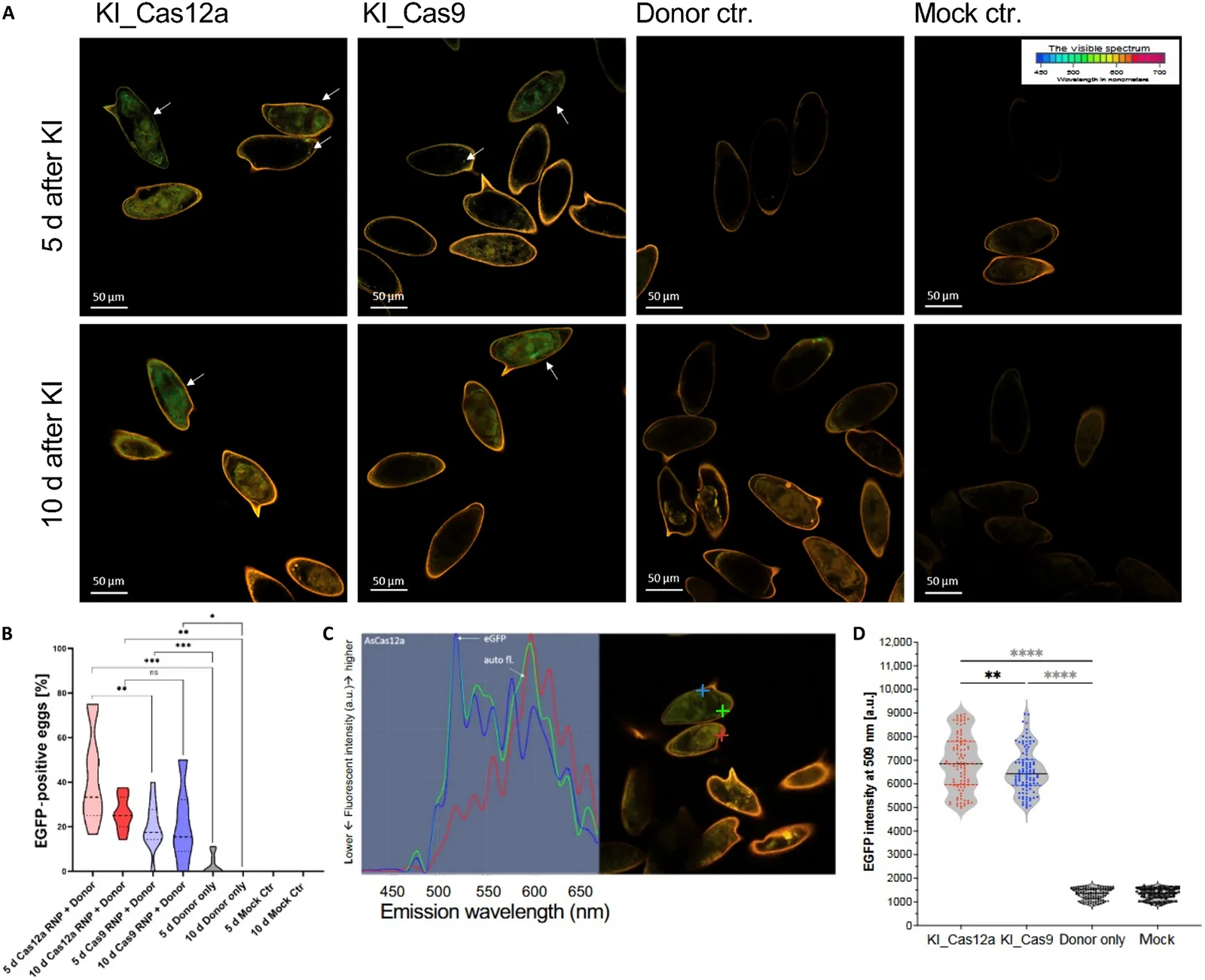
Enhanced KI efficiency of CRISPR-Cas12a compared to Cas9 at GSH1 in *S. mansoni* miracidia. (**A**) Within the eggs, green fluorescent cells of developing and fully developed miracidia were observed by CLSM at 5 and 10 days (d) after treatment with RNPs targeting GSH1, respectively. The orange color resembles an artifactual autofluorescence. Scale bars, 50 μm. (**B**) CLSM analysis showed the proportion of EGFP-positive larvae in eggs at 5 and 10 days after KI experiments. About 10,000 LEs of each biological replicate (*n* = 6) were studied. Comparing Cas9 (blue) and Cas12a (red) displayed higher EGFP-positive rates in LEs after 5 days. Cas-treated eggs contained significantly more fluorescent larvae as the donor-only control (gray). No fluorescence signals occurred in untreated eggs. *P < 0.05, **P < 0.01, and ***P < 0.001 by a *t* test. (**C** and **D**) Verification of EGFP signals in LEs transformed with Cas12a RNPs after 5 days. Different positions of the fluorescence spectrum were sampled to distinguish EGFP from background fluorescence (C). The typical EGFP spectrum (left; blue and green lines) was evident in the larvae (right; green and blue + signs) and was distinct from the autofluorescence spectrum (right; red + sign) of the egg, which mainly occurred in the eggshell (red + sign). (D) The non-HDR miracidium from donor only and mock control showed fluorescence intensity at ∼1333.84 ± 235.39 arbitrary units (a.u.) (mean + SD) and 1331.12 ± 1169.31 a.u., respectively. EGFP-KI mediated by Cas12a resulted in higher signal intensities (6548.71 ± 941.11) than Cas9 (6897.25 ± 1169.31). The EGFP signals from KI_Cas12a eggs were statistically significantly higher than those of KI_Cas9 eggs following ordinary one-way ANOVA analysis, **P = 0.0079 and ****P < 0.0001 (*n* = 100). The spectral intensities of autofluorescence in the controls, donor only (no CRISPR materials), and mock electroporated eggs were not statistically significant. Abbreviation: ctr, control: KI, knock-in. Created in BioRender. Möscheid, M. (2026) https://BioRender.com/p12w4z9.

### Transgenic schistosomes were generated using CRISPR-Cas12a

On the basis of these findings, we attempted to introduce transgenic miracidia into the life cycle to establish a transgenic *S. mansoni* line, a goal not yet achieved in the field of schistosomiasis research. We took two approaches: a “biological approach” using transgenic miracidia for snail infection and a “surgical approach,” where sporocysts were transplanted into snails (Fig. 5A). For the former, 40 snails were each exposed to 10 to 15 miracidia derived 48 hours after EPO-mediated transformation of LEs, which had been prescreened by fluorescence microscopy. Control snails were infected either with untreated (WT) miracidia or with miracidia hatching from LEs electroporated with the donor template only. About 5% of the snails of the KI approach released cercariae, which exhibited programmed editing of GSH1 (Table 1). Perhaps due to postinfection stress, some snails died during the subsequent culture period, reducing the number of snails that produced cercariae. As a result, schistosome lines with successful transgene integration were likely lost. Nevertheless, we proceeded using sibling-derived cercariae for the final host infection. CLSM analysis of the worms recovered from hamsters infected with the putatively transgenic cercariae failed to reveal EGFP expression (fig. S5A). However, the application of a T7E1 mismatch detection assay, which uncovers DNA strands with CRISPR-Cas–induced mutations and unedited strand (*48*), in combination with Sanger sequencing of GSH1, identified a deletion of 9 nt at the predicted Cas12a cleavage site, which was absent in WT worms (fig. S5, B and C). This finding indicated that, despite the absence of genomic integration into the genome, Cas12a-mediated GSH1 indels can be stably transmitted through the life cycle.

**Fig. 5.**
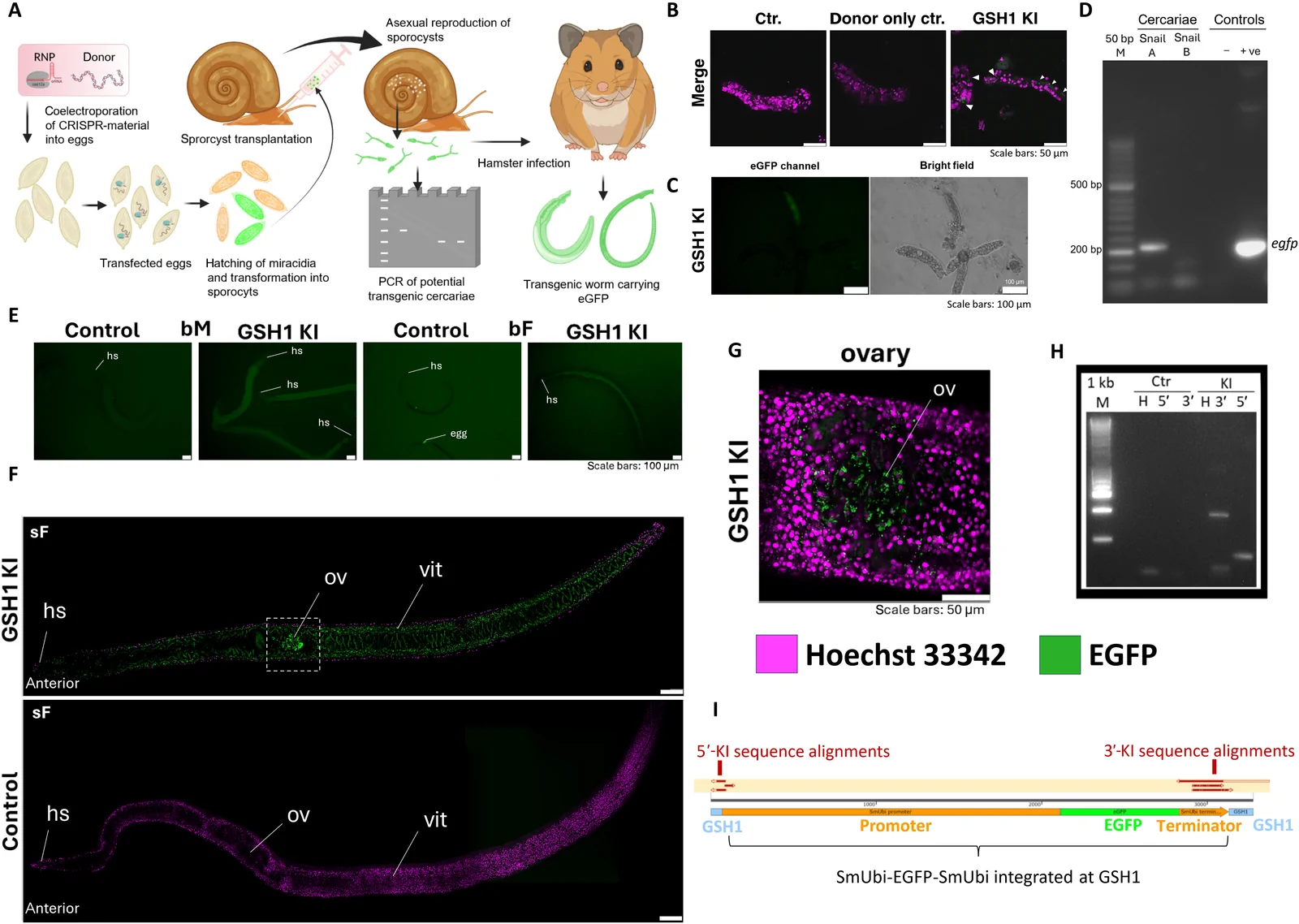
CRSIPR/Cas12a-mediated germline integration of the SmUbi-EGFP-SmUbi transgene. (**A**) Workflow of the surgical transplantation approach of transgenic sporocysts into *B. glabrata*. (**B**) Maximum projection of 20 overlaid *Z*-stacks of 4 days old sporocysts derived from miracidia of KI treatments using Cas12a RNPs. No EGFP signal was detected in control sporocysts; EGFP-positive signals were observed in the KI group (arrows). Sporocysts were counterstained with Hoechst 33342 (purple). (**C**) Representative fluorescence micrograph to highlight *egfp*-positive sporocysts. (**D**) An EGFP-specific fragment of 236 bp was amplified from cercariae harvested from Snail A. Plasmid DNA harboring the SmUbi-EGFP-SmUbi construct served as the positive control. (**E** and **F**) Adult *S. mansoni* were obtained from hamsters infected with cercariae from either monomiracidial snail infection or sporocyst transplantation generated from potentially transgenic miracidia. Adult WT worms served as controls. (E) Fluorescence microscopy of living males (bM) and females (bF) revealed green fluorescence signals in worms from KI experiments only, not in control WT worms. (F) Transplantation experiments of sporocysts, selected on the basis of EGFP-positive signals, (after cercariae recovery and hamster infection) led to the recovery of a female-only population. CLSM analysis exhibited EGFP signals in nearly all tissues of females obtained. A representative female is shown (top). EGFP-positive cells were not found in control females (bottom). (**G**) The ovaries of transgenic females showed EGFP-specific fluorescence. (**H**) We confirmed transgene integration by PCR and sequence analysis of amplicons spanning the 3′- and 5′-integration sites. (**I**) Sanger direct sequencing of both ends of the transgene in GSH1 confirmed transgene integration in worms obtained from three different hamsters infected by cercariae from the same snail. Abbreviations: bF, pairing-experienced females; bM, pairing-experienced males; Ctr, control; hs, head sucker, imO, immature ovary; mO, mature ovary; ov, ovary; vit, vitellarium; KI, knock-in; −, EGFP-negative control; +ve, EGFP-positive control. Created in BioRender. Möscheid, M. (2026) https://BioRender.com/zmq6far.

**Table 1.** Evaluation of the efficacy of several methods for transmitting the transgene through the schistosome life cycle.

Method	Sporocyst transplantation	Monomiracidial infection	Polymiracidial infection
Snails infected	40	185	40
Snails shedding cercariae	5% (2/40)	6.7% (12/185)	7.5% (3/40)
GSH1 editing evidence	2.5% (1/40)	2.2% (4/185)	5% (2/40)
EGFP-positive worms	Yes	Yes	No

Not all potentially transgenic (green fluorescent) miracidia may be infectious, and not all miracidia will successfully transform into mother sporocysts. Furthermore, EPO may influence locomotion and directional swimming behavior, probably due to potential effects on the neuronal system of miracidia (*50*). This could affect their infection competence. To circumvent these potential hurdles, we also tested surgical transplantation (*51*–*53*) of in vitro–generated sporocysts as an alternative approach to reintroduce transgenic larvae into the life cycle (Fig. 5A). To ensure vitality and survival in vitro, we optimized the culture conditions for sporocysts, which finally led to continuous growth and an extended life span for more than 28 days in vitro (fig. S6 A to C). For the editing approach, we electroporated 5000 LEs with Cas12a RNPs and SmUbi-EGFP-SmUbi donor DNA (*n* = 3). EPO of LEs with donor DNA only and eggs electroporated without RNPs and/or donor DNA served as controls. Eggs of all experimental groups were cultured for 5 days at 37°C and 5% CO_2_. Miracidia hatched from these eggs and transformed in vitro into sporocysts, which were cultivated for 4 to 7 days (*54*, *55*). For EGFP expression analysis, sporocysts were examined by CLSM or fluorescence microscopy. EGFP-positive signals were detected only in sporocysts from the Cas12a-mediated KI experiments (Fig. 5, B and C). Most potentially transgenic sporocysts showed mosaic patterns of fluorescent cells (Fig. 5B and fig. S7).

By using an inverse fluorescence microscope, we manually enriched EGFP-positive sporocysts for surgical transplantation into snails, following published protocols (*52*, *53*, *56*). Forty snails were infected with one to five sporocysts each, whereas 20 snails each were infected with control sporocysts. Snails were maintained in aquaria for 8 weeks, by which time ∼5% shed cercariae (Table 1). Unexpectedly, no cercariae were recovered from snails infected with sporocysts from the control group. This could be due to a relatively low number of snails used for the control population and/or due to an increased lethality of this group of snails of unknown origin following surgical transplantation. To confirm reporter gene presence in cercariae of the transgenesis group, 50 individual cercariae were collected from each snail for gDNA isolation and PCR. EGFP-specific amplicons were found in cercariae released by one snail (Snail A; Fig. 5D). Sibling cercariae obtained from the same snail were used to infect the final host. In contrast to the “biological” infection (Fig. 5E), a female-only population was recovered from hamsters infected with cercariae of this surgical transplantation experiment (Fig. 5F). All females of this group were EGFP positive (322 worms recovered from three hamsters), and CLSM analysis showed the distribution of fluorescence in these females (Fig. 5, F and G). EGFP signals were observed along the length of worms including the ovary (Fig. 5, F and G, and fig. S8, A and B), indicating germline transmission of the transgene. We confirmed reporter gene occurrence in GSH1 by PCR and nucleotide sequencing (Fig. 5, H and I, and table S5), which revealed variation in the precision of the transgene integration at the 5′-integration site of GSH1. Analysis of the alignment coverage of the resulting amplicons revealed an average alignment rate of 73.4+/−26% at the 5′-KI and 94.6+/−5.2% at the predicted 3′-KI side, highlighting the variations in the KI precision. However, this did not appear to influence EGFP expression.

In addition, we transformed LEs, as above. Miracidia enriched from eggs showing EGFP signals were used for monomiracidial infections of 185 snails. Of these snails, 6.7% shed cercariae by 10 weeks later. About one-third of the cercariae shed from these snails carried the reporter gene and siblings of these cercarial populations were used for final host infection. Fluorescence microscopy revealed EGFP expression in female and male worms that developed in hamsters infected with these cercariae. In contrast to control worms, signals were only detected in worms from KI experiments. The distribution of EGFP varied among the worms but included signals from the ovary, again indicating transgene transmission via the germ line (Fig. 5E and fig. S8B).

Last, to investigate transgene inheritance, we performed also infections of hamsters with both EGFP PCR-positive cercariae obtained from snails infected with single transgenic miracidia (monomiracidial infection) and WT cercariae, aiming to provide a sexually reproducing population of adult F0 generation schistosomes. EGFP-positive F0 adult worms of both sexes were recovered from the hamsters. Moreover, after egg recovery of F0 worms and eggs from hamsters, miracidia hatching, and snail infection, clonal cercariae of this F1 generation were mixed with (unedited) WT cercariae for a second round of hamster infections. CLSM analyses of the F1 generation worms obtained from these hamsters revealed EGFP signals at comparable levels, with 16.6 ± 10.7% of males and 13.7 ± 8.6% of females showing EGFP signals (fig. S9). We saw EGFP also in the ovaries of these females, which convincingly substantiated the stable genome integration and germline transmission of the of the SmUbi-EGFP-SmUbi transgene.

## DISCUSSION

Here, we took on the challenge to advance functional genomics of a typical—albeit one that is also a major neglected tropical disease pathogen—parasitic platyhelminth (*57*, *58*), *S. mansoni*, and now report progress in genome editing at the previously identified GSH site GSH1 (*14*). To this end, we first compared the editing capacities of Cas9 and Cas12a, optimized RNP delivery conditions and donor DNA template structure, tested different approaches of life cycle reintroduction of edited parasites stages, optimized sporocyst culture as a prerequisite for the surgical transplantation, and lastly achieved efficient and stable germline integration and filial generation transmission of a reporter gene into GSH1. As an additional technical modification, we used 5′-C6-PEG10–modified 50-bp HAs to program CRISPR-Cas9/Cas12a–induced HDR repair of GSH1.

Knockout models are widely used for functional studies of gene(s) of interest. However, in the omics era of schistosomes and other helminth research, facile protocols for stable transformation are not readily available. A first breakthrough in this context was the successful generation of genetically modified *B. glabrata*, the intermediate sail host of *S. mansoni*, using CRISPR-Cas9–based editing (*3*). CRISPR-Cas gene editing is now the state of the art for genome manipulation (*59*), and there are reports involving Cas-mediated gene editing approaches in trematodes (*14*, *16*, *43*, *58*, *60*). Thus far, genome editing was limited to eggs, and genome manipulation probably accessed the outer cell layers during embryogenesis (*17*, *61*, *62*). Yet, to establish stably transformed lines, approaches need optimized germline transgenesis. We had established a protocol for RNA-programmed Cas9-catalyzed editing at GSH1 in *S. mansoni* that used multiple, overlapping gRNAs, after computational prediction of the genome coordinates for this GSH based on genome annotation, chromosome structure, and experimental validation (*14*, *15*). Here, we boosted both this method and enhanced gene editing efficiency using a single gRNA-programmed Cas12a in the presence of a donor repair template bearing chemically modified HAs, which, in combination, enhanced programmed KI of the transgene.

In previous studies, both egg and adult stages of *S. mansoni* were transfected by EPO using Cas-containing RNPs. Treatment of adult schistosomes this way failed to achieve a penetration and distribution RNPs in all tissues (*43*). Exposure of adult-stage schistosomes to CRISPR-Cas by EPO can be expected to result in genetic mosaicism. Our findings conformed with earlier reports of deletion mutations at CRISPR-Cas targets in LEs, schistosomula, and adult schistosomes (*15*, *16*, *58*, *59*, *63*, *64*). Nonetheless, the outcome has been informative as proof of principle for genome editing or as an alternative for RNA interference (RNAi) of genes transcribed in the (sub)tegument.

To overcome these impediments and to establish a genetically homogeneously modified multicellular parasite, it is necessary to target life stages with a limited number of cells, or a stage rich in stem/germinal cells such as the schistosomulum (*65*), or at an early developmental stage such as the zygote (*17*). For this purpose, we aimed to introduce RNPs into LEs isolated from livers of infected rodents, which consisted of egg subpopulations representing a spectrum of developmental stages. Notably, this LE population includes nonembryonated eggs, consisting of the zygote and a few vitelline cells (*17*). Confocal microscopy of LEs transfected with RNPs, which incorporated rhodamine-labeled Cas9-gRNAs (*43*) or Cas12a-gRNAs, demonstrated the suitability of this approach to permeate the eggshell. Here, Cas12a RNPs showed a higher efficiency to access the schistosome embryo within the eggshell. Moreover, transfection of the zygotes at an early developmental stage was visualized by RNP tracking experiments (fig. S10).

A limitation of our approach was that RNPs of both nucleases were not detected in all cells of the eggs. To optimize this, alternative transfection methods such as lipid nanoparticles (*66*) and other methods (*66*–*71*) could be tested in the future. Alternatively, other larval stages such as the sporocysts could be targeted, aiming to enhance transfection efficiency (*43*).

Analysis of the deletion frequencies of GSH1 revealed a significantly higher editing efficiency of Cas12a over Cas9 in eggs and adult schistosomes. The editing efficiency of Cas12a with a single gRNA was comparable to the efficiency of Cas9 with two to three gRNAs (*72*). This difference might result from (i) the efficiency bias of Cas12a for penetration of the eggshell, (ii) differences in the cleavage profiles of the nucleases (Cas9, blunt ends; Cas12a, overhanging ends) (*9*), (iii) divergent efficiency of NHEJ, which relies on the gRNA sequence and the cell cycle stage of the cells (*73*–*75*), and/or (iv) combinations of these factors. Intriguingly, the deletion patterns of Cas12a and Cas9 differed between the egg and the adult worm, suggesting that DNA repair mechanisms might differ during schistosome development. The egg in utero is comprised mostly of S4 vitelline cells (*76*), the fertilized oocyte in the early phase, and, at a later stage, the miracidium with surrounding acellular and cellular layers: Reynolds’ layer and von Lichtenberg’s envelope, respectively (*17*, *77*, *78*). We speculate that DNA repair profiles differ among these cell types and developmental stages. Findings in other metazoan species support the conjecture (*79*–*82*). Aiming to buttress support for this hypothesis, we performed an in silico approach to identify orthologs in *S. mansoni* of genes participating in dsDNA break repair, NHEJ and HDR, in *S. mansoni* based on data available for the model nematodes, *Caenorhabditis elegans* and *Pristionchus pacificus* (fig. S11 and table S6) (*83*–*88*). Notably, schistosome proteins predicted to participate DSB repair did not exhibit marked identity to mammalian DSB repair pathway members (*75*, *89*). Expression patterns of *S. mansoni* protein-coding genes, predicted to be involved in DSB repair (table S6) (*20*, *36*, *90*), showed clear differences from the tissue-preferential transcript profiles of genes attributable to NHEJ (e.g., SmCKU70 and SmCKU80) or HDR (e.g., SmHIM6, SmRAD51, and SmRPA). NHEJ-associated genes were transcribed throughout the schistosome tissues, whereas HDR-associated proteins were preferentially transcribed in the gonads (*20*, *82*, *91*). Genes predicted to be involved in HDR, including Sm*rad51*, Sm*rpa*, and Sm*brc1*, were not transcribed in S4 vitellocytes (*90*). These variations might underline divergent mutation rates and alleles between the egg and adult stages. In addition, HDR is known to be the prominent repair pathway operating in proliferating cells including embryonic cells (*75*) and where indels are not expected to accompany perfect DSB repair (*81*). Correspondingly, increased transcript levels of HDR-associated genes might be anticipated in the parasitic stages of the developmental cycle including the sporocyst, which undergo intense cellular proliferation (*36*, *92*). By contrast, we hypothesize that terminally differentiated cells such as the S4 vitelline cell likely rely on NHEJ (*75*, *89*) for DNA repair (*73*, *80*).

Furthermore, we confirmed the utility of microhomology (50 nt) arms for CRISPR-Cas–induced HDR repair, as demonstrated by constitutive reporter gene expression in the embryonating miracidium. Cas9 and Cas12a were each introduced into LEs the presence of a 5′-C6-PEG10–modified donor template coding for EGFP under control of the endogenous ubiquitin promoter of *S. mansoni* (*14*, *15*). We analyzed this promoter in detail and identified several core promoter elements (*33*, *93*–*96*), such as a potential minimal promoter sequence (table S7) including a schistosomal Kozak sequence (*34*). The promoter elements may contribute to the constitutive transcriptional activity of the cognate ubiquitin gene in the developmental cycle (*36*). Constraints on transgene cargo size delivered by HDR are well known (*14*, *97*, *98*). However, the use of a minimal length (HAs) of 50 bp in length flanking the transgene, i.e., HAs as deployed here, can facilitate insertion of transgene cargo up to at least 9 kb in length (*49*, *99*–*101*).

For reporter gene integration, we used a chemically modified donor DNA template, modifications aimed at preventing DNA degeneration by endogenous nucleases and known to enhance HDR in human cell lines (HCT116, HEK293T, H1, and WTC G3) (*49*, *102*). Superior KI performance has been reported for the donor template in human cells when the template combined 5′-C6-PEG10 modification and short HAs (*49*, *103*, *104*). Transgene insertion into GSH1 using this adapted approach for schistosomes represents a significant optimization over our original protocol (*15*) as both HAs were substantially shorter, and dsDNA could be used instead of a single-stranded DNA donor. Likewise, with an RNP assembled with Cas12a and a single crRNA, we achieved comparable and higher integration rates with the modified donor template compared with Cas9 and up to three gRNAs (*14*). Notably, Cas12a appeared to be well suited for *S. mansoni*, given its AT-rich genome (*105*), and given that Cas12a has a T-rich specific PAM, 5′-TTTN-3′ (*9*), whereby the used crRNA showed substantial lower numbers of predicted off-targets (table S8). In overview, Cas12a exhibits a divergent flexibility to Cas9, which is constrained in its target sequence range in the schistosome genome due to its CG-rich PAM, 5′-NGG-3′. Furthermore, the use of Cas12a requires a single gRNA (crRNA), which reduces the risk of potential off-target effects and expenses. Reintroducing potential transgenic miracidia into the life cycle succeeded by infecting snails with these cercariae directly. However, to improve upon the success rates of natural snail infection by miracidia, especially when EPO influences locomotion and directional swimming and thus may affect their infection capacity, we also used an alternative approach. To this end, miracidia from edited LEs were transformed in vitro into sporocysts (*54*, *55*), maintained under conditions optimized in our study. The number of EGFP-positive sporocysts was comparable to the number of EGFP-positive eggs after 10 days in vitro. Sporocysts showed a mosaic of EGFP-positive cells, with sporocysts containing EGFP-positive cells in the center (Fig. 5B). On the basis of reports assessing sporocyst morphology, this structure should harbor germ cells (*50*, *106*), which either directly enter cercariogenesis or develop into another daughter sporocyst generation by asexual reproduction (*78*, *106*, *107*). For reintegration into the life cycle, in vitro–generated, EGFP-positive sporocysts were surgically transplanted into snails (*52*, *53*, *56*). Although some snails died posttransplantation, this manipulation did not arrest the biological activity of the transplanted larvae because cercariae were eventually released from the surviving snails. PCR confirmed reporter gene presence. Upon final host infection, EGFP-positive and thus transgenic female worms were obtained. Although the general efficiency of this method was only ∼2%, this improves upon existing protocols. On the level of editing eggs and miracidia hatching, efficiencies varied among biological replicates but reached about 40% at best in our hands. Not only this value but also lower efficiencies provide a sound basis for successful snail infection and the recovery of transgenic cercariae. However, developmental processes in the snail may eventually represent a bottleneck. Compared to the WT control, cercariogenesis appeared occasionally delayed. Furthermore, some snails died before cercariae shedding, and not all infected snails surviving longer periods postinfection produced cercariae.

The resultant population of transgenic females was probably derived from one single female sporocyst, which survived and reproduced asexually to generate clonal cercariae (*78*, *108*), and continued intra-snail development or was perhaps derived from several female sporocysts (*109*). CLSM analysis of these female schistosomes revealed EGFP expression in various tissues. This included the ovary, which confirmed successful germline transgenesis. Furthermore, this result reinforced the predicted constitutive activity of the SmUbi promoter (*19*, *36*, *90*). For additional confirmation, we performed PCR and Sanger sequencing showing the integration of the reporter gene into the GSH1 locus. Unexpectedly, Sanger sequencing revealed a discrepancy at the 5′-integration site, which exhibited an unexpected large Cas12a-induced deletion (*14*, *110*) of the GSH1 locus followed by imperfect transgene integration (*111*–*114*). Given the presence of nearly repetitive, AT-rich sequences within the GSH1 locus (*14*, *15*, *105*, *115*) and the tendency for HDR-mediated transgene integration to result in imperfect integrations (*111*–*114*), it may be possible that the transgene was integrated by partial homology (*116*) or error-prone NHEJ-mediated transgene integration (*117*, *118*). T7E1 mismatch detection assays confirmed only small deletions at the predicted Cas12a cleavage site, and GSH1 sequencing postediting showed small variations mainly at the 5′-integration site. Although neither result indicates transgene expression failure, we cannot exclude that the observed EGFP mosaicism could be the result of imperfect transgene integration in some target cells (and their progeny) of the embryo following egg EPO. Alternatively, it may be possible that the regulation of genes in proximity of the GSH1 locus varies during schistosome development, the pairing status, and/or the tissue context, which could influence transgene expression at this locus in trans.

In addition, fluorescence patterns suggested mosaicism in the F0 founder generation of transgenic animals, a common occurrence attributable to the frequent integration of foreign DNA into the genome. Following the initial cell division, a subset of cells will have acquired the transgene, resulting in a heterogeneous population (i.e., a mosaic) comprising both modified and unmodified cells. This phenomenon occurs within the same animal and its germ line, leading to inconsistent inheritance patterns (*119*–*121*). Consequently, establishing a homozygous transgenic line requires crossing F0 with WT worms to confirm germline transmission (as shown in this study) and to generate heterozygous F1 individuals. F1 individuals should subsequently be inbred to produce homozygous F2 in accordance with Mendelian ratios. For subsequent functional experiments, such as specific gene knockouts, breeding should extend to at least the F2 generation to obtain genetically homogeneous animals. An unrelated study investigating the karyotype of germ cells in the closely related trematode *Fasciola hepatica* demonstrated the occurrence of triploids in ∼20% of the flukes (*122*). This observation suggests that filial generations must be carefully evaluated to ensure the robust establishment of homozygous trematode lines. Moreover, transgene insertion in the genome can negatively affect the host cell function or the cell may silence the transgene due to changes in the chromatin structure (*11*, *12*, *123*). Nonetheless, these results represent the proof that editing GSH1 at the egg stage is possible as a prerequisite to reintroduced transgenic life stages, here miracidium and sporocysts, by natural (miracidium–snail infection) or by surgical transplantation (in vitro–generated sporocyst into recipient snail) into the life cycle to obtain transgenic adult *S. mansoni* that are able to vertically transmit the transgene to the following generations.

Generation of EGFP-positive worms was also observed following monomiracidial infections. Here, hatching of LEs, which were cultured in vitro for 2 days, resulted in a notably increased hatching rate, indicating that the duration of in vitro culture also plays a critical role in the survival and development of miracidia. Monomiracidial infection was comparable in efficiency to sporocyst surgical transplantation for obtaining potential transgenic worms. However, the CRISPR-Cas–based method of editing still must be optimized and remains challenging. Further efforts are needed to optimize the in vitro culture to make this method a standard procedure (*54*, *124*–*126*). In this context, optimizing the in vitro culture is a worthwhile goal because it has high potential for improving the production and selection of transgenic larvae. Here, we showed optimized culture conditions for sporocysts, which improves the prerequisite to transplant vital sporocysts into recipient snails. Although, during recent years, some key components, such as human low-density lipoprotein (LDL) and vitamin C, were reported to enhance the maintenance of adult *S. mansoni* and their egg production in vitro, further research is required to determine optimal media compositions (*124*–*126*). Furthermore, the efficiency of germline transmission could be improved by using sporocysts as biological targets for manipulation. Sporocysts are well suited as they can be maintained in vitro for at least 4 weeks (fig. S6A), they have a favorable germ cell/somatic cell ratio (*78*, *92*, *127*), and a transgene can be inherited and propagated by asexual reproduction. An independent study also demonstrated the successful delivery of RNPs in diverse sporocyst cells (*43*). Also, because sporocysts are not autofluorescent, EGFP-positive individuals can readily be screened and selected for surgical transplantation into snails (*52*, *53*). Furthermore, we optimized the EPO protocol and found that a 125-V pulse for 20 ms achieved an optimal balance between miracidia hatching and transformation rates (*14*). In addition, multiple pulses might be advantageous for delivering CRISPR-Cas materials into the eggs. Another alternative for the delivery of CRISPR-Cas materials in the zygote of eggs would be the delivery of CRISPR-Cas mRNA as it has been described in transgenesis protocols for several model organisms (*128*–*130*) or the usage of the recently established vitellogenin (Vtg)–mediated Cas9 RNP delivery in oocytes of the sea star *Patiria miniata* (*131*). For this purpose, the process of endocytosis of Vtg, which is exclusively taken up by oocytes of metazoans, was used (*131*, *132*). Such an approach might be tested for *S. mansoni* eggs to improve editing efficiencies.

Our results underscore the appropriateness of the GSH1 locus for transgene integration (*108*, *133*) and the maintenance of insertion locus integrity throughout the schistosome life cycle. The demonstration of stable, heritable transgenesis in schistosomes overcomes a long-standing technical obstacle in platyhelminth molecular genetics and opens multiple avenues for basic and translational research. The new approach will advance in understanding key questions in subsequent studies such as knockout effects of genes of interest of schistosomes and further (platy)helminths, for which similar techniques do not exist. Furthermore, KI studies are conceivable to, e.g., express transgenes for (i) recombinant protein production (within the native host and its, in part, unique glycosylation patterns (*134*)] or (ii), in a gain-of-function manner, for medical purposes using parasitic worms as vehicles for immunization and other therapeutic purposes (*135*, *136*). A new proof-of-concept study following a similar technical approach for genome editing demonstrated the successful integration, expression, and secretion of a human single-chain variable fragment antibody from a GSH of the hookworm *Ancylostoma ceylanicum* (*137*). In case selectable marker genes are integrated into GSH1 (or a target gene of interest locus) as an alternative for, or in addition to, fluorescence reporters, this would enable the maintenance of transgenic lines under drug selection pressure. Oxamniquine showed selectivity for sporocysts and adult schistosomes (*43*, *138*). Beyond that, this model could be used for testing target genes/proteins of schistosomes or other helminth parasites for their suitability as potential targets with the goal of developing new drugs and vaccines to treat and prevent schistosomiasis and other similar diseases. This method also opens the way for studying genes inaccessible to RNAi (*72*, *139*, *140*). We respectfully posit that this new workflow can provide a blueprint for the design of more advanced, tissue-stage investigation and can contribute to deciphering the complex biology of schistosomes and closely related trematodes.

## MATERIALS AND METHODS

### Maintenance and isolation of *S. mansoni* developmental stages

The following procedures were previously described in (*141*). Mice (female, Swiss Webster) exposed to about 180 cercariae of *S. mansoni* were provided by the schistosomiasis Resource Center, Biomedical Research Institute (Rockville, MD). The mice were housed at the Animal Research Facility of George Washington University (GWU), which is accredited by the American Association for Accreditation of Laboratory Animal Care (AAALAC no. 000347) and has the Animal Welfare Assurance on file with the National Institutes of Health, Office of Laboratory Animal Welfare (OLAW assurance no. A3205). All procedures used were consistent with the Guide for the Care and Use of Laboratory Animals. The Institutional Animal Care and Use Committee at GWU approved the protocol used for mice in this study. Mice were euthanized at about day 49 following infection. Schistosomes were recovered by hepatic portal vein perfusion with 150 mM sodium chloride and 15 mM sodium citrate (pH 7). The worms were washed with 1x phosphate-buffered saline (PBS) (pH 7.4) and 2% penicillin/streptomycin/fungizone (PSF) and maintained in Dulbecco’s modified Eagle’s medium (DMEM) and 10% heat-inactivated fetal bovine serum (FBS) (Corning Life Sciences, Corning, NY) supplemented with 2% antibiotic/antimycotic (Corning) in the atmosphere of 5% CO_2_ and a 37°C incubator (Panasonic, Newark, NJ) (*14*).

Experiments conducted on hamsters at Justus Liebig University Giessen (JLU) complied with the European Convention for the Protection of Vertebrate Animals used for Experimental and other Scientific Purposes (ETS no. 123; revised Appendix A) and received approval from the Regional Council (Regierungspraesidium) Giessen (V54-19c 20 15h 02 GI18/10).

Adult schistosomes were processed within 24 hours following recovery from mice. To prepare *S. mansoni* eggs, mouse (GWU) and/or hamster (JLU) livers were rinsed in 70% ethanol, washed twice with 1x PBS and 2% penicillin/streptomycin solution, and lastly homogenized in 1x PBS and 2% PSF in a mechanical blender. The homogenate was digested with clostridial collagenase (0.7 mg/ml) at 37°C for 18 hours, after which the cellular debris was removed using sieves of 250- and 125-μm mesh (Gilson, Middleton, WI). Sequentially, followed by sucrose/Percoll gradient centrifugation, eggs were recovered (*142*). LEs were maintained at 37°C in 20% FBS/2% PSF/DMEM and electroporated with CRISPR-Cas materials with or without donor (below) within 24 hours.

For isolating unembryonated eggs, in vitro–laid eggs (IVLEs) at 0 to 24 hours (*17*) were collected. For this, the media of couples 24 hours postperfusion were collected and rinsed first through a 180-μm cell and second through a 15-μm cell strainer. IVLEs inside the cell strainer were washed twice with sterile PBS supplemented with 1% ABAM. Subsequently, IVLEs were resuspended in 10 ml of M199+++ supplemented with chloramphenicol (50 μg/ml) and transferred to 50-ml tubes. This step was followed by an incubation for 1 hour at 37°C. Then, IVLEs were centrifuged for 10 min at 600*g* at room temperature (RT), and the supernatant was discarded. These eggs were transferred into 2 ml of M199+++ supplemented with 20% NCS and incubated at 37°C and 5% CO_2_.

*B. glabrata* snails were maintained in snail water at 26°C, with a regime of 16 hours of light and 8 hours of darkness. For polymiracidial infections, snails were placed individually in 12-well microtiter plates containing 2 ml of snail water and 10 to 15 miracidia for 16 hours before they returned to the incubator. For monomiracidial infections, snails were wiped dry by a paper towel. A single miracidium was pipetted to a drop of 10 μl of snail water and transferred to a well of a 12-well tissue-culture plate. The shell aperture of the previously wiped snail was then placed on the miracidium-containing drop. Then, the snail was incubated on a light plate for 15 min, after which 100 μl of sterile tab water was added directly to the snail to prevent desiccation. After 60 min, 2 ml of snail water was added. Infection was performed overnight, as described above.

### Cultivation of Bge cells and collection of conditioned media

The following procedures were previously described in (*141*). Embryonic cells of the intermediate snail host *B. glabrata* [*Bge* (CRL-1494, American Type Culture Collection, Manassas, USA] were cultivated according to the Bge cell culture protocol of the Biomedical Research Institute (BRI) established by Ittiprasert and Miller (*143*). *Bge* cell–conditioned medium was used as supplement for the in vitro cultivation of sporocysts to simulate the intermediate snail host. For this, the medium of a *Bge* culture at a confluence of 70% was exchanged with prewarmed (26°C), Hepes-free, StemMACS iPS-Brew XF embryo cell medium (MACS Media, Miltenyi Biotec) supplemented with 5% NCS. The medium was conditioned for 7 days, collected, and sterile filtered. The filtered medium was stored at 4°C for immediate usage or frozen in liquid nitrogen and stored at −80°C.

### In vitro transformation of miracidia into sporocysts

The following procedures were previously described in (*141*). A modified protocol based on the works of Chernin (*54*, *55*), Voge and Seidel (*144*, *145*), and Yoshino and Laursen (*51*) was developed to transform miracidia into sporocysts and for in vitro cultivation. LEs were hatched under sterile conditions, and miracidia were collected and washed with 50 ml of PBS using a 15-μm cell strainer. Subsequently, miracidia were transferred into a reaction vessel with CBSS buffer (*55*) supplemented with trehalose (1 g/liter; CBSS+T, Sigma-Aldrich) and 1% ABAM. Miracidia were incubated for 2 hours at 26°C in reduced oxygen conditions (5% O_2_, 5% CO_2_, and 90% N_2_) and then incubated for 30 min on ice. This immobilized miracidia, which were then counted. Approximately 1000 up to 2500 miracidia were transferred into a well of a 12-well plate, and the remaining CBSS+T was exchanged by 3 ml of Hepes-free sporocyst medium (M199sporo; 48% CBSS+T, 48% M199 without supplements, 5% 7-day Bge cell–conditioned StemMACS iPS-Brew XF medium, and 1% ABAM). Miracidia were cultivated in 5% O_2_, 5% CO_2_, and 90% N_2_ at 26°C under for at least 24 hours, which induced their transformation into sporocysts. Ciliated plates were removed by washing the sporocysts using a 15-μm cell strainer and further incubated as above.

### Vital staining

The following procedures were previously described (*141*). Before surgical transplantation, a representative fraction of cultivated sporocysts was screened to determine cellular integrity. For this, 100 sporocysts were incubated in 500 μl of M199 medium containing 2 μM Hoechst 33342, 5 μM SYTOX Orange, and 2 μM Calcein AM (each from Thermo Fisher Scientific). Sporocysts were incubated for 30 min and washed twice with M199 and then mounted on a slide in ROTI MOUNT FluorCare (Roth). The samples were analyzed by fluorescence microscopy according to the manufacturer’s instructions.

### Transplanting in vitro–generated sporocysts into the intermediate snail host

The following procedures were previously described in (*141*). The surgical transplantation of sporocysts generated in vitro was carried out according to previous protocols (*52*, *53*, *56*). To this end, potentially transgenic (green fluorescent) sporocysts were manually enriched upon monitoring under an inverted fluorescence microscope (DM IL LED, Leica Microsystems, Germany). For transplantation, recipient snails with a shell diameter of ∼1 cm were anaesthetized with 0.08% (v/v) sodium pentobarbital (Nembutal, Sanofi Santé Nutrition Animale) for at least 4 hours at 26°C. During transplantation, the male genital orifice of the snail was manipulated with a microretractor made from glass according to the instructions of Mouahid *et al.* (*56*). An incision was made in the integument of the snail sinus using a cannula. One to five in vitro–cultured sporocysts aged 4 to 7 days after transformation were inoculated using a thin, drawn-out syringe. Older sporocysts were fragile and elongated. They were carefully aspirated with the syringe. The microretractor was removed, after which the snails were transferred to sterile snail water containing 0.02% nitrofurantoin (w/v; Sigma-Aldrich) as an antibiotic for 1 day at 26°C to minimize microbial infection. Thereafter, the snails were maintained in aquaria for at least 8 weeks before cercariae shedding.

### In silico analyses of the ubiquitin regulatory promoter elements

The following procedures were previously described in (*141*). As reporter for transfection, we used EGFP (*146*), which was cloned under the control of the promoter and terminator sequences of Smp_335990 (*21*, *115*), an abundantly transcribed ubiquitin gene of *S. mansoni* (Sm*ubi*) (figs. S1 and S2 and table S1). To this end, the 5′ and 3′ regulatory regions, nucleotide sequences of upstream and downstream UTRs of Smp_335990, were retrieved from WormBase ParaSite (*21*, *115*). To confirm this annotation, we used the protein homology/analogy recognition engine Phyre2 with the protein structure modeling mode (https://www.sbg.bio.ic.ac.uk/phyre2/html/page.cgi?id=index) (*22*). To predict the promoter region of Sm*ubi*, adjacent sequences up to the 3 kb upstream of the start codon were scanned. Promoter elements were identified by the Berkeley *Drosophila* Genome Project Neural Network Promoter Prediction Tool, using a minimum promoter score of 0.8 for eukaryotic promoters (*26*) (https://fruitfly.org/seq_tools/promoter.html and https://github.com/bdgp/bdgp-splice-promoter-tools). In addition, we used the AliBaba2.1 TRANSFAC 4.0 transcription factor binding site prediction tool (https://gene-regulation.com/pub/programs.html) (*28*) to identify potential transcription factor binding sites. We analyzed potential promoter sequences also by AliBaba2.1 TRANSFAC (*26*, *29*), applying the following parameters: The Pairsim function was set to 50, the matrix width was set to 10 bp, a minimum number of overlapping predicted transcription factor binding sites was limited to a total of four for each site with a minimum sequence conversation of 75% coverage, and the factor class was set to 4. In addition, the sequence was analyzed for canonical core promoter elements by the YAPP eukaryotic core promoter predictor using a cutoff score of 0.8. To conduct the promoter analysis, the sequence including the predicted core promoter fused with the sequence of EGFP (717 bp) was used to analyze potential transcription start sites (TSSs). TSS prediction was performed by YAPP (*32*, *33*). To identify the schistosomal Kozak consensus sequence, we performed a comparative analysis applying the AGAT software package to determine also potential Kozak sequences of other genes in the *S. mansoni* genome version 9. To this end, ubiquitously and abundantly transcribed genes were screened for the presence of potential Kozak sequences and patterns like SmUbi (*90*). Specifically, the sequences of Smp_009580, Smp_106930, Smp_042160, Smp_056970, Smp_054160, Smp_182890, Smp_099870, Smp_179300, Smp_111340, Smp_090120, Smp_072330, Smp_003770, Smp_017430, Smp_040130, Smp_155060, and Smp_335990 were compared (*147*). In addition, the 3′UTR of Smubi defined by WormBase ParaSite (*21*, *115*) version 7 of the draft genome of *S. mansoni* (*105*, *148*), was analyzed. To identify the polyadenylation signal, 600 bp downstream from the stop codon were analyzed with the DNAFSMiner-DNA Functional Site Miner polyadenylation side prediction tool (*30*). Subsequently, the RNA secondary structure of the predicted 3′UTR was calculated, including the stop codon and predicted rank 1 (highest) polyadenylation site within the in silico–added polyadenosine tail (10 adenosines). Potential RNA secondary structures characteristic for translation terminator loops were investigated by the RNAfold Webserver Vienna tool, applying the minimum free energy (MFE) model and activated partition function, whereas the model avoids RNA folding with isolated base pairs (http://rna.tbi.univie.ac.at) (*37*, *149*).

### Reporter gene construct

The following procedures were previously described in (*141*). On the basis of the pJC53.2 vector backbone (Addgene, plasmid #26536) (*150*), we cloned the reporter gene construct consisting of EGFP and the promoter and terminator sequences of Sm*ubi* (Fig. 1 and table S1). To this end, 1 μg of pJC53.2 plasmid DNA was cleaved using *Ahd* I [New England Biolabs (NEB), Ipswich, MA] in 50 μl of 1x CutSmart buffer (NEB) at 37°C for 2 hours. The two processed DNA fragments were size separated by agarose gel electrophoresis. The plasmid fragment of 3240 bp was recovered from the gel (Monarch Gel Extraction Kit, NEB, Ipswich, MA).

For cloning, essential DNA fragments, the full-length *egfp* sequence, as well as the putative promoter (5′SmUbi) and terminator (3′SmUbi) regions of the ubiquitin gene, were amplified by PCR (table S5). The used primers were designed according to database information in WormBase ParaSite for Smp_335990 (*21*, *115*). PCR amplicons of each fragment were synthesized each in a 20-μl reaction volume with either 100 ng of genomic DNA (gDNA) or 10 ng of pET-30a_SmDHFR-EGFP (the EGFP-DNA fragment was kindly provided by J. Collins) as a template for the reporter gene EGFP (717 bp). Expected sizes of the specific fragments for 5′Ubi and 3′Ubi were 2056 and 580 bp, respectively. PCR reactions were set up using the Q5 High-Fidelity Polymerase kit (NEB) and the following conditions: 98°C for 3 min, followed by 35 cycles of 95°C for 30 s, 55°C for 30 s, and elongation at 72°C for 75 s. Amplicons were analyzed by agarose gel electrophoresis and recovered as mentioned above. This step was followed by an additional PCR to add HAs to the reporter gene for Gibson assembly (*151*). Primers were designed with HAs of 20 to 26 bp to both the promoter and the terminator sequences of the backbone pJC53.2 flanking the EGFP fragment. Amplification of the promoter sequence was carried out using primers for Gibson assembly (table S5). A two-step PCR protocol was used, in which the template DNA was first denatured at 98°C for 5 min, followed by 10 cycles of 95°C for 30 s, 54°C for 30 s, and 72°C for 75 s, before 25 cycles followed with 62°C for 30 s, and 5 min at 72°C. The entire construct pJC53.2_SmUbi-EGFP-SmUbi was assembled using the NEBuilder Hifi DNA Assembly Cloning Kit (NEB). Competent *Escherichia coli* DH5α (NEB) were transformed with the ligation products and plated on LB agar supplemented with kanamycin and ampicillin (Sigma-Aldrich, Darmstadt, Germany) for 18 hours at 37°C. The presence of the correct insert in plasmids was confirmed by Sanger sequencing (Microsynth SeqLab, Göttingen, Germany).

### Particle bombardment

The following procedures were previously described (*141*). Transfection by PB was used verify gene expression in vivo from the episomal SmUbi-EGFP-SmUbi reporter gene. For PB, we used the PDS 1000/He system (Bio-Rad, Hercules, CA), as previously described (*39*, *71*). In brief, plasmid DNA was isolated using the PureLink Expi Endotoxin-Free Maxi Plasmid Purification Kit (Thermo Fisher Scientific) and precipitated onto gold particles (0.6 μm) using CaCl_2_, spermidine, and ethanol (*39*, *71*) at a final concentration of 5 μg DNA/600 ng gold. Because of the absence of autofluorescence, male schistosomes were transfected. For PB, the culture medium was removed and the worms (10 males per replicate) positioned centrally in the petri dish. The ballistic parameters were as follows: helium at 1550 psi, 3-cm target distance, 15 in Hg (381 mmHg) atmosphere, and 23°C. After PB, prewarmed M199+++ at 37°C was added to the worms, which were maintained in culture for 48 hours before further analysis. Before microscopy, the nuclei of transfected worms were stained with Hoechst 33342 (Invitrogen) for 120 min. Then, the worms were examined using fluorescence microscopy (IX71 Inverted Fluorescence Microscope Pred IX73, Olympus) and CLSM (TCS SP5 VIS confocal laser scanning microscope, Leica). The acquired images were analyzed using the CellSens software (Olympus) and LAS X software (Leica). Worms showing EGFP-induced fluorescence were washed with PBS, transferred to 50 μl of RNAzol RT reagent, and stored in liquid N_2_. Total RNA was isolated from these worms, and its concentration and integrity were evaluated by using an electropherogram using the Agilent 2100 Bioanalyzer (Agilent, Santa Clara, CA). Furthermore, cDNA of each sample was synthesized by reverse transcription of 100 ng of total RNA, as described above. PCRs using these cDNAs were performed in 25-μl reaction volumes with 200 μM dNTPs, 0.5 μM each primer, 1 μl of cDNA, and FIREPol DNA Polymerase (Solis BioDyne). Last, the EGFP transcript of 717 nt was amplified by PCR using the primers EGFP_fw and EGFP_rev (table S5): 98°C for 30 s, 35 cycles of 98°C for 30 s, 55°C for 30 s, and 72°C for 60 s; 10 ng of pJC53.2_ SmUbi-EGFP-SmUbi plasmid DNA served as a positive PCR control.

### RNP complexes

The following procedures were previously described in (*141*). To prepare CRISPR-Cas editing of the AT-rich GSH1 (65.82% AT), target PAMs and adjacent sequences for both Cas9 and Cas12a were predicted using CHOPCHOP (*47**,* *152*), applying the default function against the *S. mansoni* genome. To compare the CRISPR-Cas efficiency between these two programmable endonucleases without cleavage site-specific bias, we identified gRNA sites for Cas9 and Cas12a predicted to exhibit similar CRISPR efficiency (∼50%), the absence of off-targets, and, notably, programmed cleavages at practically identical positions on the targeted region. In addition, the CRISPR RGEN Cas-OFFinder tool (www.rgenome.net/cas-offinder) (*153*) was used to predict genome-wide off-targets of chosen gRNAs. For this, the tool was set up to detect off-targets with up to four mismatches within the target sequence (table S8). Only gRNAs with three or more mismatches at an alternative locus were considered for this study, whereby, for both Cas enzymes, a probability for off-target effect below 0.5% has been previously described (*154*–*159*). Both Cas9 and Cas12a CRISPR-Cas target sites with a probability of less than 0.5% and/or without any predicted off-targets in the *S. mansoni* genome were used in this study. The sgRNAs and crRNA for Cas9 and Cas12a, respectively, were purchased from Integrated DNA Technologies (ALT-system, IDT, Coralville, IA). The customer-defined gRNA sequences for Cas9 and Cas12a were 5′-GAGAUCAAUUAUCUGACAAU-3′ and 5′-GAGAUCAAUUAUCUGACAAUGGAA-3′, respectively. A gRNA with a scrambled and *S. mansoni*–unspecific sequence was used as a control (5′-GCACTACCAGAGCTAACTCA-3′). The Cas9-sgRNA and Cas12a-crRNA were each labeled with CX-rhodamine (Label IT Nucleic Acid Labeling Reagents Rhodamine Kit, Mirus Bio, Madison, WI) (*54*, *61*), after which RNPs of Cas12a were assembled by mixing of 1:1 (w/w, gRNA:enzyme) Alt-R AsCas12a V3 with labeled and purified crRNA and Alt-R SpCas9 Nuclease V3 with labeled, purified sgRNA, as previously described (*16*). Briefly, 10 μg of Cas9 and 10 μg of sgRNA were mixed by pipetting in a final volume of 100 μl of Opti-MEM (Gibco, Thermo Fisher Scientific, Waltham, MA) and incubated for 15 min at 23°C in the dark. Before RNP transfection, ∼10,000 eggs were exposed to 0.25% trypsin in EDTA at 37°C for 5 min, aiming to remove the protein lining the pores of the egg (*160*). The eggs were washed twice in 1x PBS (500 g, RT, 5 min) and resuspended in 100 μl of Opti-MEM in an EPO cuvette (4 mm) (Bio-Rad, Hercules, CA, USA), and 100 μl of labeled RNPs was added. Trypsinized eggs were subjected to 125 V, one pulse for 20 ms with square-wave EPO by the Gene Pulser Xcell Electroporation System (at JLU, Bio-Rad) or the BTX ECM 830 Electro Square Porator (at GWU, Holliston, MA) (*14*–*16*). Subsequently, eggs were cultured in complete medium as described above for 120 min before counterstaining with Hoechst 333420 (H3570, Invitrogen, Waltham, MA, USA) for 30 min. RNP entry into the LE was monitored by fluorescence microscopy. CX-rhodamine–specific signals were localized by CLSM at 576-nm excitation (TCS SP5 VIS confocal laser scanning microscope, Leica), and images were processed using the LAS X software (Leica). Controls included eggs not subjected to EPO, eggs that were electroporated solely in Opti-MEM without CRISPR materials (mock treatment), and eggs transfected with an irrelevant (scramble) gRNA/RNP complex (*14*).

### Donor repair template

The following procedures were previously described in (*141*). The donor DNA template consisting of 3351 bp of the Sm*ubi* promoter (2056 bp), EGFP (717 bp) Sm*ubi* terminator (578 bp), and microhomology arms of 50 bp each was prepared by PCR using 5′-C6-PEG10–modified primers with 50-bp GSH1 (table S5). Yu *et al.* had shown before that DNA donor–containing HAs of 50 bp with 5′ modifications including an amine group, a C6 linker (AmC6), and polyethylene glycol (PEG10) treatment improved the knock-in efficiency of an EGFP reporter gene using CRISPR-Cas9 (*49*). We prepared a DNA donor template by PCR using primers specific to the Sm*ubi* promoter and terminator sequences including 50 nt of HA (table S5) and the 5′ ends, which were PEG10-AmC6 modified (*49*). Briefly, 10 μM 5′-amine C6 primers (IDT, Coralville, IA) were incubated with 1 mM Bis-PEG10-NHS ester (BroadPharm, San Diego, CA) in 1x borate buffer (Thermo Fisher Scientific) at 23°C for 18 hours. Primers were desalted and dissolved in Tris buffer using the Bio-Spin 30 column (Bio-Rad) for PCR. PEG10-5′-AmC6–modified primers were used for the amplification of the donor template using the GoTaq Green Master Mix DNA polymerase (Promega). The SmUbi-EGFP-SmUbi construct with the 600-bp HA sequence (*14*) served as template DNA, with thermocycling of 98°C for 5 min, 6 cycles of 95°C for 20 s, 56°C for 30 s, and 72°C for 3:35 min, which was followed by 25 cycles of 95°C for 20 s, 68°C for 3:35 min, and 72°C for 3:35 min. The presence of an amplicon of 3451 bp in length, the expected size, was confirmed following agarose gel electrophoresis and isolated as described above.

### CRISPR-Cas–based GSH1 editing

The following procedures were previously described in (*141*). Genomic DNA was recovered from LEs, as described before (*14*, *16*). In brief, the eggs were homogenized in DNAzol (Molecular Research Center Inc., Cincinnati, OH) using a motorized pestle (Kimble Chase, Thermo Fisher Scientific or PowerMasher II Nippi, Funakoshi, Tokyo, Japan). Concentration and purity of the isolated DNA were assessed photometrically. For PCR, the region flanking the expected CRISPR-Cas cut site on the target GSH1 locus (∼808 bp) was amplified by GSH1-specific primers (table S5) with thermocycling of 98°C for 3 min, 35 cycles of 95°C for 20 s, 60°C for 30 s, and 72°C for 60 s. Amplicons were size separated in agarose gels (as above), and bands of the expected size were isolated using the NucleoSpin Gel and PCR Clean-up kit (Takara Bio Inc., San Jose, CA) and sequenced by Sanger direct sequencing at Azenta Life Sciences (South Plainfield, NJ) or Microsynth Seqlab (Göttingen, Germany). The CRISPR efficiencies of Cas9 and Cas12a nucleases were estimated by TIDE (*161*) comparing with the control groups (mock and/or WT). To reveal the positions and frequencies of insertion-deletion (indel) around the target GSH1 site, a fragment-harboring target GSH1 CRISPR-Cas cleavage site was amplified by specific primers containing partial Illumina adaptor sequences (table S5). Generated amplicons were suitable for the AmEZ service of Genewiz Azenta Life Science (South Plainfield, NJ) for NGS. PCR was done as follows: 98°C for 3 min, 10 cycles of 95°C for 20 s, 55°C for 30 s, and 72°C for 35 s and 25 cycles of 95°C for 20 s, 62°C for 30 s, and 72°C for 35 s. The profile of indels at and proximal to the programmed cleavage site in GSH1 was revealed using >100,000 NGS paired-end reads using the CRISPREssoV2, RGEN Tool, and Cas-Analyzer (*46*, *48*). This analysis was evaluated using NGS reads as paired-end reads, using the optimal parameters: minimum homology for alignment on amplicon of 60%, base editing target base C and result base T, and quantification window size of 5. The quantification window was set to −3, and the plot window size to 30. The TruSeq2PE adapter-trimming setting was used. With CRISPR RGEN, both sense and antisense reads of the sequence reads were used. The RGEN Tool was also used to investigate the distribution of CRISPR-Cas–mediated indels at GSH1; for this, the nuclease detection mode was set to single nuclease. All data were tested against the WT reference, the comparison range was set to 70, the WT marker was set to 5, and the minimum frequency was set to 1.

### T7 endonuclease I mismatch detection assay

To analyze CRISPR-Cas–mediated GSH1 editing in worms derived from hamsters infected with potentially transgenic cercariae, a T7 endonuclease I (T7E1) mismatch detection assay (*162*) was performed. T7E1 originates from T7 bacteriophages and cleaves Holliday structures, mismatched DNAs, and heteroduplexes within DNA templates (*163*–*165*). This assay relies on heteroduplex formation between a DNA strand with Cas-induced mutations and an unedited complementary strand. T7E1 cleaves formed heteroduplexes, allowing the confirmation of mosaicism and/or heterozygotic editing through gel electrophoresis, as shown by multiple bands on the gel. The GSH1 locus was amplified by PCR using GSH1 TIDE primer pairs (table S5). The T7 heteroduplex formation reaction combined 200 ng of the PCR product with 2 μl of 10x NEBuffer 2 (NEB) to a final volume of 19 μl. DNA was denatured for 10 min at 95°C, and heteroduplexes were formed by cooling to RT. Samples were digested by adding 1 μl of T7E1 (5 U/μl, NEB) at 37°C for 15 min. T7E1 was inactivated with 1 μl of 0.5 M EDTA. Fragment analysis was performed by gel electrophoresis.

### Verification of transgene integration

The following procedures were previously described in (*141*). After EPO, LEs of experimental and control groups were harvested at 5 and 10 days posttransfection. Genomic DNA of each sample was analyzed by PCR for 5′- and 3′ transgene integration into GSH1. To check the 5′ terminus, we used a primer pair spanning 799 nt of the upstream region of GSH1 and part of the ubiquitin promoter (table S5). For the 3′ terminus, primers spanning 1290 nt of the targeted mutation, specifically a chimeric primer spanning the EGFP coding sequence, and the terminator paired with a primer for the GSH1 were used (table S5). For KI verification, we designed specific primers amplifying the 5′- and 3′-integration sites of the transgene. To this end, primers were developed that bind specifically to GSH1, ensuring that the HAs of the donor templates are not recognized. These primers were each combined with primers specific for the donor DNA to investigate the success of integration (table S5). PCRs were performed using the GoTaq Master Mix DNA polymerase (Promega, Madison, WI), 10 ng of gDNA, and 10 μl of reaction volumes. PCR conditions were 98°C for 5 min, 38 cycles of 95°C for 30 s, 60°C for 30 s, and 72°C for 80 s. Ten biological replicates were examined. The internal control for the PCR was performed by amplifying a 1466-bp fragment using the GSH1-specific primers (Fig. 3B; see also table S5). The PCR conditions did not allow amplification of the 4732-bp fragment, which represents the length of the complete reporter gene. Quantitative PCR (qPCR) was performed by analyzing 10 ng of gDNA (in triplicate) in a 10-μl total volume of the SYBR558 Green PCR Master Mix (Bio-Rad, Hercules, CA) using the Bio-Rad real-time system using 400 nM EGFP-specific primers (table S5). The pJC53.2_SmUbi-EGFP-SmUbi plasmid served as a template for qPCR to generate an external and logarithmic standard curve based on the EGFP copy numbers, which ranged from 2.7 to 1.17 × 10^7^ copies. Copy numbers of integrated EGFP sequences were estimated using qPCR values obtained from 100 ng of gDNA from eggs and interpolation from the standard curve (*14*, *15*).

### Transgene integration analyses

The following procedures were previously described in (*141*). EGFP expression was assessed using CLSM (Zeiss Examiner.Z1 AX10; Camera, Carl Zeiss AxioCam MRc) with an inverse objective at a 20x magnification [Zeiss W Plan-Apochromat 20x/1.0 DIC (UV) VIS-IR 421402-9800], an approach that enabled imaging of the eggs in culture (20% FBS in DMEM) at 509-nm excitation (*15*). Image analysis was accomplished using ZEN 2011 (Zeiss) at days 5 and 10 following transfection using ∼3000 LEs in each replicate. Adult schistosomes obtained after transplantation of potentially transgenic sporocysts into recipient snails were washed twice with PBS immediately after perfusion and fixed for 1 hour with 2% paraformaldehyde. The worms were washed twice with PBS, counterstained with Hoechst (Thermo Fisher Scientific), embedded in ROTI MOUNT FluorCare (Roth), and analyzed using the TCS SP5 VIS confocal laser scanning microscope (Leica Microsystems GmbH, Wetzlar, Germany) and a 63x objective. Single images were stitched using the ImageJ Mosaic tool. Image processing was performed using the LasX software (Leica). Subsequently, egg hatching was induced, or eggs or adult worms were lysed for recovery of DNA and RNA (as above).

Transcripts of the transgene were amplified by RT-PCR. At day 10 following transfection, 3000 LEs of each biological replicate were transferred to 50 μl of RNAzol RT (Molecular Research Center Inc., Cincinnati, OH), homogenized using a motorized pestle, and RNA isolated and quantified. Synthesis of cDNA from 30 μg of total RNA per sample was done using the Maxima First Strand cDNA Synthesis Kit with DNase I (Thermo Fisher Scientific) and used as a template to confirm the presence of transgene transcripts using EGFP-specific primers. Transcripts of the Sm*gapdh* gene served as the reference (table S5), and 10 ng of donor dsDNA served as a positive control. PCR reaction volumes of 25 μl, with 3 μl of cDNA, 1 μM each primer, and GoTaq polymerase reagent (Promega, Madison, WI, USA), were subjected to thermal cycling: 98°C for 3 min, 40 cycles of 95°C for 30 s, 55°C for 30 s, and 72°C for 60 s, and products were assessed as above.

## Supplementary Materials

### The PDF file includes:

Tables S1 to S4 and S7 Legends for tables S5, S6, and S8 to S10 Figs. S1 to S11 References
