# Supplementary material for: Toward transgenic multicellular parasites: CRISPR-Cas12a germline editing of Schistosoma mansoni eggs as key to success
Source: Sci Adv. 2026 Sep 30;12(40):eaeh0455. doi: 10.1126/sciadv.aeh0455 (PMC13626086; doi:10.1126/sciadv.aeh0455)
Supplement: Supplementary file 1 [file sciadv.aeh0455_sm.pdf]

Science Advances
AAAS

Supplementary Materials for

Toward transgenic multicellular parasites: CRISPR-Cas12a germline editing of *Schistosoma mansoni* eggs as key to success

Max F. Moescheid *et al.*

Corresponding author: Max F. Moescheid, max.moescheid@leibniz-fli.de; Paul J. Brindley, pbrindley@gwu.edu; Christoph G. Grevelding, christoph.grevelding@vetmed.uni-giessen.de

Sci. Adv. **12**, eaeh0455 (2026)
DOI: 10.1126/sciadv.aeh0455

The PDF file includes:

Tables S1 to S4 and S7
Legends for tables S5, S6, and S8 to S10
Figs. S1 to S11
References

Other Supplementary Material for this manuscript includes the following:

Tables S5, S6, and S8 to S10

Supplemental Figures and Tables

Supplemental Tables:

Supplemental Table S1. Plasmid card and sequence of the SmUbi-EGFP-SmUbi reporter gene construct.

Plasmid card of the reporter-gene construct

pJC53.2_UbiProm-EGFP-UbiTer (6554 bp; AmpR; KanR)

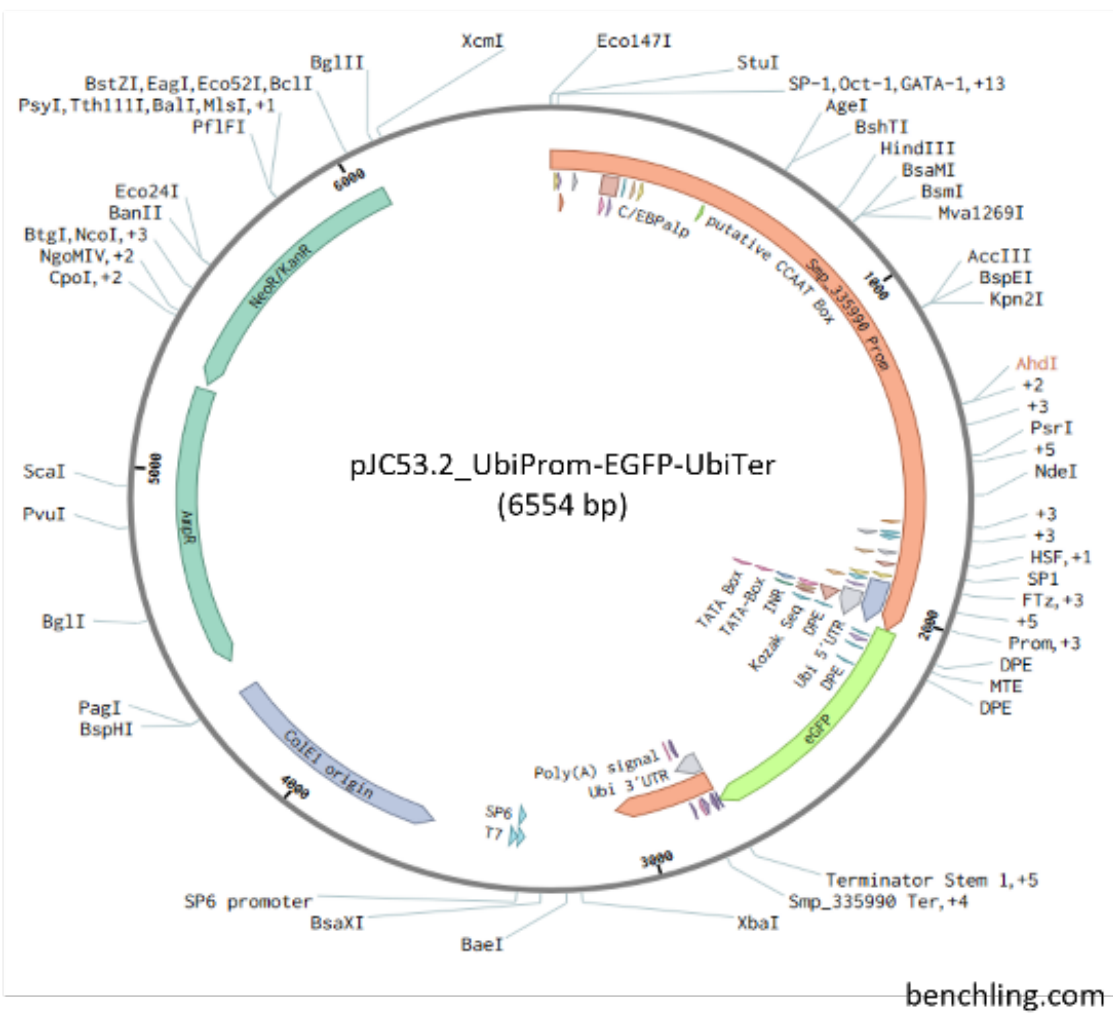

Sequences of the reporter-gene construct

Ubiquitin (Smp_335990) promoter 5´-3´

CCTTTCCAGATTGTCTCCGATTGATTTCACTTATCTCAATCACCACCAGATTGTGTCTTCTCACTTGGACACGATCTGAACGGTCCTCAATTGTGTGCGAACACTTCA
CTTTCCGATATTGGCTGCCATTCTGGTCATTGTGAAGGTCTACGTTTTTGTGGCTCATAAAAAGTCCCAGCTTTCGGCACAAGTAATTGTAAGACTGCCAAACTACC
AAGGAAGAGGTTAAATGACTTATCTGGTAAGTGTTTTTCGGAAATATTTTGCTGTATGGAGTCACTAACCACACACGACCTGTTTTCTCTACTCGAGCTTGAGAAAG
TCACTAACCCAAGAAAATTGAAGCCTGTAATGTTCGTCATCCCTTCTCTTAGCGTTTATCAAGCCCACGAATACGTCCAGCCAAAAGAGAATTATTGCTGCCGGAAT
AGCGGAGGTGAGTGCTTCTAAGTCACTGAGGTTGTCAACATGGTCAATGTAAGGAAACTTCTGACTGTTCCTTCTCTCAGAATCGAGGTAGATAATATGTGGACCA
AGAAATGTGTTTTGTAGACAGCGTATTTTCTGGTGTGTTAGCCTCGTACATAAGCTAACATCAGAGATTCATGGGAAACCGGTGTTGATGTTATCACTAGTGTGAA
TACTAGGGAATGATCTGGGGTTTGGAAAGTATTAGTTGGTCCCATCATTATTCTTCACACTCGGAGCATCAGGGCTTGATGACTTGAAAACGCCATCTTGTGTCTCC
GTGTAAGTGGAAATATAACATAAAATAAGCTTTTTACGTTCGAGACACATATCGACCAGCTTAGTTGGGCAATGAATGCAATCTTCTCTTTGGGATTCAGTCTAGG
AATTTCCCATTTTTAAAGGAGAAAGTTCCTTTAAAATCTTTTTAAGAGTCTAATTTCGAGAAACTACATTAAACAGTGTGCGATTTTATCCTCATGTGTTCGATGAAG
GAGCTAGAAAAACATTACTCGGCAGTCATCCTTCCGCAAAGAAACCATAGTGAGAGGTTGTTGGCGAAAACGTTTTTGCCAGTAAAGATATAATATTAGCAGACCT
GTGACTGGATGCTGATCCCACATTGCAGAATAACTTGACTTCTCCGGACTTGAATTGTCCCTATTAATGAACTCATCTCCTCCACTATATGTGTTCTGAAAAATTAAC
CTAGAAAATATTCAAATATCTTCAGGCTATAATATGAAGTTTCATAGTCTAAGAAAAGAGATTATTCCATCCAAAACCAGATTTTCACGCTCAATTTCAGAACCGTA
GTTCTTGTCAACATTTCTGATGAGTGCTAGTTAGTTTCTGACAAATTGAAGTGATAAGAAAGTAAGAAGTGATGTCGGAAAGAAGATGAGGGTGGTGACAAGCAG
TCGAAATCCAGGAGTCACGGCTAATCCAATGGTCGCACCACTTTGTAAGTCGGAATTCTTATAACCTTCGTGGACAGTCGTGTTATTTTCACGCTCAATTGTTGTGA
ACTGGGTCTTCCAATTTAGCGTCTGCAGAAGTATCTGAACGTCTCTTACTCGCTTAAGTCAGAGACAAACTAGTGAGACATTTTCAAGGTCTTCACAATTGTTTTCT
GTTGATTTAGATGTGAAAAATCATTCATATGATGCACTTGGAGATGTTTAAGTTCCGATCTAACGAAGCATATCTCCAACAGTTAACTGCTCTAATAAATTGATGAC
TTCACGTGAACTAAAACCCATAAAGATAAAATGGCAAACTTTATTTTACATTATTACCTAAACTTTTGTCATCTTGAAACTCGAAAAATGTGGCACTTGAATGAGAA
GTTGCTGACTAACAAAAACGTTTCATAATGAATTAGGAGGTTCAGACTGTAACAAGTTATTTTCTGAAAGTAAATGTGACTAGAATGTGGTTTGAGTAAGTAACAA
TCACCTCGACGAATACCTTGTTTTACTGCCCTGAATTGAAGGTAGCGCTAAATAAACGTAGTATAGACTATATATAGCCAGCAAAATCGAAAGGCTGCCATACTTG
TTCGTTAGTGTAAACGATAGACCTTACG

EGFP-reporter gene 5´-3´

ATGGTGAGCAAGGGCGAGGAGCTGTTCACCGGGGTGGTGCCCATCCTGGTCGAGCTGGACGGCGACGTAAACGGCCACAAGTTCAGCGTGTCCGGCGAGGGCG
AGGGCGATGCCACCTACGGCAAGCTGACCCTGAAGTTCATCTGCACCACCGGCAAGCTGCCCGTGCCCTGGCCCACCCTCGTGACCACCCTGACCTACGGCGTGC
AGTGCTTCAGCCGCTACCCCGACCACATGAAGCAGCACGACTTCTTCAAGTCCGCCATGCCCGAAGGCTACGTCCAGGAGCGCACCATCTTCTTCAAGGACGACG
GCAACTACAAGACCCGCGCCGAGGTGAAGTTCGAGGGCGACACCCTGGTGAACCGCATCGAGCTGAAGGGCATCGACTTCAAGGAGGACGGCAACATCCTGGG
GCACAAGCTGGAGTACAACTACAACAGCCACAACGTCTATATCATGGCCGACAAGCAGAAGAACGGCATCAAGGTGAACTTCAAGATCCGCCACAACATCGAGG
ACGGCAGCGTGCAGCTCGCCGACCACTACCAGCAGAACACCCCCATCGGCGACGGCCCCGTGCTGCTGCCCGACAACCACTACCTGAGCACCCAGTCCGCCCTGA
GCAAAGACCCCAACGAGAAGCGCGATCACATGGTCCTGCTGGAGTTCGTGACCGCCGCCGGGATCACTCTCGGCATGGACGAGCTGTACAAGTG

Ubiquitin (Smp_335990) terminator 5´-3´

GAGGTTAACTTTAATTTTGTTCATTTAACTTAATAAACGTTTAATTTAACTTATTAAAATGTTTTCTTCCTAATTTGTAGCGTATCTTAGTGTAACTGTGCTTCCTATTA
GATTGAAATTCTATCGTAATCTGCAATTAGGCTCATGGGGATATGTTATGCCAATTTTCCGAAAATTGTTAGCACTTAGGTTTCGGTGTTTTTTTACTAGGATGTCAT
GTTTTATTCATATTTGCGTCCTTGCTCTTGTGTGTGGTATGCGTCGCTATGGTCTCAGCTTTGGGCTGCATTGTTAAGTTTGAG

Supplemental Table S2. List of regulatory elements within the predicted Sm*ubi* promoter rank1 sequence.

Predicted promoter element	Abbreviation	5‘ to 3’ sequence	Position	Sources
Promoter Rank 1	**Prom rank 1**	TAGTATAGACTATATATAG CCAGCAAAATCGAAAGGC TGCCATACTTGTT	1981 - 2030 bp	Berkeley Drosophila Genome Project: Neural Network Promoter Prediction
Core Promoter	**Core Prom**	GTAAGTAACAATCACCTC GACGAATACCTTGTTTTAC TGCCCTGAATTGAAGGTA GCGCTAAATAAACGTAGT ATAGACTATATATAGCCA GCAAAATCGAAAGGCTGC CATACTTGTTCGTTAGTGT AAACGATAGACC	1912 – 2051 bp	Butler and Kadonaga 2002
Enhancer Box	**E-Box**	CACGTG	1712 – 1717 bp	Chaudhary and Skinner 1999
CCAAT/enhancer-binding protein binding site	**C/EBPalp**	ACTTTATTTT	1747 – 1756 bp	Kovács *et al*., 2003; AliBaba2.1 TRANSFAC 4.0
Octamer-Binding Transcription Factor 1	**Oct-1**	TATTTTACAT	1751 – 1760 bp	Roberts *et al.,* 1991; AliBaba2.1 TRANSFAC 4.0
CCAAT/enhancer-binding binding site	**C/EBPalp**	TATTACCTAAA	1761 – 1771 bp	Kovács *et al*., 2003; AliBaba2.1 TRANSFAC 4.0
Heat Shock Transcription Factor 1	**HSF**	GAGAAGTTGCTG	1812 – 1823 bp	The Human Gene Database genecards.org; AliBaba2.1 TRANSFAC 4.0
Activator protein 1	**AP-1**	TGCTGACTAA	1819 – 1828 bp	Hess *et al.,* 2004; AliBaba2.1 TRANSFAC 4.0
Transcription Factor SP-1	**SP1**	TAGGAGGTTCA	1850 -1860 bp	The Human Gene Database genecards.org; AliBaba2.1 TRANSFAC 4.0
Fushi Tarazu element	**FTz**	AAATGTGACTA	1888 – 1898 bp	Ohtsuki *et al.,* 1998; AliBaba2.1 TRANSFAC 4.0
GATA-binding factor 1	**GATA-1**	ACTAGAATGT	1895 – 1904 bp	AliBaba2.1 TRANSFAC 4.0
CAAT-Box like sequence	**CAAT-Box**	GTAAGTAACAATC	1912 – 1924 bp	Busek *et al.,* 2002; Juven-Gershon *et al.,* 2010; Godbey 2021
Hepatocyte nuclear factor 1 binding element	**HNF-1**	AACAATCACCT	1918 – 1928 bp	Au *et al.,* 2008; AliBaba2.1 TRANSFAC 4.0
B recognition element	**BRE**	TTGTTTT	1940 – 1946 bp	Ohler *et al.,* 2002; Juven-Gershon *et al.,* 2010; Godbey 2021; YAPP eukaryotic core promoter predictor
TATA-Box containing region	**TATA-Box**	TATAGACTATATATAGCCA	1984 – 2002 bp	Godbey 2021; YAPP eukaryotic core promoter predictor
TATA-Box binding protein	**TBP**	GACTATATATAG	1988 – 1999 bp	AliBaba2.1 TRANSFAC 4.0
CCAAT/enhancer-binding protein binding site	**C/EBPalp**	CAAAATCGAA	2004 – 2003 bp	AliBaba2.1 TRANSFAC 4.0
Serum response factor	**SRF**	GCCATACTTG	2019 – 2028 bp	AliBaba2.1 TRANSFAC 4.0
Transcription Initiator Element	**INR**	CCATACT	2020 – 2026 bp	Godbey 2021; YAPP eukaryotic core promoter predictor
downstream promoter element	**DPE**	AGACC	2047 – 2051 bp	YAPP eukaryotic core promoter predictor
Kozak-Sequence	**Kozak**	CTTACGATG GT Consensus: [N N (T/C) (A/G) N (A/G/T) ATG N (C/A/T) N)]	2051 – 2061 bp	Kozak 1987, Seeber *et al.,* 1997; WEBLOGO tool

Supplemental Table S3. List of regulatory elements within the predicted Sm*ubi* promoter rank2 and 3´UTR sequence.

Predicted promoter element	Abbreviation	*S. mansoni* Sequence 5´- 3	Position in construct (UbiProm-EGFP-UbiTer)	Source
Promoter rank 2	**Prom rank 2**	TTGTGGCTCATAAAAA GTCCCAGCTTTCGGC ACAAGTAATTGTAAGA CTG	156 – 205 bp	Berkeley Drosophila Genome Project: Neural Network Promoter Prediction
Transcription Factor SP-1	**SP-1**	GTCTCCGAT	13 – 21 bp	AliBaba2.1 TRANSFAC 4.0
Octamer-Binding Transcription Factor 1	**Oct-1**	TGATTTCACT	22 -31 bp	AliBaba2.1 TRANSFAC 4.0
GATA-binding factor 1	**GATA-1**	TTATCTCAAT	31-40 bp	AliBaba2.1 TRANSFAC 4.0
CCAAT/enhancer-binding protein binding site	**C/EBPalp**	CACGATCTGA	70 – 79 bp	AliBaba2.1 TRANSFAC 4.0
putative TATA-Box containing region	**TATA-Box**	CTCATAAAAAGT	162 – 173 bp	YAPP eukaryotic core promoter predictor
CCAAT/enhancer-binding protein binding site	**C/EBPalp**	AAGTAATTGT	189 – 198 bp	AliBaba2.1 TRANSFAC 4.0
downstream promoter element	**DPE**	GGTT	224 – 227 bp	YAPP eukaryotic core promoter predictor
CCAAT/enhancer-binding protein binding site	**C/EBPgam**	TTTCGGAAAT	251 – 260 bp	AliBaba2.1 TRANSFAC 4.0
Activator protein 1	**AP-1**	AGTCACTAAC	275 – 284 bp	Hess *et al.*, 2004; AliBaba2.1 TRANSFAC 4.0
CAAT-Box like sequence	**CAAT-Box**	GGTCAATGT	471 – 479 bp	Godbey 2021; AliBaba2.1 TRANSFAC 4.0

Predicted terminator element	Abbreviation	*S. mansoni* Sequence 5´- 3	Position in construct (UbiProm-eGFP-UbiTer)	Source
mRNA 3´Untranslated Region	**3´UTR**	GAGGTTAACTTTAATTTTGT TCATTTAACTTAATAAACGT TTAATTTAACTTATTAAAAT GTTTTCTTCCTAATTTGTAG C	2777 – 2857 bp	Berriman *et al.*, 2009; Protasio *et al.*, 2012
Polyadenylation signal	**Poly(A)**	TGA (52 bp) ATTAAA (21 bp) GC	2774 – 2857 bp	DNAFSMiner-DNA Functional Site Miner polyadenylation side prediction too
Terminator Stem-loop 1	**TL1**	AGAG	2776 – 2779 bp	RNAfold Webserver: Vienna tools
Terminator Stem-loop 1	**TL1**	CUUU	2785 – 2788 bp	RNAfold Webserver: Vienna tools
Terminator Stem-loop 2	**TL2**	UUUUGUUCA	2791 – 2799 bp	RNAfold Webserver: Vienna tools
Terminator Stem-loop 3	**TL3**	AAACGUUUAAU	2811 – 2821 bp	RNAfold Webserver: Vienna tools
Terminator Stem-loop 3	**TL3**	AUU	2829 – 1831 bp	RNAfold Webserver: Vienna tools
Terminator Stem-loop 3	**TL3**	AAAUGUUU	2833 – 2840 bp	RNAfold Webserver: Vienna tools
Terminator Stem-loop 2	**TL2**	UGUAGCAAAA	2852 – 2857 bp	RNAfold Webserver: Vienna tools

Supplemental Table S4. Sequencing results of Cas12a mediated SmUbi-EGFP-SmUbi integration into GSH1 of adult worms from transplantation experiments.

1. 3´-integration side sequence (5´- 3´):

AACGTTTAATTTAACTTATTAAAATGTTTTCTTCCTAATTTGTAGCGTATCTTAGTGTAACTGTGCTTCCTATTAGATTGAAATTC
TATCGTAATCTGCAATTAKGCTCATGGGGATATGTTATGCCAATTTTCCGAAAATTGTTAGCACTTAGGTTTCGGTGTTTTTTA
CTAGGATGTCATGTTTTATTCATATTTGCGTCCTTGCTCTTGTGTGTGGTATGCGTCGCTATGGTCTCAGCTTTGGGCTGCATTGT
TAAGTTTAAGGAAACTGCATAGTATGGAACCAAGGCAGTGGTCACAGAGTAGTATAAYAARATTYTCCTTCAGGCTGCCCACC
AGCACAAARAATGACATAATCGATAGKTACRTCCCCTCMGYTGGAGGGTTTGACCTACGACAGCATGATYTAATATATTTTCR
AGAAAAGYCTAWGATGAAATCGAMCGAATGATGTTAAATCMWACAGGAAAACATTGTTTCGAAGGGGAGAATAAWAGGAG
GGGCTTTWCTMTKAAGGTCCATTAAAGATTTCCTGGATATGCACCACTGCATGTGGCTAATATTTTRTCTCCTGCGACCCTCAA
TMARGAGTCTCAATACKGCTAACCCAWAAGTCTATAATWTCAGYACTAATAGCATATCTTGMTCTGACTTCCAACATCATTCT
TATCRTTCTACTTGCAAGTCRCCCAACATAWAGGCGCTGKATGRRTAGATCCTTACATMSCCCAACCACCTGTGATGTTTACAA
ATTCATTCKKCCTAATACCATCTGTATGTAGGACTTTTCTTAACATCATTGT

2. 3´-integration side sequence (5´- 3´):

YCTAAATGTTTTCTTCCTAATTTGTAGCGTATCTTAGTGTAACTGTGCTTCCTATTAGATTGAAATTCTATCGTAATCTGCAATT
AGGCTCATGGGGATATGTTATGCCAATTTTCCGAAAATTGTTAGCACTTAGGTTTCGGTGTTTTTTACTAGGATGTCATGTTTT
ATTCATATTTGCGTCCTTGCTCTTGTGTGTGGTATGCGTCGCTATGTGGAACTCGGTAGATGAAGGCTGGTTCAATAGCTGGAA
TTGGGATGGTGTGCCACAAGTGTTTAAGGAAACTGCATAGTATGGAACCAGCCTTCATCTACCGAGTTCCACATAGCGACGCA
TACCACACACAAGAGCAAGGACGCAAATATGAATAAAACATGACATCCTAGTAAAAAAACACCGAAACCTAAGTGCTAACAA
TTTTCGGAAAATTGGCATAACATATCCCCATGAGCCTAATTGCAGATTACGATAGAATTTCAATCTAATAGGAAGCACAGTTAC
ACTAAGATACGCTACAAATTAGGAAGAAAACATTTTAATAAGTTAAATTAAACGTTTATTAAGTTAAATGAACAAAATTAAAG
TTAACCTCTCACTTGTACAGCTC

3. 3´-integration side sequence (5´- 3´):

ARCGKATCTTAGTGTAACTGTGCTTCCTATTAGATTGAAATTCTATCSTAATCTGCAATTAKGCTCATGGGGATATGTTATGCCA
ATTTTCCGAAAATTGTTAGCACTTAGGTTTCGGTGTTTTTTACTAGGATGTCATGTTTTATTCATATTTGCGTCCTTGCTCTTGT
GTGTGGWATGCGTCGCTATGGTCTCASCTTTGGGCTGCATTGTTAAGTTTAAGGAAACTGCATAGTATGGAACCAGAG

1. 5´-integration side sequence (5´- 3´):

TTGAGATAAGTGAAATCAATCGGAGACAATCTGGAAAGGTCAGATAATTGATCTCGAGGTATTTTCATAGCATAGGAAATACA
CAATCCG

2. 5´-integration side sequence (5´- 3´):

GCGTATCTTAKTGTAACTGTGCTTCCTATTAGATWGAAATTCTATCSKAATCTGCAATTAKGCTCATGGGGATATGTTATGCCA
ATTTTCCKAAAATTGTTASCACTTAGGTTTCGGTGTTTTTTTACTAGGATGTCATGTTTTATTCATATTTGCGTCCTTGCTCYTGT
GTGTGGWATGCGTCGCTATGTGR

3. 5´-integration side sequence (5´- 3´):

CTTATCTCAATCACCACCAGATTGTGTCTTCTCACTTGGACACGATCTGAACGGTCCTCAATAATG

Supplemental Table S5. List of primers.
See additional Table S5

Supplemental Table S6. *S. mansoni* NHEJ HDR machinery.
See additional Table S6

Supplemental Table S7. Sequence information of the Sm*ubi* minimal promoter.

**Sequence of the Smp_335990 (SmUbi) minimal promoter 5´- 3´ (503 bp)**
TCAGAGACAAACTAGTGAGACATTTTCAAGGTCTTCACAATTGTTTTCTGTTGATTTAGATG<br>TGAAAAATCATTCATATGATGCACTTGGAGATGTTTAAGTTCCGATCTAACGAAGCATATCT<br>CCAACAGTTAACTGCTCTAATAAATTGATGACTTCACGTGAACTAAAACCCATAAAGATAAA<br>ATGGCAAACTTTATTTTACATTATTACCTAAACTTTTGTCATCTTGAAACTCGAAAAATGTGG<br>CACTTGAATGAGAAGTTGCTGACTAACAAAAACGTTTCATAATGAATTAGGAGGTTCAGAC<br>TGTAACAAGTTATTTTCTGAAAGTAAATGTGACTAGAATGTGGTTTGAGTAAGTAACAATCA<br>CCTCGACGAATACCTTGTTTTACTGCCCTGAATTGAAGGTAGCGCTAAATAAACGTAGTAT<br>AGACTATATATAGCCAGCAAAATCGAAAGGCTGCCATACTTGTTCGTTAGTGTAAACGATA<br>GACCTTACG

Supplemental Table S8. Genome-wide prediction of CRISPR/Cas off-target sites.
See additional Table S8

Supplemental Table S9. Tide analysis of Cas9 and Cas12a mediated editing of GSH1.
See additional Table S9

Supplemental Table S10. Detection of EGFP-positive miracidia in LEs following transformation and integration experiments.

Supplemental Figures

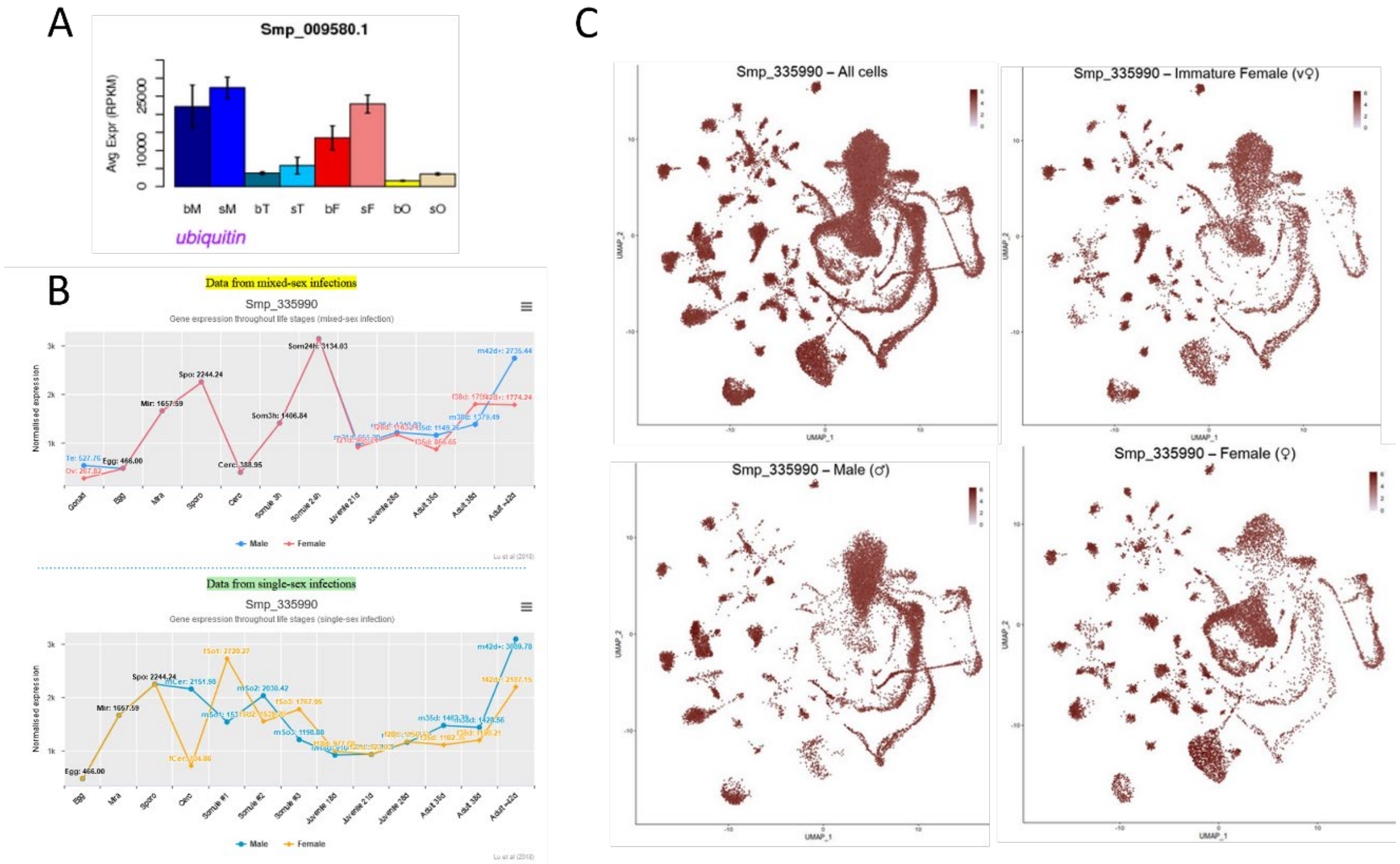

Supplemental Figure S1. Transcriptome data for Smp_335990 (SmUbi) revealed its ubiquitous transcript pattern in both sexes and all cell types in all developmental stages of *S. mansoni.* Compilation of expression data for Smp_335990 (SmUbi) based on different RNAseq data sets that comprised (Panel (**A**)) transcript levels in adult schistosome and their gonads (*19*). **B**, The transcript levels across all life stages of (paired (top) and unpaired (bottom) *S. mansoni*, and the cell type-independent transcript profile of Smp_335990 as disclosed in the single-cell atlas of *S. mansoni* (*35,90*). Abbreviations: bM, bisex male (pairing experienced male); sum, single-sex males pairing unexperienced male), bT, testes of bM; sT, testes of sM; bF, bisex female (pairing experienced female); sF, single-sex (pairing unexperienced female); bO, ovary of bF; sO, ovary of sF.

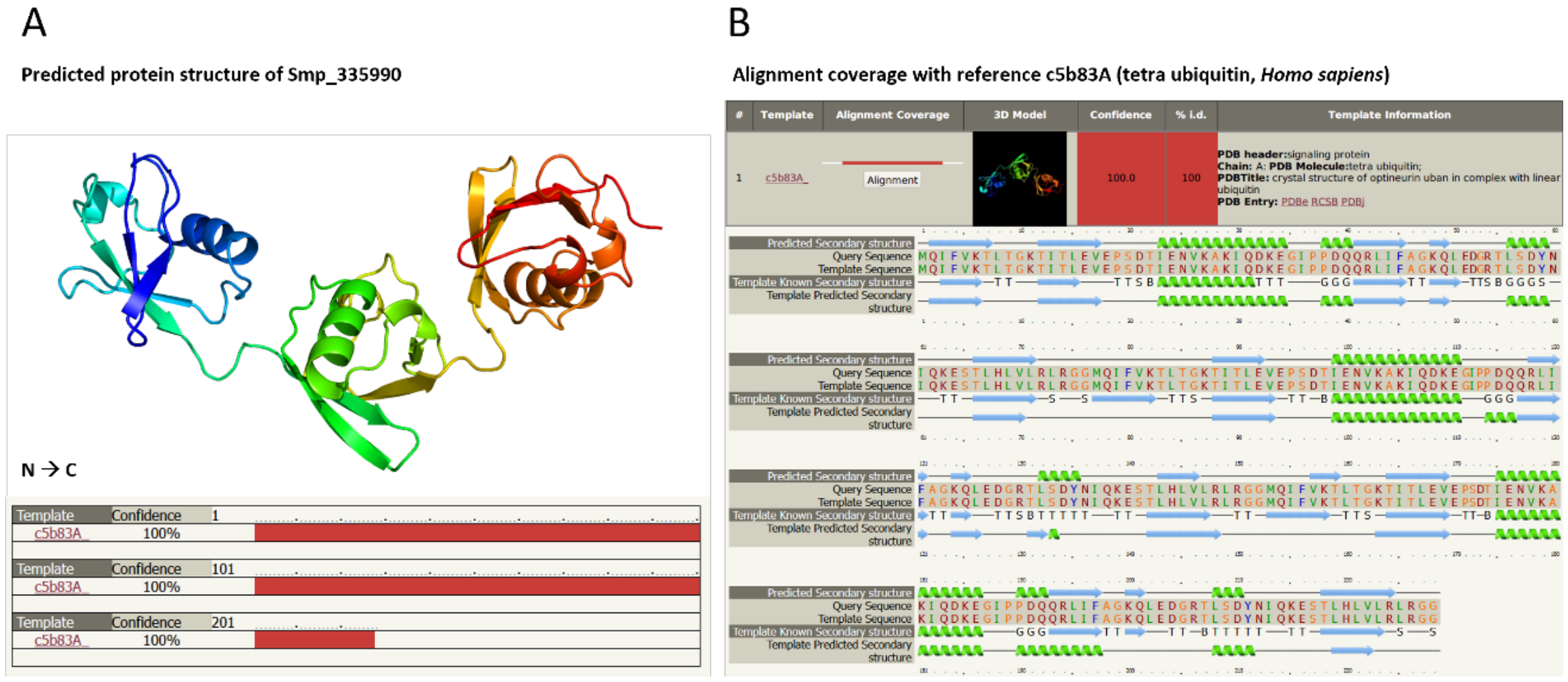

Supplemental Figure S2. Protein structure modelling and sequence analysis confirmed Smp_335990 (SmUbi) as an orthologue of human tetra-ubiquitin. A, The protein structure of SmUbi was predicted using Phyre2 (https://doi.org/10.1016/j.retram.2022.103333) based on known structures of orthologous ubiquitin proteins (*23*). **B**, The amino acid sequence of SmUbi was compared to tetra-ubiquitin orthologs from model species, including *Homo sapiens*, using Phyre2. Using alignment coverage, SmUbi was verified as an ortholog of human tetra-ubiquitin.

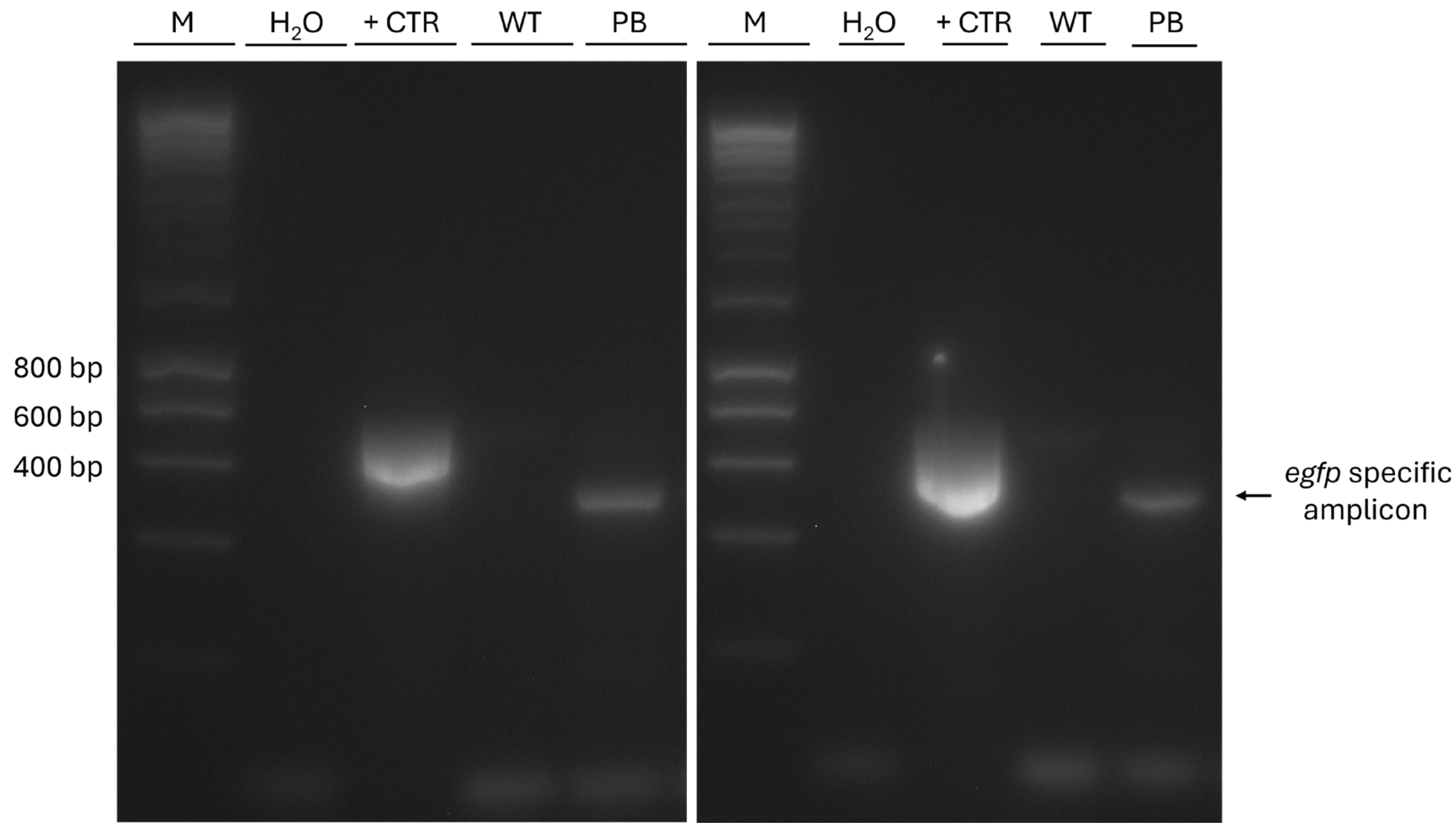

Supplemental Figure S3. Verification of the promoter activity of *S. mansoni* ubiquitin (SmUbi) promoter and terminator elements driving EGFP expression. Original gel images verifying *egfp* transcription after transformation of adult *S. mansoni* with the SmUbi-EGFP-SmUbi reporter-gene by particle bombardment (Figure 2 B-E). The gel images represent the verification of reporter-gene expression of two independent biological replicates of DNase treated RNA by RT-PCR. Abbreviations: CTR, control; H_2O, water control; M, marker; PB, parcticle bombardment; WT, wildtype control.

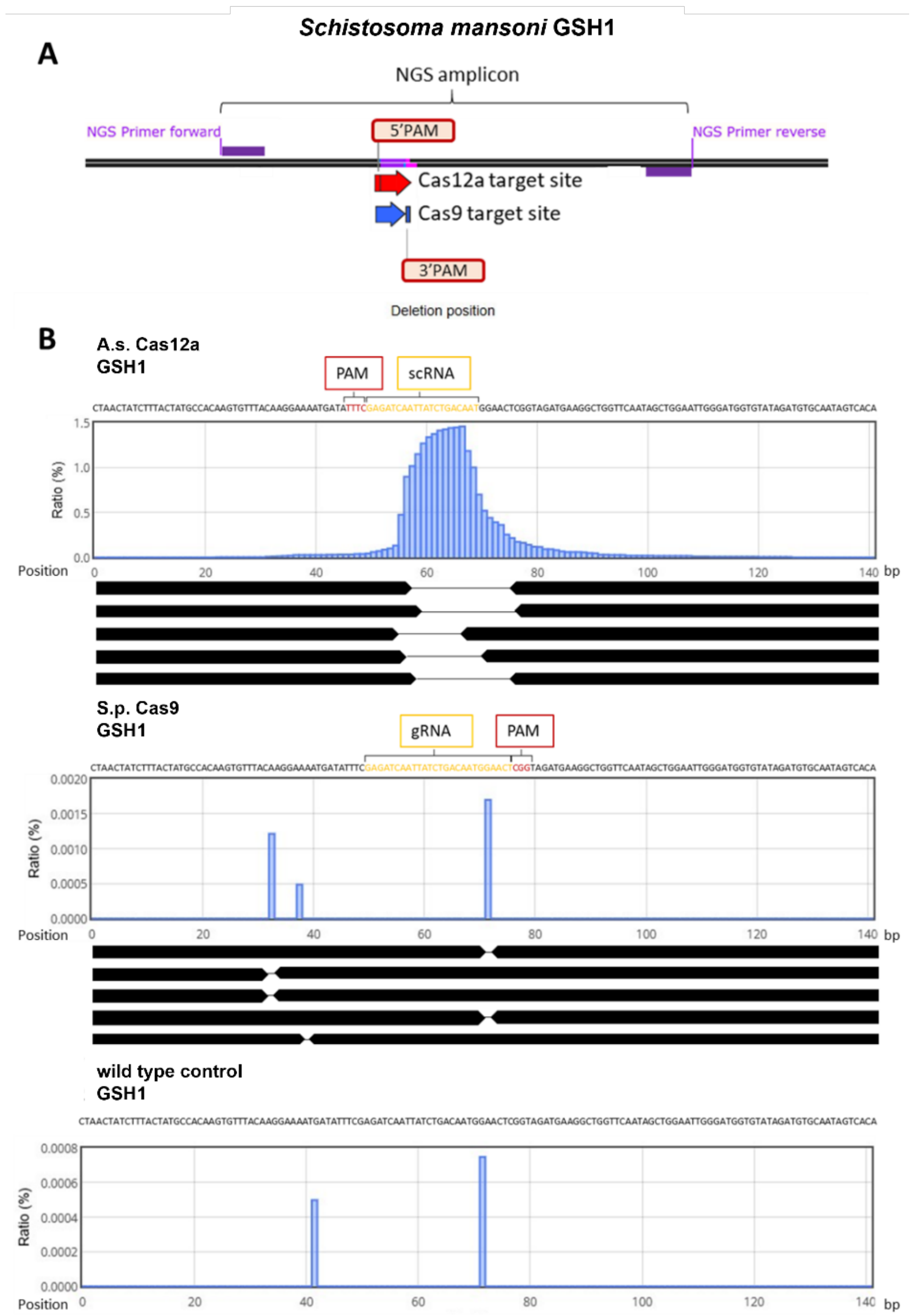

Supplemental Figure S4. AsCas12a RNPs delivered higher editing efficiency and deletion of long DNA fragments compared with Cas9. A, Schematic illustration of the GSH1 locus including the predicted Cas12a (red) and Cas9 (blue) target sites, and amplicon analyses by NGS (purple). **B**, gRNA binding site (yellow) and PAM (red) at the GSH1 locus for the corresponding Cas12a or Cas9 enzymes. The individual graphs (blue) show the deletion events at the respective position in percentage. The editing efficiency per position by Cas12a was 100x higher compared to Cas9-based editing. Representative edited single reads of the sequencing are shown in black, whereby the gaps illustrate deletion events. Abbreviations: gRNA, guide RNA; NGS, next generation sequencing; PAM, protospacer adjacent motif.

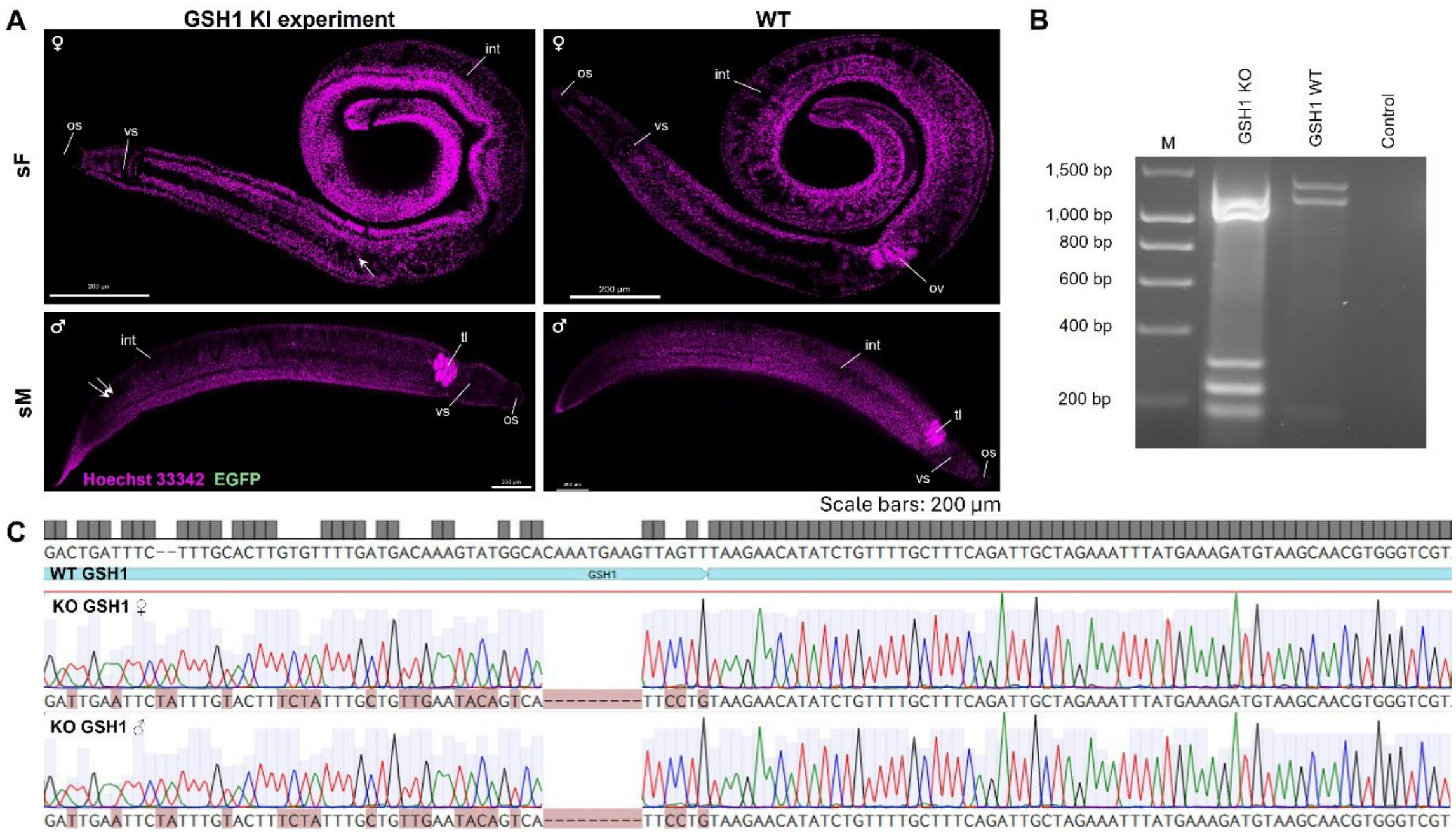

Supplemental Figure S5. Inherited base deletions at GSH1 following attempts at Cas12a-mediated transgene integration. A, Adult worms were counterstained with Hoechst 33342 and analysed by CLSM. Treated worms showed no green, fluorescent signals distributed throughout the tissue. Only some single green, fluorescing spots (indicated by arrows) were found. **B,** Restriction analysis of the GSH1 fragment PCR-amplified from EGFP using the T7E1 approach resulted in the appearance of several bands compared to the corresponding sequence amplified from WT DNA. This indicated successful genome editing and demonstrated successful base deletions at GSH1. The control sample represented a PCR/restriction digest without the addition of DNA. **C,** Amplified GSH1 fragments of a potentially transgenic female and male worm was further analysed by Sanger sequencing. Sequencing results identified several base substitutions and a 9 bp base deletion at the target site. This indicated Cas12a-induced KO of the target locus but no KI if the reporter gene. Sequence alignment was performed with Benchling (Benchling, USA). Abbreviations: WT, wild type; sF, S. mansoni females without pairing experience; sM, S. mansoni males without pairing experience; int, intestine; os, oral sucker; o, ovary; tl, testicular lobes; vs, ventral sucker; KI, Knock-in; KO, knock-out; bp, base pair.

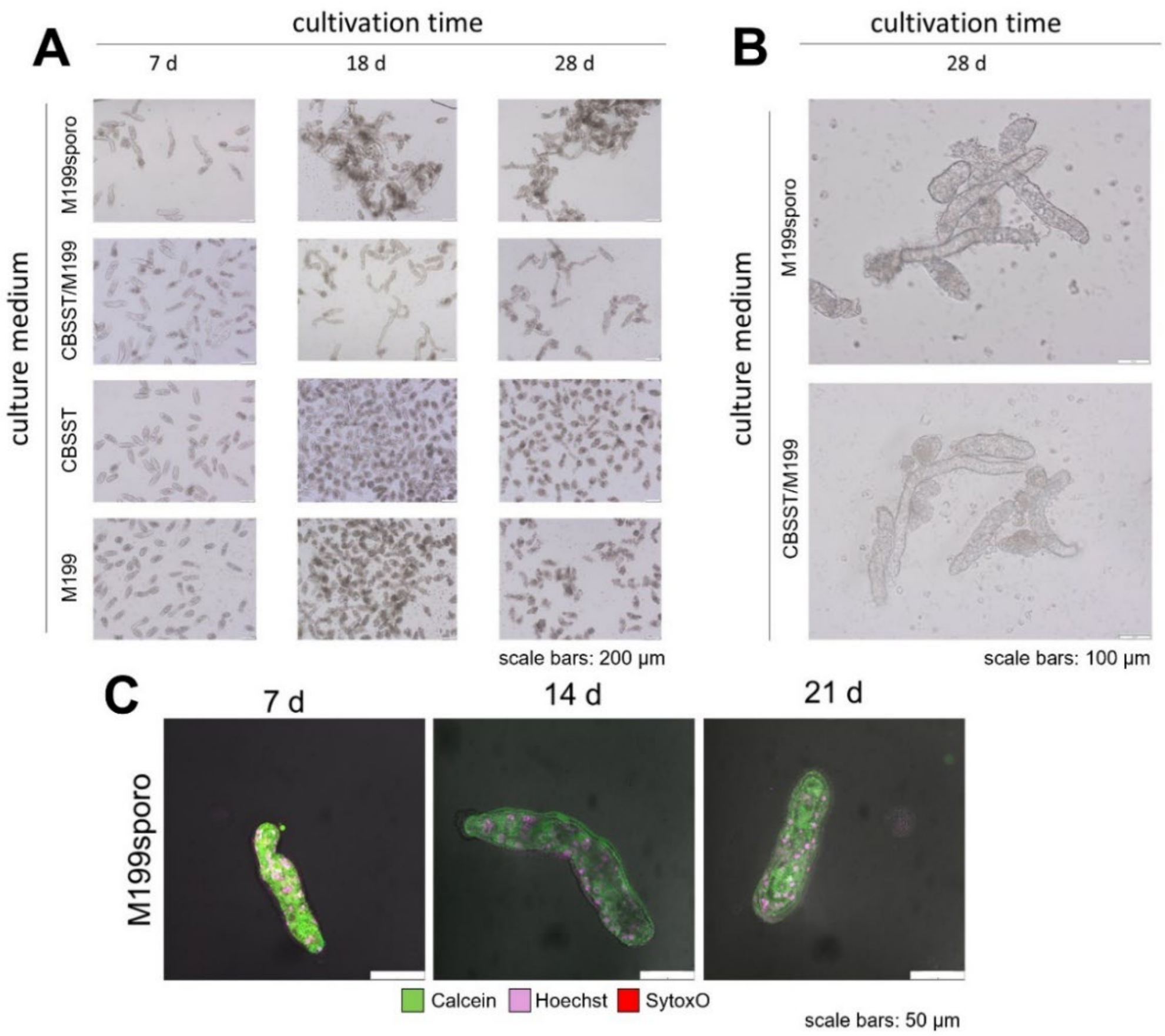

Supplemental Figure S6. Optimized cultivation conditions allowed long-term *in vitro* maintenance of sporocysts. Miracidia were cultured *in vitro* to induce transformation into sporocysts. Cultivation at low oxygen tension (5% O_2), 5% CO_2, 90% N_2 at 26°C was used for explants of snail tissue harboring sporocysts (*54,55*). A, To obtain Bge-cell-free culture of sporocysts (*143, 144, 166*), *in vitro* conditions for sporocysts maintenance and growth, different media were used in our study. The survival and growth of sporocysts were observed over a period of 28 d. Cultivation of sporocysts in M199 without the addition of HEPES and NCS and cultivation in CBSST showed considerably reduced viability, marked by a compressed, dark appearance, after seven and 18 d, respectively. Sporocysts cultured in CBSST/M199 at a 1:1 ratio remained viable at day 28 and showed longitudinally growth. Optimal conditions in terms of sporocyst viability, morphology, and growth were seen with sporocysts cultured in the M199sporo medium, which in comparison to CBSST/M199 additionally contained 5% 7 d Bge cell-conditioned StemMACS iPS-Brew XF. B, Detailed images of viable sporocysts after 28 d. C, Sporocysts were cultured in M199sporo and stained with a life death cell stain (HSC stain). Calcein (green) stained vital, esterase-active cells, while SytoxO (red) stained permeabilized, dead cells, which were not found. Cell nuclei were counterstained with Hoechst 33342 (purple). Sporocysts remained viable throughout the experiment.

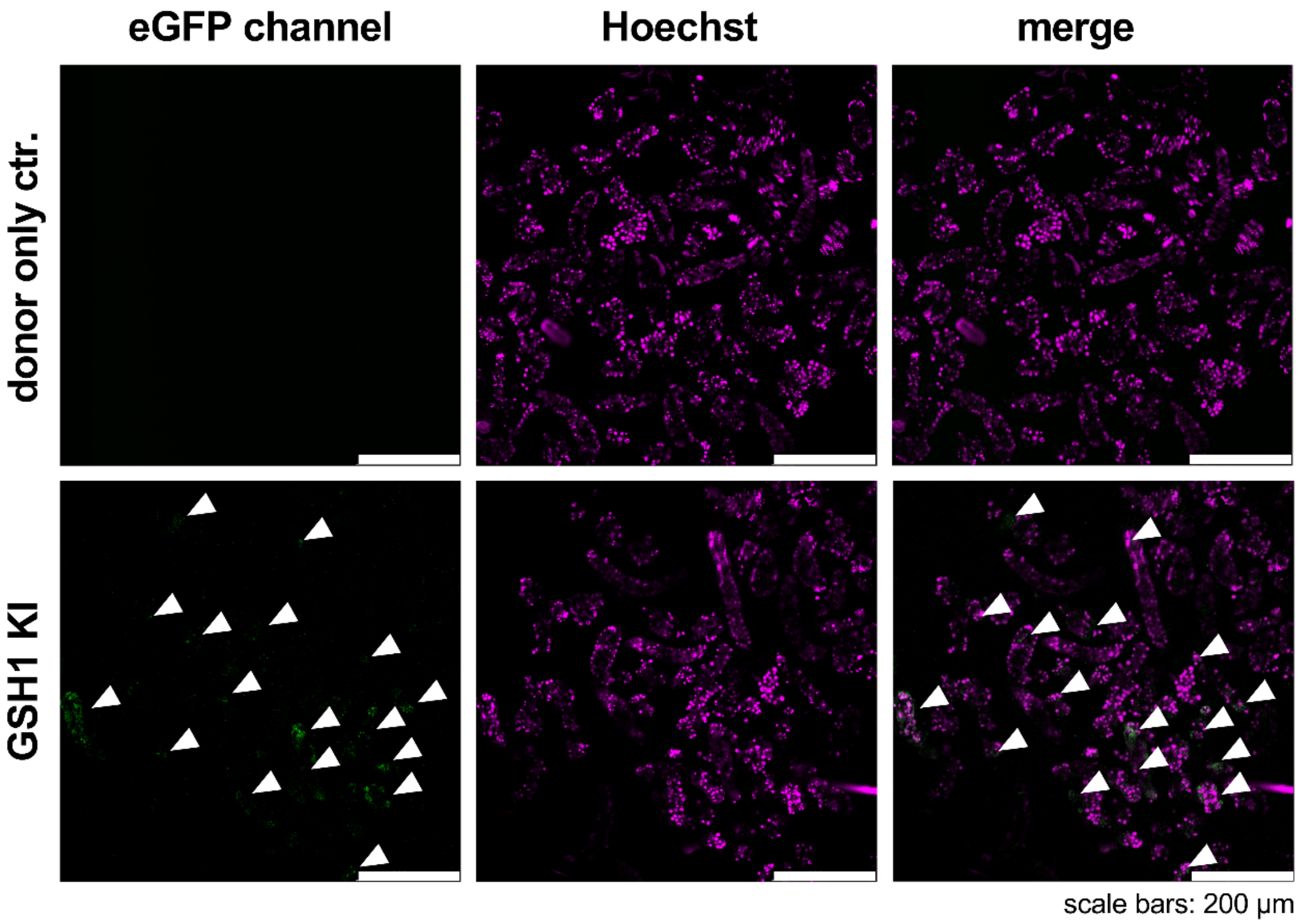

Supplemental Figure S7. Detection of EGFP specific signals in sporocysts derived from transformed, potential transgenic miracidia. Overview of 4 d old sporocysts derived from miracidia of KI experiments using the SmUbi-EGFP-SmUbi transgene with 5'-C6-PEG10-modified microhomology arms and Cas12a RNPs. Sporocysts were counterstained with Hoechst33342 (purple) and analyzed using CLSM. In control sporocysts, which were derived from LEs only transfected with donor DNA, no eGFP signals were detected; eGFP-positive signals were only identified in sporocysts of the KI group. Sporocyst showing either EGFP signals in all cells or in a mosaic pattern are indicated by white arrows. Abbreviations: ctr, control; KI, knock-in.

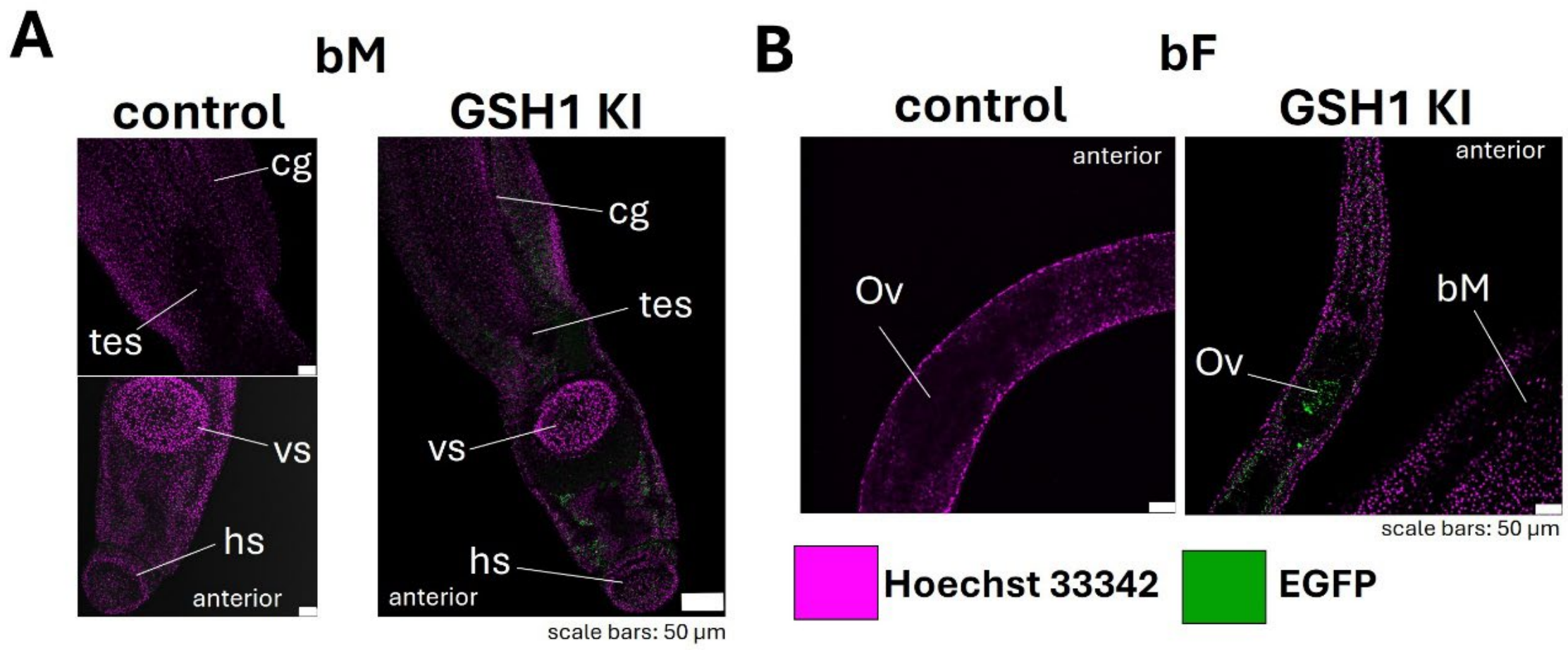

Supplemental Figure S8. CLSM analysis showing EGFP expression in adult *S. mansoni* following GSH1 editing of schistosome eggs, mono-miracidial snail infection, cercariae shedding, and infection of hamsters. Adult *S. mansoni* were recovered from hamsters infected with cercariae which resulted from mono-miracidial snail infection with potentially transgenic miracidia. For KI editing, we used a 5´C6-PEG10-modified SmUbi-EGFP-SmUbi donor, which induced HDR by 50 bp (HAs). As biological source for transformation we used LEs that were electroporated with Cas12a RNPs and donor DNA to target GSH1. Control worms were obtained from donor DNA only treated LEs as starting material. CLSM of living males (**A**) and females (**B**) exhibited EGFP expression only in worms from KI experiments but not in control worms. Worms were counterstained with Hoechst33342 (purple). **A**, EGFP signals were found in the parenchyma of males from KI experiments and not in the control. **B**, CLSM analysis revealed EGFP signals in the parenchyma and ovaries (Ov of paired females (bF) obtained from KI experiments. No EGFP signals were found in the ovaries of control females.

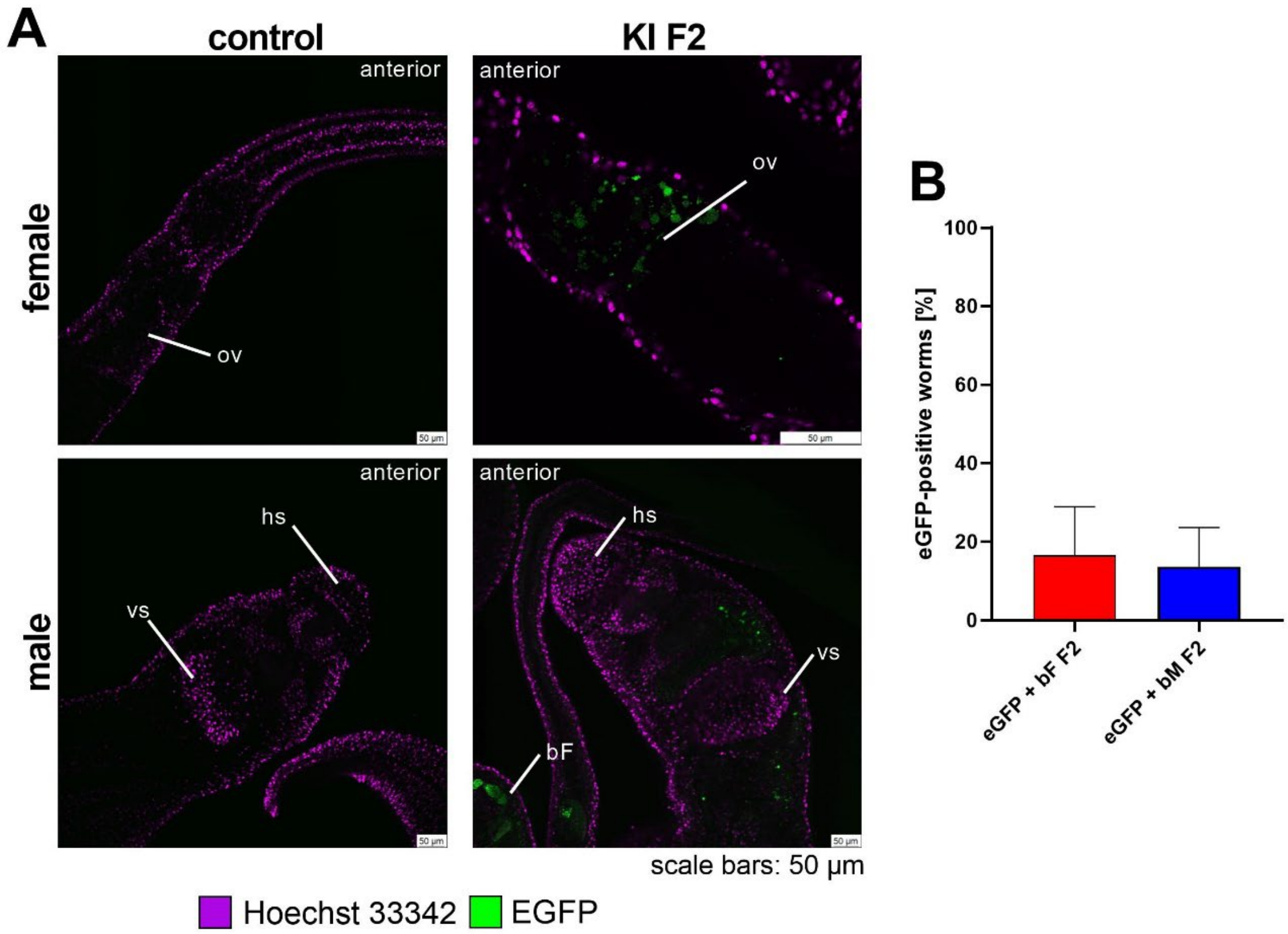

Supplemental Figure S9. EGFP-positive schistosomes also occur in filial generation. To investigate transgene inheritance, clonal cercariae of the F0 generation (obtained from transplantation experiments with EGFP-positive sporocysts), were mixed with (unedited) wild-type cercariae for final host infection. Cercariae obtained from this "founder" experiment were used for hamster infection A, In the resulting filial generation, EGFP-positive worms of both sexes were observed. Furthermore, EGFP signals were also found in oocytes of female ovaries. B, The number of EGFP-positive worms was determined with worms being considered positive if they displayed EGFP expression in at least 50% of their tissue and/or in the gonads. Abbreviations: bF, pairing experienced females; hs, head sucker; F0, filial generation 0; KI, knock-in; ov, ovary; vs, ventral sucker.

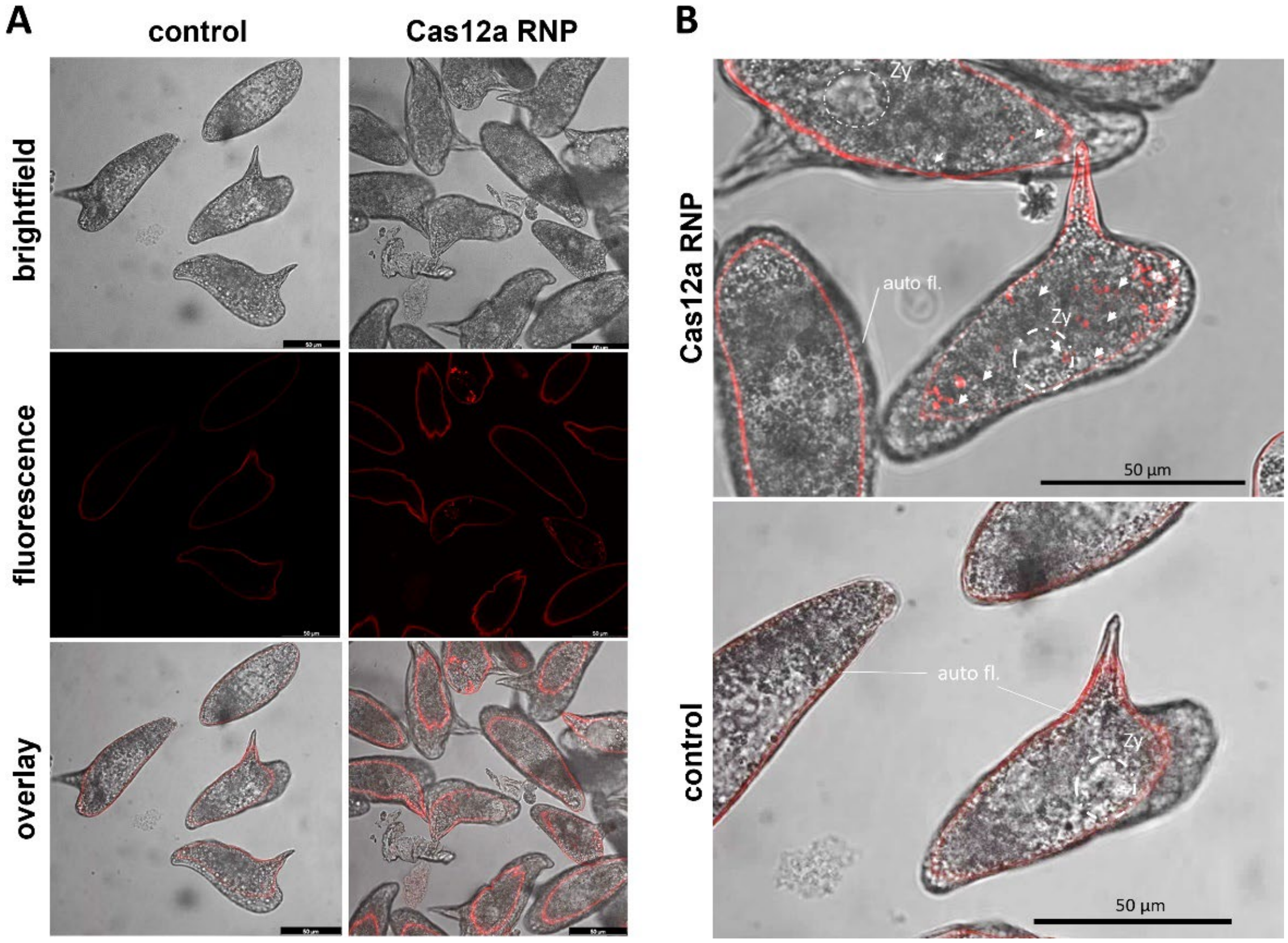

Supplemental Figure S10. Transfection of eggs at an early developmental stage using labeled CRISPR/Cas12a RNPs. A, CLSM analysis of early, *in vitro* laid schistosome eggs (0-24 h, IVLEs (*17*)) 2 h after transfection with RNPs containing rhodamine-labelled Cas12a-gRNAs via electroporation. The control IVLEs were electroporated in the absence of RNPs. In control IVLEs, only autofluorescence of the eggshell was observed. Fluorescent signals, specific for the rhodamine-labelled RNPs, were detected on the eggshell and in the cells of various RNP-treated IVLEs. **B**, Detailed image of IVLEs transfected with rhodamine-labelled Cas12a RNPs 2 h after electroporation. Successful delivery of CRISPR/Cas material (arrows) into numerous cells of the IVLEs was observed. In some IVLEs, RNPs were detected in the zygote. Fluorescence spectra were acquired using a Leica TCS SP5 vis confocal laser scanning microscope with Leica LasX software. Scale bars: 50 µm. Abbreviations: auto fl., auto fluorescence; CLSM, confocal laser scanning microscopy; Zy, Zygote.

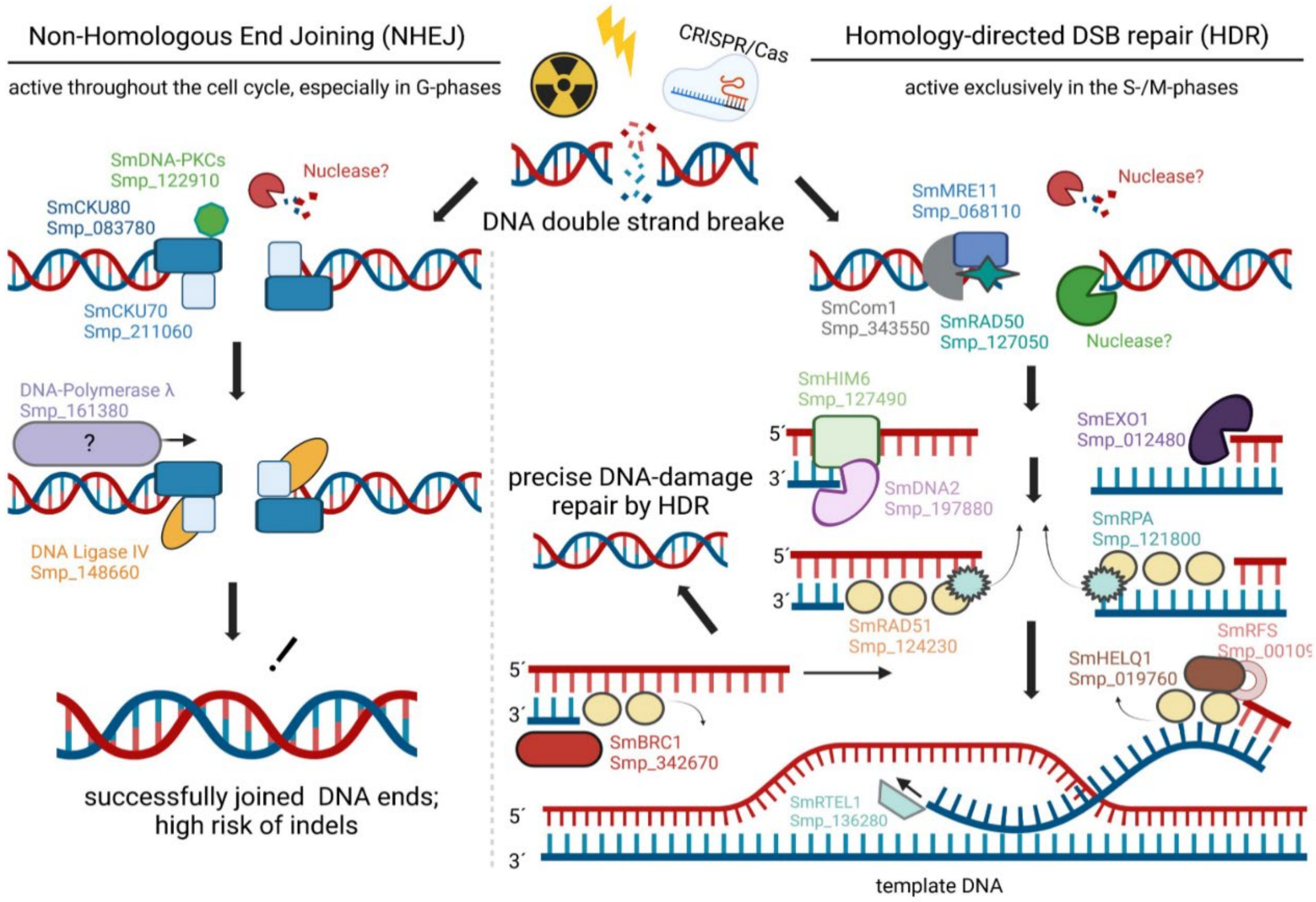

Supplemental Figure S11. Schematic overview of predicted DSB-repair pathways in *S. mansoni.* Orthologs to the key actors of the double-stranded break (DSB)-repair pathway, as described for *Caenorhabditis elegans* (*83, 84*) were found in *S. mansoni*. Consequently, orthologs essential for both NHEJ and HDR were identified. For this purpose, the protein sequences of previously characterized DSB repair proteins from the nematodes *Caenorhabditis elegans* and *Pristionchus pacificus* were analyzed using BLAST against the proteome of *S. mansoni* (parasite.wormbase.org, genome V10). Furthermore, both the sequence coverage and domain structures of the analyzed proteins were compared using the SMART Domain analyzer (smart.embl-heidelberg.de, and orthologs were annotated as described *(83)*). Created in Biorender. Möscheid, M. (2026) https://BioRender.com/7xyu8tp

REFERENCES

1. B. Zhang, CRISPR/Cas gene therapy. *J. Cell. Physiol.* **236**, 2459–2481 (2021).

2. D. Zhang, Z. Zhang, T. Unver, B. Zhang, CRISPR/Cas: A powerful tool for gene function study and crop improvement. *J. Adv. Res.* **29**, 207–221 (2020).

3. K. Oonuma, R. Kuroda, T. Uchida, S.-M. Zhang, CRISPR/Cas9-germline editing of *Biomphalaria glabrata*: A breakthrough in genetic modification of snails that transmit schistosomiasis. *Sci. Adv.* **11**, eadx5889 (2025).

4. K. S. Makarova, Y. I. Wolf, J. Iranzo, S. A. Shmakov, O. S. Alkhnbashi, S. J. J. Brouns, E. Charpentier, D. Cheng, D. H. Haft, P. Horvath, S. Moineau, F. J. M. Mojica, D. Scott, S. A. Shah, V. Siksnys, M. P. Terns, Č. Venclovas, M. F. White, A. F. Yakunin, W. Yan, F. Zhang, R. A. Garrett, R. Backofen, J. van der Oost, R. Barrangou, E. V. Koonin, Evolutionary classification of CRISPR-Cas systems: A burst of class 2 and derived variants. *Nat. Rev. Microbiol.* **18**, 67–83 (2020).

5. A. Pickar-Oliver, C. A. Gersbach, The next generation of CRISPR-Cas technologies and applications. *Nat. Rev. Mol. Cell Biol.* **20**, 490–507 (2019).

6. L. Cong, F. A. Ran, D. Cox, S. Lin, R. Barretto, N. Habib, P. D. Hsu, X. Wu, W. Jiang, L. A. Marraffini, F. Zhang, Multiplex genome engineering using CRISPR/Cas systems. *Science* **339**, 819–823 (2013).

7. M. Jinek, K. Chylinski, I. Fonfara, M. Hauer, J. A. Doudna, E. Charpentier, A programmable dual-RNA-guided DNA endonuclease in adaptive bacterial immunity. *Science* **337**, 816–821 (2012).

8. P. Mali, L. Yang, K. M. Esvelt, J. Aach, M. Guell, J. E. DiCarlo, J. E. Norville, G. M. Church, RNA-guided human genome engineering via Cas9. *Science* **339**, 823–826 (2013).

9. B. Paul, G. Montoya, CRISPR-Cas12a: Functional overview and applications. *Biomed. J.* **43**, 8–17 (2020).

10. D. C. Swarts, M. Jinek, Cas9 versus Cas12a/Cpf1: Structure-function comparisons and implications for genome editing. *Wiley Interdiscip. Rev. RNA* **9**, e1481 (2018).

11. H. Kim, S.-T. Kim, J. Ryu, B.-C. Kang, J.-S. Kim, S.-G. Kim, CRISPR/Cpf1-mediated DNA-free plant genome editing. *Nat. Commun.* **8**, 14406 (2017).

12. V. Nekrasov, B. Staskawicz, D. Weigel, J. D. G. Jones, S. Kamoun, Targeted mutagenesis in the model plant *Nicotiana benthamiana* using Cas9 RNA-guided endonuclease. *Nat. Biotechnol.* **31**, 691–693 (2013).

13. E. P. Papapetrou, A. Schambach, Gene insertion into genomic safe harbors for human gene therapy. *Mol. Ther.* **24**, 678–684 (2016).

14. W. Ittiprasert, M. F. Moescheid, C. Chaparro, V. H. Mann, T. Quack, R. Rodpai, A. Miller, P. Wisitpongpun, W. Buakaew, M. Mentink-Kane, S. Schmid, A. Popratiloff, C. G. Grevelding, C. Grunau, P. J. Brindley, Targeted insertion and reporter transgene activity at a gene safe harbor of the human blood fluke, *Schistosoma mansoni*. *Cell Rep. Methods* **3**, 100535 (2023).

15. W. Ittiprasert, M. M. Moescheid, V. H. Mann, P. J. Brindley, Multiplexed CRISPR-Cas9 protocol for large transgene integration into the *Schistosoma mansoni* genome. *STAR Protoc.* **5**, 102886 (2024).

16. W. Ittiprasert, C. Chatupheeraphat, V. H. Mann, W. Li, A. Miller, T. Ogunbayo, K. Tran, Y. N. Alrefaei, M. Mentink-Kane, P. J. Brindley, RNA-guided AsCas12a- and SpCas9-catalyzed knockout and homology directed repair of the Omega-1 locus of the human blood fluke, *Schistosoma mansoni*. *Int. J. Mol. Sci.* **23**, 631 (2022).

17. A. D. Jurberg, T. Gonçalves, T. A. Costa, A. C. A. de Mattos, B. M. Pascarelli, P. P. A. de Manso, M. Ribeiro-Alves, M. Pelajo-Machado, J. M. Peralta, P. M. Z. Coelho, H. L. Lenzi, The embryonic development of *Schistosoma mansoni* eggs: Proposal for a new staging system. *Dev. Genes Evol.* **219**, 219–234 (2009).

18. M. F. Moescheid, Z. Lu, C. D. Soria, T. Quack, O. Puckelwaldt, N. Holroyd, P. Holzaepfel, S. Haeberlein, G. Rinaldi, M. Berriman, C. G. Grevelding, The retinoic acid family-like nuclear

receptor SmRAR identified by single-cell transcriptomics of ovarian cells controls oocyte differentiation in *Schistosoma mansoni*. *Nucleic Acids Res.* **53**, gkae1228 (2025).

19. Z. Lu, F. Sessler, N. Holroyd, S. Hahnel, T. Quack, M. Berriman, C. G. Grevelding, Schistosome sex matters: A deep view into gonad-specific and pairing-dependent transcriptomes reveals a complex gender interplay. *Sci. Rep.* **6**, 31150 (2016).

20. Z. Lu, F. Sessler, N. Holroyd, S. Hahnel, T. Quack, M. Berriman, C. G. Grevelding, A gene expression atlas of adult *Schistosoma mansoni* and their gonads. *Sci. Data* **4**, 170118 (2017).

21. B. J. Bolt, F. H. Rodgers, M. Shafie, P. J. Kersey, M. Berriman, K. L. Howe, Using WormBase ParaSite: An integrated platform for exploring helminth genomic data. *Methods Mol. Biol.* **1757**, 471–491 (2018).

22. L. A. Kelley, S. Mezulis, C. M. Yates, M. N. Wass, M. J. E. Sternberg, The Phyre2 web portal for protein modeling, prediction and analysis. *Nat. Protoc.* **10**, 845–858 (2015).

23. G. C. Webb, R. T. Baker, K. Fagan, P. G. Board, Localization of the human UbB polyubiquitin gene to chromosome band 17p11.1-17p12. *Am. J. Hum. Genet.* **46**, 308–315 (1990).

24. M. G. Reese, Application of a time-delay neural network to promoter annotation in the *Drosophila melanogaster* genome. *Comput. Chem.* **26**, 51–56 (2001).

25. S. B. Roberts, N. Segil, N. Heintz, Differential phosphorylation of the transcription factor Oct1 during the cell cycle. *Science* **253**, 1022–1026 (1991).

26. E. Wingender, TRANSFAC, TRANSPATH and CYTOMER as starting points for an ontology of regulatory networks. *In Silico Biol.* **4**, 55–61 (2004).

27. Y. Zhang, J. Huang, X. Li, C. Fang, L. Wang, Identification of functional transcriptional binding sites within chicken *Abcg2* gene promoter and screening its regulators. *Genes* **11**, 186 (2020).

28. N. I. Gershenzon, I. P. Ioshikhes, Synergy of human Pol II core promoter elements revealed by statistical sequence analysis. *Bioinformatics* **21**, 1295–1300 (2005).

29. V. X. Jin, G. A. C. Singer, F. J. Agosto-Pérez, S. Liyanarachchi, R. V. Davuluri, Genome-wide analysis of core promoter elements from conserved human and mouse orthologous pairs. *BMC Bioinformatics* **7**, 114 (2006).

30. H. Liu, H. Han, J. Li, L. Wong, DNAFSMiner: A web-based software toolbox to recognize two types of functional sites in DNA sequences. *Bioinformatics* **21**, 671–673 (2005).

31. G. E. Chalkley, C. P. Verrijzer, DNA binding site selection by RNA polymerase II TAFs: A TAF(II)250-TAF(II)150 complex recognizes the initiator. *EMBO J.* **18**, 4835–4845 (1999).

32. K. Cartharius, K. Frech, K. Grote, B. Klocke, M. Haltmeier, A. Klingenhoff, M. Frisch, M. Bayerlein, T. Werner, MatInspector and beyond: Promoter analysis based on transcription factor binding sites. *Bioinformatics* **21**, 2933–2942 (2005).

33. J. E. F. Butler, J. T. Kadonaga, The RNA polymerase II core promoter: A key component in the regulation of gene expression. *Genes Dev.* **16**, 2583–2592 (2002).

34. M. Kozak, At least six nucleotides preceding the AUG initiator codon enhance translation in mammalian cells. *J. Mol. Biol.* **196**, 947–950 (1987).

35. G. R. Wendt, M. L. Reese, J. J. Collins, SchistoCyte Atlas: A single-cell transcriptome resource for adult schistosomes. *Trends Parasitol.* **37**, 585–587 (2021).

36. Z. Lu, Y. Zhang, M. Berriman, A web portal for gene expression across all life stages of *Schistosoma mansoni*. bioRxiv 308213 [Preprint] (2018). https://doi.org/10.1101/308213.

37. A. R. Gruber, R. Lorenz, S. H. Bernhart, R. Neuböck, I. L. Hofacker, The Vienna RNA websuite. *Nucleic Acids Res.* **36**, W70–W74 (2008).

38. S. W. Cheng, E. C. Lynch, K. R. Leason, D. L. Court, B. A. Shapiro, D. I. Friedman, Functional importance of sequence in the stem-loop of a transcription terminator. *Science* **254**, 1205–1207 (1991).

39. V. Wippersteg, K. Kapp, W. Kunz, W. P. Jackstadt, H. Zahner, C. G. Grevelding, HSP70-controlled GFP expression in transiently transformed schistosomes. *Mol. Biochem. Parasitol.* **120**, 141–150 (2002).

40. V. Wippersteg, F. Ribeiro, S. Liedtke, J. R. Kusel, C. G. Grevelding, The uptake of Texas Red-BSA in the excretory system of schistosomes and its colocalisation with ER60 promoter-induced GFP in transiently transformed adult males. *Int. J. Parasitol.* **33**, 1139–1143 (2003).

41. G. Lamolle, A. Iriarte, H. Musto, Codon usage in the flatworm *Schistosoma mansoni* is shaped by the mutational bias towards A+T and translational selection, which increases GC-ending codons in highly expressed genes. *Mol. Biochem. Parasitol.* **247**, 111445 (2022).

42. Y. Chen, M. Cheng, X. Feng, X. Niu, H. Song, Y. Cao, Genome editing by CRISPR/Cas12 recognizing AT-rich PAMs in *Shewanella oneidensis* MR-1. *ACS Synth. Biol.* **11**, 2947–2955 (2022).

43. G. Sankaranarayanan, A. Coghlan, P. Driguez, M. E. Lotkowska, M. Sanders, N. Holroyd, A. Tracey, M. Berriman, G. Rinaldi, Large CRISPR-Cas-induced deletions in the oxamniquine resistance locus of the human parasite *Schistosoma mansoni*. *Wellcome Open Res.* **5**, 178 (2020).

44. M. Nasri, P. Mir, B. Dannenmann, D. Amend, T. Skroblyn, Y. Xu, K. Schulze-Osthoff, M. Klimiankou, K. Welte, J. Skokowa, Fluorescent labeling of CRISPR/Cas9 RNP for gene knockout in HSPCs and iPSCs reveals an essential role for GADD45b in stress response. *Blood Adv.* **3**, 63–71 (2019).

45. E. K. Brinkman, T. Chen, M. Amendola, B. van Steensel, Easy quantitative assessment of genome editing by sequence trace decomposition. *Nucleic Acids Res.* **42**, e168 (2014).

46. K. Clement, H. Rees, M. C. Canver, J. M. Gehrke, R. Farouni, J. Y. Hsu, M. A. Cole, D. R. Liu, J. K. Joung, D. E. Bauer, L. Pinello, CRISPResso2 provides accurate and rapid genome editing sequence analysis. *Nat. Biotechnol.* **37**, 224–226 (2019).

47. K. Labun, T. G. Montague, M. Krause, Y. N. T. Cleuren, H. Tjeldnes, E. Valen, CHOPCHOP v3: Expanding the CRISPR web toolbox beyond genome editing. *Nucleic Acids Res.* **47**, W171–W174 (2019).

48. J. Park, K. Lim, J.-S. Kim, S. Bae, Cas-analyzer: An online tool for assessing genome editing results using NGS data. *Bioinformatics* **33**, 286–288 (2017).

49. Y. Yu, Y. Guo, Q. Tian, Y. Lan, H. Yeh, M. Zhang, I. Tasan, S. Jain, H. Zhao, An efficient gene knock-in strategy using 5′-modified double-stranded DNA donors with short homology arms. *Nat. Chem. Biol.* **16**, 387–390 (2020).

50. S. C. Pan, The fine structure of the miracidium of *Schistosoma mansoni*. *J. Invertebr. Pathol.* **36**, 307–372 (1980).

51. T. P. Yoshino, J. R. Laursen, Production of *Schistosoma mansoni* daughter sporocysts from mother sporocysts maintained in synxenic culture with *Biomphalaria glabrata* embryonic (Bge) cells. *J. Parasitol.* **81**, 714–722 (1995).

52. K. Kapp, C. Coustau, V. Wippersteg, J. Jourdane, W. Kunz, C. G. Grevelding, Transplantation of in vitro-generated *Schistosoma mansoni* mother sporocysts into *Biomphalaria glabrata*. *Parasitol. Res.* **91**, 482–485 (2003).

53. J. Jourdane, A. Theron, *Schistosoma mansoni*: Cloning by microsurgical transplantation of sporocysts. *Exp. Parasitol.* **50**, 349–357 (1980).

54. E. Chernin, Maintenance in vitro of larval *Schistosoma mansoni* in tissues from the snail, *Australorbis glabratus*. *J. Parasitol.* **50**, 531–545 (1964).

55. E. Chernin, Observations on hearts explanted in vitro from the snail *Australorbis glabratus*. *J. Parasitol.* **49**, 353–364 (1963).

56. G. Mouahid, A. Rognon, R. de Carvalho Augusto, P. Driguez, K. Geyer, S. Karinshak, N. Luviano, V. Mann, T. Quack, K. Rawlinson, G. Wendt, C. Grunau, H. Moné, Transplantation of schistosome sporocysts between host snails: A video guide. *Wellcome Open Res.* **3**, 3 (2018).

57. M. Stitz, C. Chaparro, Z. Lu, V. J. Olzog, C. E. Weinberg, J. Blom, A. Goesmann, C. Grunau, C. G. Grevelding, Satellite-like W-elements: Repetitive, transcribed, and putative mobile genetic Factors with potential roles for biology and evolution of *Schistosoma mansoni*. *Genome Biol. Evol.* **13**, evab204 (2021).

58. W. Ittiprasert, V. H. Mann, S. E. Karinshak, A. Coghlan, G. Rinaldi, G. Sankaranarayanan, A. Chaidee, T. Tanno, C. Kumkhaek, P. Prangtaworn, M. M. Mentink-Kane, C. J. Cochran, P. Driguez, N. Holroyd, A. Tracey, R. Rodpai, B. Everts, C. H. Hokke, K. F. Hoffmann, M. Berriman, P. J. Brindley, Programmed genome editing of the omega-1 ribonuclease of the blood fluke, *Schistosoma mansoni*. *eLife* **8**, e41337 (2019).

59. X. Wang, Y. Tang, J. Lu, Y. Shao, X. Qin, Y. Li, L. Wang, D. Li, M. Liu, Characterization of novel cytochrome P450 2E1 knockout rat model generated by CRISPR/Cas9. *Biochem. Pharmacol.* **105**, 80–90 (2016).

60. S. Chaiyadet, S. Tangkawattana, M. J. Smout, W. Ittiprasert, V. H. Mann, R. Deenonpoe, P. Arunsan, A. Loukas, P. J. Brindley, T. Laha, Knockout of liver fluke granulin, Ov-grn-1, impedes malignant transformation during chronic infection with *Opisthorchis viverrini*. *PLOS Pathog.* **18**, e1010839 (2022).

61. P. Khampoosa, M. K. Jones, E. M. Lovas, T. Srisawangwong, T. Laha, S. Piratae, C. Thammasiri, A. Suwannatrai, B. Sripanidkulchai, V. Eursitthichai, S. Tesana, Light and electron microscopy observations of embryogenesis and egg development in the human liver fluke, *Opisthorchis viverrini* (Platyhelminthes, Digenea). *Parasitol. Res.* **110**, 799–808 (2012).

62. A. Born-Torrijos, A. S. Holzer, J. A. Raga, G. S. van Beest, A. Yoneva, Description of embryonic development and ultrastructure in miracidia of *Cardiocephaloides longicollis* (Digenea, Strigeidae) in relation to active host finding strategy in a marine environment. *J. Morphol.* **278**, 1137–1148 (2017).

63. H. You, J. U. Mayer, R. L. Johnston, H. Sivakumaran, S. Ranasinghe, V. Rivera, O. Kondrashova, L. T. Koufariotis, X. Du, P. Driguez, J. D. French, N. Waddell, M. G. Duke, W.

Ittiprasert, V. H. Mann, P. J. Brindley, M. K. Jones, D. P. McManus, CRISPR/Cas9-mediated genome editing of *Schistosoma mansoni* acetylcholinesterase. *FASEB J.* **35**, e21205 (2021).

64. X. Du, D. P. McManus, J. D. French, N. Collinson, H. Sivakumaran, S. R. MacGregor, C. E. Fogarty, M. K. Jones, H. You, CRISPR interference for sequence-specific regulation of fibroblast growth factor receptor A in *Schistosoma mansoni*. *Front. Immunol.* **13**, 1105719 (2022).

65. C. L. Diaz Soria, J. Lee, T. Chong, A. Coghlan, A. Tracey, M. D. Young, T. Andrews, C. Hall, B. L. Ng, K. Rawlinson, S. R. Doyle, S. Leonard, Z. Lu, H. M. Bennett, G. Rinaldi, P. A. Newmark, M. Berriman, Single-cell atlas of the first intra-mammalian developmental stage of the human parasite *Schistosoma mansoni*. *Nat. Commun.* **11**, 6411 (2020).

66. P. Kazemian, S.-Y. Yu, S. B. Thomson, A. Birkenshaw, B. R. Leavitt, C. J. D. Ross, Lipid-nanoparticle-based delivery of CRISPR/Cas9 genome-editing components. *Mol. Pharm.* **19**, 1669–1686 (2022).

67. D. A. Kuppers, J. Linton, S. Ortiz Espinosa, K. M. McKenna, A. Rongvaux, P. J. Paddison, Gene knock-outs in human CD34+ hematopoietic stem and progenitor cells and in the human immune system of mice. *PLOS ONE* **18**, e0287052 (2023).

68. D. Mazurov, L. Ramadan, N. Kruglova, Packaging and uncoating of CRISPR/Cas ribonucleoproteins for efficient gene editing with viral and non-viral extracellular nanoparticles. *Viruses* **15**, 690 (2023).

69. A. Bhandawat, V. Sharma, V. Rishi, J. K. Roy, Biolistic delivery of programmable nuclease (CRISPR/Cas9) in bread wheat. *Methods Mol. Biol.* **2124**, 309–329 (2020).

70. O. Heyers, A. K. Walduck, P. J. Brindley, W. Bleiss, R. Lucius, T. Dorbic, B. Wittig, B. H. Kalinna, *Schistosoma mansoni* miracidia transformed by particle bombardment infect *Biomphalaria glabrata* snails and develop into transgenic sporocysts. *Exp. Parasitol.* **105**, 174–178 (2003).

71. V. Wippersteg, K. Kapp, W. Kunz, C. G. Grevelding, Characterisation of the cysteine protease ER60 in transgenic *Schistosoma mansoni* larvae. *Int. J. Parasitol.* **32**, 1219–1224 (2002).

72. M. F. Moescheid, O. Puckelwaldt, M. Beutler, S. Haeberlein, C. G. Grevelding, Defining an optimal control for RNAi experiments with adult *Schistosoma mansoni*. *Sci. Rep.* **13**, 9766 (2023).

73. S. Khan, E. Sallard, Current and prospective applications of CRISPR-Cas12a in pluricellular organisms. *Mol. Biotechnol.* **65**, 196–205 (2023).

74. M. A. Moreno-Mateos, J. P. Fernandez, R. Rouet, C. E. Vejnar, M. A. Lane, E. Mis, M. K. Khokha, J. A. Doudna, A. J. Giraldez, CRISPR-Cpf1 mediates efficient homology-directed repair and temperature-controlled genome editing. *Nat. Commun.* **8**, 2024 (2017).

75. Z. Mao, M. Bozzella, A. Seluanov, V. Gorbunova, DNA repair by nonhomologous end joining and homologous recombination during cell cycle in human cells. *Cell Cycle* **7**, 2902–2906 (2008).

76. Z. Lu, T. Quack, S. Hahnel, V. Gelmedin, E. Pouokam, M. Diener, M. Hardt, G. Michel, N. Baal, H. Hackstein, C. G. Grevelding, Isolation, enrichment and primary characterisation of vitelline cells from *Schistosoma mansoni* obtained by the organ isolation method. *Int. J. Parasitol.* **45**, 663–672 (2015).

77. P. D. Ashton, R. Harrop, B. Shah, R. A. Wilson, The schistosome egg: Development and secretions. *Parasitology* **122**, 329–338 (2001).

78. E. A. Meuleman, P. J. Holzmann, R. C. Peet, The development of daughter sporocysts inside the mother sporocyst of *Schistosoma mansoni* with special reference to the ultrastructure of the body wall. *Z. Für Parasitenkd.* **61**, 201–212 (1980).

79. N. C. Hoch, Tissue specificity of DNA damage and repair. *Physiology* **38**, 231–241 (2023).

80. F. Fattah, E. H. Lee, N. Weisensel, Y. Wang, N. Lichter, E. A. Hendrickson, Ku regulates the non-homologous end joining pathway choice of DNA double-strand break repair in human somatic cells. *PLOS Genet.* **6**, e1000855 (2010).

81. M. Sato, S. Takabayashi, E. Akasaka, S. Nakamura, Recent advances and future perspectives of in vivo targeted delivery of genome-editing reagents to germ cells, embryos, and fetuses in mice. *Cells* **9**, 799 (2020).

82. T. Iyama, D. M. Wilson, DNA repair mechanisms in dividing and non-dividing cells. *DNA Repair (Amst.)* **12**, 620–636 (2013).

83. B. B. L. G. Lemmens, M. Tijsterman, DNA double-strand break repair in *Caenorhabditis elegans*. *Chromosoma* **120**, 1–21 (2011).

84. W. Bae, S. Hong, M. S. Park, H.-K. Jeong, M.-H. Lee, H.-S. Koo, Single-strand annealing mediates the conservative repair of double-strand DNA breaks in homologous recombination-defective germ cells of *Caenorhabditis elegans*. *DNA Repair (Amst.)* **75**, 18–28 (2019).

85. C. Dieterich, S. W. Clifton, L. N. Schuster, A. Chinwalla, K. Delehaunty, I. Dinkelacker, L. Fulton, R. Fulton, J. Godfrey, P. Minx, M. Mitreva, W. Roeseler, H. Tian, H. Witte, S.-P. Yang, R. K. Wilson, R. J. Sommer, The *Pristionchus pacificus* genome provides a unique perspective on nematode lifestyle and parasitism. *Nat. Genet.* **40**, 1193–1198 (2008).

86. T. Rillo-Bohn, R. Adilardi, T. Mitros, B. Avşaroğlu, L. Stevens, S. Köhler, J. Bayes, C. Wang, S. Lin, K. A. Baskevitch, D. S. Rokhsar, A. F. Dernburg, Analysis of meiosis in *Pristionchus pacificus* reveals plasticity in homolog pairing and synapsis in the nematode lineage. *eLife* **10**, e70990 (2021).

87. The C. elegans Sequencing Consortium, Genome sequence of the nematode *C. elegans*: A platform for investigating biology. *Science* **282**, 2012–2018 (1998).

88. J. S. Martin, N. Winkelmann, M. I. R. Petalcorin, M. J. McIlwraith, S. J. Boulton, RAD-51-dependent and -independent roles of a *Caenorhabditis elegans* BRCA2-related protein during DNA double-strand break repair. *Mol. Cell. Biol.* **25**, 3127–3139 (2005).

89. M. R. Lieber, The mechanism of human nonhomologous DNA end joining. *J. Biol. Chem.* **283**, 1–5 (2008).

90. G. Wendt, L. Zhao, R. Chen, C. Liu, A. J. O'Donoghue, C. R. Caffrey, M. L. Reese, J. J. Collins, A single-cell RNA-seq atlas of *Schistosoma mansoni* identifies a key regulator of blood feeding. *Science* **369**, 1644–1649 (2020).

91. S. Ozturk, N. Demir, DNA repair mechanisms in mammalian germ cells. *Histol. Histopathol.* **26**, 505–517 (2011).

92. H. You, M. K. Jones, D. J. Whitworth, D. P. McManus, Innovations and advances in Schistosome stem cell research. *Front. Immunol.* **12**, 599014 (2021).

93. W. T. Godbey, *Biotechnology and Its Applications (2nd Edition)* (Academic Press, 2021).

94. U. Ohler, G. Liao, H. Niemann, G. M. Rubin, Computational analysis of core promoters in the *Drosophila* genome. *Genome Biol.* **3**, RESEARCH0087 (2002).

95. T. Juven-Gershon, J. T. Kadonaga, Regulation of gene expression via the core promoter and the basal transcriptional machinery. *Dev. Biol.* **339**, 225–229 (2010).

96. S. U. Busek, M. Fantappie, L. C. Malaquias, R. A. Wilson, R. Corrêa-Oliveira, G. C. Oliveira, Cis-acting elements, CArG-, E-, CCAAT- and TATA-boxes may be involved in sexually regulated gene transcription in *Schistosoma mansoni*. *Mem. Inst. Oswaldo Cruz* **97** (Suppl. 1), 85–90 (2002).

97. S. M. Byrne, L. Ortiz, P. Mali, J. Aach, G. M. Church, Multi-kilobase homozygous targeted gene replacement in human induced pluripotent stem cells. *Nucleic Acids Res.* **43**, e21 (2015).

98. J.-P. Zhang, X.-L. Li, G.-H. Li, W. Chen, C. Arakaki, G. D. Botimer, D. Baylink, L. Zhang, W. Wen, Y.-W. Fu, J. Xu, N. Chun, W. Yuan, T. Cheng, X.-B. Zhang, Efficient precise knockin with a double cut HDR donor after CRISPR/Cas9-mediated double-stranded DNA cleavage. *Genome Biol.* **18**, 35 (2017).

99. T. Sakuma, S. Nakade, Y. Sakane, K.-I. T. Suzuki, T. Yamamoto, MMEJ-assisted gene knock-in using TALENs and CRISPR-Cas9 with the PITCh systems. *Nat. Protoc.* **11**, 118–133 (2016).

100. T. Sakuma, M. Takenaga, Y. Kawabe, T. Nakamura, M. Kamihira, T. Yamamoto, Homologous recombination-independent large gene cassette knock-in in CHO cells using TALEN and MMEJ-directed donor plasmids. *Int. J. Mol. Sci.* **16**, 23849–23866 (2015).

101. G. Li, X. Zhang, H. Wang, J. Mo, C. Zhong, J. Shi, R. Zhou, Z. Li, H. Yang, Z. Wu, D. Liu, CRISPR/Cas9-Mediated integration of large transgene into pig CEP112 locus. *G3 (Bethesda)* **10**, 467–473 (2020).

102. J. A. Gutierrez-Triana, T. Tavhelidse, T. Thumberger, I. Thomas, B. Wittbrodt, T. Kellner, K. Anlas, E. Tsingos, J. Wittbrodt, Efficient single-copy HDR by 5′ modified long dsDNA donors. *eLife* **7**, e39468 (2018).

103. Y.-Y. Jin, P. Zhang, L.-L. Liu, X. Zhao, X.-Q. Hu, S.-Z. Liu, Z.-K. Li, Q. Liu, J.-Q. Wang, D.-L. Hao, Z.-Q. Zhang, H.-Z. Chen, D.-P. Liu, Enhancing homology-directed repair efficiency with HDR-boosting modular ssDNA donor. *Nat. Commun.* **15**, 6843 (2024).

104. S. Qi, Y. Wang, Z. Liu, S. Wu, Y. Zhao, Y. Li, S. Deng, K. Yu, Z. Lian, Construction of a TAT-Cas9-EGFP site-specific integration eukaryotic cell line using efficient PEG10 modification. *Int. J. Mol. Sci.* **26**, 1331 (2025).

105. M. Berriman, B. J. Haas, P. T. LoVerde, R. A. Wilson, G. P. Dillon, G. C. Cerqueira, S. T. Mashiyama, B. Al-Lazikani, L. F. Andrade, P. D. Ashton, M. A. Aslett, D. C. Bartholomeu, G. Blandin, C. R. Caffrey, A. Coghlan, R. Coulson, T. A. Day, A. Delcher, R. DeMarco, A. Djikeng, T. Eyre, J. A. Gamble, E. Ghedin, Y. Gu, C. Hertz-Fowler, H. Hirai, Y. Hirai, R. Houston, A. Ivens, D. A. Johnston, D. Lacerda, C. D. Macedo, P. McVeigh, Z. Ning, G. Oliveira, J. P. Overington, J. Parkhill, M. Pertea, R. J. Pierce, A. V. Protasio, M. A. Quail, M.-A. Rajandream, J. Rogers, M. Sajid, S. L. Salzberg, M. Stanke, A. R. Tivey, O. White, D. L. Williams, J. Wortman, W. Wu, M. Zamanian, A. Zerlotini, C. M. Fraser-Liggett, B. G. Barrell, N. M. El-Sayed, The genome of the blood fluke *Schistosoma mansoni*. *Nature* **460**, 352–358 (2009).

106. B. Wang, J. Lee, P. Li, A. Saberi, H. Yang, C. Liu, M. Zhao, P. A. Newmark, Stem cell heterogeneity drives the parasitic life cycle of *Schistosoma mansoni*. *eLife* **7**, e35449 (2018).

107. D. P. McManus, D. W. Dunne, M. Sacko, J. Utzinger, B. J. Vennervald, X.-N. Zhou, Schistosomiasis. *Nat. Rev. Dis. Primer* **4**, 13 (2018).

108. P. J. Hotez, A. Kamath, Neglected tropical diseases in sub-saharan Africa: Review of their prevalence, distribution, and disease burden. *PLOS Negl. Trop. Dis.* **3**, e412 (2009).

109. F. Cobo, "8—*Schistosomiasis (bilharziasis)*" in *Imported Infectious Diseases*, F. Cobo, Ed. (Woodhead Publishing, 2014).

110. J. Huang, D. Rowe, P. Subedi, W. Zhang, T. Suelter, B. Valent, D. E. Cook, CRISPR-Cas12a induced DNA double-strand breaks are repaired by multiple pathways with different mutation profiles in *Magnaporthe oryzae*. *Nat. Commun.* **13**, 7168 (2022).

111. J. Kirchhoff, A. Schiermeyer, K. Schneider, R. Fischer, W. M. Ainley, S. R. Webb, H. Schinkel, S. Schillberg, Gene expression variability between randomly and targeted transgene integration events in tobacco suspension cell lines. *Plant Biotechnol. Rep.* **14**, 451–458 (2020).

112. Z. C. Stevenson, M. J. Moerdyk-Schauwecker, B. Jamison, P. C. Phillips, Rapid self-selecting and clone-free integration of transgenes into engineered CRISPR safe harbor locations in *Caenorhabditis elegans*. *G3 (Bethesda)* **10**, 3775–3782 (2020).

113. S. Riesenberg, P. Kanis, D. Macak, D. Wollny, D. Düsterhöft, J. Kowalewski, N. Helmbrecht, T. Maricic, S. Pääbo, Efficient high-precision homology-directed repair-dependent genome editing by HDRobust. *Nat. Methods* **20**, 1388–1399 (2023).

114. A. H. Straume, E. Kjærner-Semb, K. Ove Skaftnesmo, H. Güralp, L. Kleppe, A. Wargelius, R. B. Edvardsen, Indel locations are determined by template polarity in highly efficient in vivo CRISPR/Cas9-mediated HDR in Atlantic salmon. *Sci. Rep.* **10**, 409 (2020).

115. K. L. Howe, B. J. Bolt, M. Shafie, P. Kersey, M. Berriman, WormBase ParaSite—A comprehensive resource for helminth genomics. *Mol. Biochem. Parasitol.* **215**, 2–10 (2017).

116. R. A. Anderson, S. Kato, R. D. Camerini-Otero, A pattern of partially homologous recombination in mouse L cells. *Proc. Natl. Acad. Sci. U.S.A.* **81**, 206–210 (1984).

117. X. Yao, X. Wang, X. Hu, Z. Liu, J. Liu, H. Zhou, X. Shen, Y. Wei, Z. Huang, W. Ying, Y. Wang, Y.-H. Nie, C.-C. Zhang, S. Li, L. Cheng, Q. Wang, Y. Wu, P. Huang, Q. Sun, L. Shi, H. Yang, Homology-mediated end joining-based targeted integration using CRISPR/Cas9. *Cell Res.* **27**, 801–814 (2017).

118. M. Li, J. Zhang, Q. Bai, L. Fang, H. Song, Y. Cao, Non-homologous end joining-mediated insertional mutagenesis reveals a novel target for enhancing fatty alcohols production in *Yarrowia lipolytica*. *Front. Microbiol.* **13**, 898884 (2022).

119. J. Davis, M. Maillet, J. M. Miano, J. D. Molkentin, Lost in Transgenesis: A users guide for Genetically manipulating the mouse in cardiac research. *Circ. Res.* **111**, 761–777 (2012).

120. N. Haruyama, A. Cho, A. B. Kulkarni, Overview: Engineering transgenic constructs and mice. *Curr. Protoc. Cell Biol.* **42**, 19.10.1–19.10.9 (2009).

121. V. Thermes, C. Grabher, F. Ristoratore, F. Bourrat, A. Choulika, J. Wittbrodt, J.-S. Joly, *I-SceI* meganuclease mediates highly efficient transgenesis in fish. *Mech. Dev.* **118**, 91–98 (2002).

122. K. Terasaki, N. Moriyama-Gonda, Y. Noda, Abnormal spermatogenesis in the common liver fluke (*Fasciola* sp.) from Japan and Korea. *J. Vet. Med. Sci.* **60**, 1305–1309 (1998).

123. S. Svitashev, J. K. Young, C. Schwartz, H. Gao, S. C. Falco, A. M. Cigan, Targeted mutagenesis, precise gene editing, and site-specific gene insertion in maize using Cas9 and guide RNA. *Plant Physiol.* **169**, 931–945 (2015).

124. J. Wang, R. Chen, J. J. Collins III, Systematically improved in vitro culture conditions reveal new insights into the reproductive biology of the human parasite *Schistosoma mansoni*. *PLOS Biol.* **17**, e3000254 (2019).

125. Y. You, X. Chen, L. Huo, L. Chen, G. Chen, M. Gu, C. Yi, J. Wang, W. Hu, An improved medium for in vitro studies of female reproduction and oviposition in *Schistosoma japonicum*. *Parasit. Vectors* **17**, 116 (2024).

126. X. Li, O. Weth, S. Haeberlein, C. G. Grevelding, Molecular characterization of Smtdc-1 and Smddc-1 discloses roles as male-competence factors for the sexual maturation of *Schistosoma mansoni* females. *Front. Cell. Infect. Microbiol.* **13**, 1173557 (2023).

127. C. L. Diaz Soria, T. Attenborough, Z. Lu, S. Fontenla, J. Graham, C. Hall, S. Thompson, T. G. R. Andrews, K. A. Rawlinson, M. Berriman, G. Rinaldi, Single-cell transcriptomics of the human parasite *Schistosoma mansoni* first intra-molluscan stage reveals tentative tegumental and stem-cell regulators. *Sci. Rep.* **14**, 5974 (2024).

128. A. Raveux, S. Vandormael-Pournin, M. Cohen-Tannoudji, Optimization of the production of knock-in alleles by CRISPR/Cas9 microinjection into the mouse zygote. *Sci. Rep.* **7**, 42661 (2017).

129. T. Thumberger, T. Tavhelidse-Suck, J. A. Gutierrez-Triana, A. Cornean, R. Medert, B. Welz, M. Freichel, J. Wittbrodt, Boosting targeted genome editing using the hei-tag. *Elife* **11**, e70558 (2022).

130. B. Farboud, Targeted genome editing in *Caenorhabditis elegans* using CRISPR/Cas9. *Wiley Interdiscip. Rev. Dev. Biol.* **6**, e287 (2017).

131. D. N. Clarke, A. Kane, M. Perillo, C. J. Lowe, S. Z. Swartz, VitelloTag: A tool for high-throughput cargo delivery into oocytes. *Development* **151**, dev202857 (2024).

132. M. Lebouvier, P. Miramón-Puértolas, P. R. H. Steinmetz, Evolutionarily conserved aspects of animal nutrient uptake and transport in sea anemone vitellogenesis. *Curr. Biol.* **32**, 4620–4630.e5 (2022).

133. S. Mukherjee, The United States Food and Drug Administration (FDA) regulatory response to combat neglected tropical diseases (NTDs): A review. *PLOS Negl. Trop. Dis.* **17**, e0011010 (2023).

134. C. H. Hokke, A. M. Deelder, K. F. Hoffmann, M. Wuhrer, Glycomics-driven discoveries in schistosome research. *Exp. Parasitol.* **117**, 275–283 (2007).

135. E. L. Houlder, L. F. da Silva, A. van Diepen, M. S. Amaral, R. A. Wilson, C. H. Hokke, M. Roestenberg, W. A. M. Bakker, Pre-clinical studies of *Schistosoma mansoni* vaccines: A scoping review. *PLoS Negl. Trop. Dis.* **19**, e0012956 (2025).

136. M. M. Eissa, C. A. Ismail, M. Z. El-Azzouni, A. A. Ghazy, M. A. Hadi, Immuno-therapeutic potential of *Schistosoma mansoni* and *Trichinella spiralis* antigens in a murine model of colon cancer. *Invest. New Drugs* **37**, 47–56 (2019).

137. K. S. Singh, S. Bharti, B. A. Rosa, M. Bigham, S. C. Uzoechi, Y.-J. Choi, J. C. Martin, D. Kemper, S. P. Djuranovic, D. A. Pickering, R. Ryan, B. K. Bracken, M. E. Bottazzi, E. C. Carnes, W. Ittiprasert, M. Moyle, P. J. Brindley, A. Loukas, S. Djuranovic, M. Mitreva, Transgenic hookworm secretes anti-tetrodotoxin human single chain antibody. *Nat. Commun.* **17**, 4691 (2026).

138. S. N. Alwan, A. B. Taylor, J. Rhodes, M. Tidwell, S. F. McHardy, P. T. LoVerde, Oxamniquine derivatives overcome Praziquantel treatment limitations for Schistosomiasis. *PLOS Pathog.* **19**, e1011018 (2023).

139. A. A. Da'dara, P. J. Skelly, Gene suppression in schistosomes using RNAi. *Methods Mol. Biol.* **1201**, 143–164 (2015).

140. S. G. Gava, N. C. Tavares, A. C. M. Salim, F. M. G. de Araújo, G. Oliveira, M. M. Mourão, *Schistosoma mansoni*: Off-target analyses using nonspecific double-stranded RNAs as control for RNAi experiments in schistosomula. *Exp. Parasitol.* **177**, 98–103 (2017).

141. M. F. Moescheid, "Identification and functional characterization of nuclear receptors with roles in the reproductive development and approaches to establish genome editing in *Schistosoma mansoni*," thesis, Justus Liebig University Giessen, Giessen, Germany (2024).

142. K. L. Joyce, W. Morgan, R. Greenberg, M. G. Nair, Using eggs from *Schistosoma mansoni* as an in vivo model of helminth-induced lung inflammation. *J. Vis. Exp.* **64**, e3905 (2012).

143. W. Ittiprasert, A. Miller, Bge Cell Culture (Biomedical Research Institute, 2014); https://afbr-bri.org/schistosomiasis/standard-operating-procedures/biomphalaria-glabrata-embryonic-cell-bge-culture/ [accessed 09 June 2026].

144. M. Voge, J. S. Seidel, Transformation in vitro of miracidia of *Schistosoma mansoni* and *S. japonicum* into young sporocysts. *J. Parasitol.* **58**, 699–704 (1972).

145. H. Vogel, H. Vogel, Uber Entwicklung, Lebensdauer Tod der Eier von *Bilharzia japonica* im Wirtsgewebe [The development, lifespan and death of *Bilharzia japonica* eggs in host tissue]. *Dtsch. Tropenmedizinische Z* **46**, 57–91 (1942).

146. B. P. Cormack, R. H. Valdivia, S. Falkow, FACS-optimized mutants of the green fluorescent protein (GFP). *Gene* **173**, 33–38 (1996).

147. G. E. Crooks, G. Hon, J.-M. Chandonia, S. E. Brenner, WebLogo: A sequence logo generator. *Genome Res.* **14**, 1188–1190 (2004).

148. A. V. Protasio, I. J. Tsai, A. Babbage, S. Nichol, M. Hunt, M. A. Aslett, N. De Silva, G. S. Velarde, T. J. C. Anderson, R. C. Clark, C. Davidson, G. P. Dillon, N. E. Holroyd, P. T. LoVerde, C. Lloyd, J. McQuillan, G. Oliveira, T. D. Otto, S. J. Parker-Manuel, M. A. Quail, R. A. Wilson, A. Zerlotini, D. W. Dunne, M. Berriman, A systematically improved high quality genome and transcriptome of the human blood fluke *Schistosoma mansoni*. *PLOS Negl. Trop. Dis.* **6**, e1455 (2012).

149. R. Lorenz, S. H. Bernhart, C. Hönerzu Siederdissen, H. Tafer, C. Flamm, P. F. Stadler, I. L. Hofacker, ViennaRNA Package 2.0. *Algorithms Mol. Biol.* **6**, 26 (2011).

150. J. J. Collins, X. Hou, E. V. Romanova, B. G. Lambrus, C. M. Miller, A. Saberi, J. V. Sweedler, P. A. Newmark, Genome-wide analyses reveal a role for peptide hormones in planarian germline development. *PLOS Biol.* **8**, e1000509 (2010).

151. D. G. Gibson, L. Young, R.-Y. Chuang, J. C. Venter, C. A. Hutchison, H. O. Smith, Enzymatic assembly of DNA molecules up to several hundred kilobases. *Nat. Methods* **6**, 343–345 (2009).

152. T. G. Montague, J. M. Cruz, J. A. Gagnon, G. M. Church, E. Valen, CHOPCHOP: A CRISPR/Cas9 and TALEN web tool for genome editing. *Nucleic Acids Res.* **42**, W401–W407 (2014).

153. S. Bae, J. Park, J.-S. Kim, Cas-OFFinder: A fast and versatile algorithm that searches for potential off-target sites of Cas9 RNA-guided endonucleases. *Bioinformatics* **30**, 1473–1475 (2014).

154. K. Murugan, A. S. Seetharam, A. J. Severin, D. G. Sashital, CRISPR-Cas12a has widespread off-target and dsDNA-nicking effects. *J. Biol. Chem.* **295**, 5538–5553 (2020).

155. K. A. V. Kohabir, J. Linthorst, L. O. Nooi, R. Brouwer, R. M. F. Wolthuis, E. A. Sistermans, Synthetic mismatches enable specific CRISPR-Cas12a-based detection of genome-wide SNVs tracked by ARTEMIS. *Cell Rep. Methods* **4**, 100912 (2024).

156. A. O'Brien, D. C. Bauer, G. Burgio, Predicting CRISPR-Cas12a guide efficiency for targeting using machine learning. *PLOS ONE* **18**, e0292924 (2023).

157. E. Slaman, L. Kottenhagen, W. de Martines, G. C. Angenent, R. A. de Maagd, Comparison of Cas12a and Cas9-mediated mutagenesis in tomato cells. *Sci. Rep.* **14**, 4508 (2024).

158. D. Modrzejewski, F. Hartung, H. Lehnert, T. Sprink, C. Kohl, J. Keilwagen, R. Wilhelm, Which factors affect the occurrence of off-target effects caused by the use of CRISPR/Cas: A systematic review in plants. *Front. Plant Sci.* **11**, 574959 (2020).

159. L. M. Drepanos, S. Srikanth, E. G. Kaplan, S. T. Shah, B. E. Velasco, S. Merzouk, J. G. Doench, Balancing off-target and on-target considerations for optimized Cas9 CRISPR knockout library design. bioRxiv 672375 [Preprint] (2025). https://doi.org/10.1101/2025.08.26.672375.

160. C. Han-Min, W. Yun-Fang, L. So, A study of ultrastructure of egg shell of *Schistosoma japonicum*: I. Transmission electron microscopic observation of *S. japonicum* egg. *Ann. Parasitol. Hum. Comp.* **57**, 345–352 (1982).

161. E. K. Brinkman, B. van Steensel, Rapid quantitative evaluation of CRISPR genome editing by TIDE and TIDER. *Methods Mol. Biol.* **1961**, 29–44 (2019).

162. K. Zou, F. Wang, Z. Zhang, Y. Zhou, P. Li, D. Wang, M. Zhu, C. Jia, Z. Wei, Optimized CRISPR/Cas9 system for gene knockout in chicken DF1 cells. *Poult. Sci.* **102**, 102970 (2023).

163. K. Matsubara, S. Ueda, J. Yamamoto, S. Iwai, N. A. Shioi, A. Takedachi, I. Kuraoka, Structure-specific DNA endonuclease T7 endonuclease I cleaves DNA containing UV-induced DNA lesions. *J. Biochem.* **176**, 35–42 (2024).

164. D. M. J. Lilley, Holliday junction-resolving enzymes—Structures and mechanisms. *FEBS Lett.* **591**, 1073–1082 (2017).

165. A.-C. Déclais, J. M. Fogg, A. D. J. Freeman, F. Coste, J. M. Hadden, S. E. V. Phillips, D. M. J. Lilley, The complex between a four-way DNA junction and T7 endonuclease I. *EMBO J.* **22**, 1398–1409 (2003).

166. N. A. Peterson, C. H. Hokke, A. M. Deelder, T. P. Yoshino, Glycotope analysis in miracidia and primary sporocysts of *Schistosoma mansoni*: Differential expression during the miracidium- to sporocyst transformation. *Int. J. Parasitol.* **12**, 1331–1344 (2009).
